# Small extracellular vesicles from young plasma reverse age-related functional declines by improving mitochondrial energy metabolism

**DOI:** 10.1038/s43587-024-00612-4

**Published:** 2024-04-16

**Authors:** Xiaorui Chen, Yang Luo, Qing Zhu, Jingzi Zhang, Huan Huang, Yansheng Kan, Dian Li, Ming Xu, Shuohan Liu, Jianxiao Li, Jinmeng Pan, Li Zhang, Yan Guo, Binghao Wang, Guantong Qi, Zhen Zhou, Chen-Yu Zhang, Lei Fang, Yanbo Wang, Xi Chen

**Affiliations:** 1 Center for Reproductive Medicine and Department of Andrology, Nanjing Drum Tower Hospital, State Key Laboratory of Pharmaceutical Biotechnology, Jiangsu Engineering Research Center for MicroRNA Biology and Biotechnology, NJU Advanced Institute of Life Sciences (NAILS), School of Life Sciences, Nanjing University, Nanjing, China; 2 Jiangsu Key Laboratory of Molecular Medicine, Medical School of Nanjing University, Nanjing, China; 3 Chemistry and Biomedicine Innovation Center (ChemBIC), Nanjing University, Nanjing, China; 4 Department of Pediatrics, The First Affiliated Hospital of Nanjing Medical University, Nanjing, China; 5 Institute of Systems, Molecular and Integrative Biology, School of Life Sciences, University of Liverpool, Liverpool, UK; 6 Research Unit of Extracellular RNA, Chinese Academy of Medical Sciences, Nanjing, China; 7 Institute of Artificial Intelligence Biomedicine, Nanjing University, Nanjing, China

**Keywords:** miRNAs, Ageing

## Abstract

Recent investigations into heterochronic parabiosis have unveiled robust rejuvenating effects of young blood on aged tissues. However, the specific rejuvenating mechanisms remain incompletely elucidated. Here we demonstrate that small extracellular vesicles (sEVs) from the plasma of young mice counteract pre-existing aging at molecular, mitochondrial, cellular and physiological levels. Intravenous injection of young sEVs into aged mice extends their lifespan, mitigates senescent phenotypes and ameliorates age-associated functional declines in multiple tissues. Quantitative proteomic analyses identified substantial alterations in the proteomes of aged tissues after young sEV treatment, and these changes are closely associated with metabolic processes. Mechanistic investigations reveal that young sEVs stimulate PGC-1α expression in vitro and in vivo through their miRNA cargoes, thereby improving mitochondrial functions and mitigating mitochondrial deficits in aged tissues. Overall, this study demonstrates that young sEVs reverse degenerative changes and age-related dysfunction, at least in part, by stimulating PGC-1α expression and enhancing mitochondrial energy metabolism.

## Abstract (web summary)

Circulating factors have an important role in aging. Here the authors show that small extracellular vesicles derived from young plasma rejuvenate whole-body physiology in aged mice, at least in part, by stimulating PGC-1α expression and improving mitochondrial energy metabolism.

## Main

Aging is an inevitable, time-dependent process that eventually limits the capacity of cells to maintain efficient homeostasis and repair mechanisms, resulting in a general decline in physiological functions throughout the body. The chronic diseases associated with aging, such as cardiovascular diseases, diabetes, neurodegenerative diseases and cancer, place a tremendous burden on the healthcare system^1^. Hence, understanding the fundamental mechanisms of aging and developing strategies to combat aging and age-related diseases are greatly needed.

Mechanistically, the aging process is predominantly attributed to the progressive accumulation of stochastic damages in cells, organelles and macromolecules. The exact mechanisms underlying the damage are not yet fully understood but likely consist of several cell-autonomous processes, including genomic instability, telomere attrition, epigenetic alterations, loss of proteostasis, deregulated nutrient sensing, mitochondrial dysfunction, cellular senescence and stem cell exhaustion^2^. However, non-cell-autonomous mechanisms, especially the altered interactions among cells, tissues and organs, are also critically important for aging^3^. The efficient interaction between tissues and organs, facilitated by environmental factors present in the bloodstream, is vital for maintaining the health status of the whole body. Thus, age-related deterioration is also thought to arise from impaired tissue communication, with molecules circulating in the bloodstream serving as principal contributors^4^. Recent studies demonstrated that blood from young mice in heterochronic parabiosis—a surgical procedure that joins the circulation systems of two animals of different ages together—rejuvenated brain, heart, liver, pancreas, kidney, skeletal muscle and bone of aged mice^5^. Several studies further showed that plasma infusion recapitulates the phenotypes conferred by blood exchange in heterochronic parabiosis models, which decouples the effects of cellular contributions from transferred plasma factors^6,7^. Since then, great efforts have been devoted to identifying the soluble factors in plasma responsible for reversing age-related impairments. To date, several rejuvenation factors (also referred to as blood-borne factors, pro-youth factors or anti-aging factors) present in young plasma have been described^8–10^. However, the precise mechanisms underlying the action of these soluble plasma factors are not fully understood.

In the past decade, extracellular vesicle (EV)-mediated intercellular communication has added a new dimension to our knowledge of genetic information transfer and homeostasis maintenance in multicellular organisms. EVs, a heterogeneous group of nanosized membranous vesicles, are composed of a protein-rich lipid bilayer and the enclosed proteins and RNAs derived from EV-producing cells^11^. EVs circulate throughout the body via blood circulation and act as messengers of intercellular communication networks, allowing exchange of their cargoes between source cells and target cells^11^. Because the blood contains numerous bioactive EVs and EVs exert pleiotropic effects on whole-body physiology and homeostasis, blood-borne EVs may mediate the aging process through a non-cell-autonomous mechanism and may explain the rejuvenating effects of young blood. In this study, we focused on small extracellular vesicles (sEVs, <200 nm) and explored their rejuvenating effects by repeated administration of sEVs derived from young mice or humans to aged mice. We further elucidated the molecular basis underlying the critical roles of young sEVs in reversing age-related impairments and degenerative changes using in vivo and in vitro models.

## Results

### Long-term effects of young sEVs on lifespan and frailty

We first established a standard protocol for purifying sEVs from the plasma of young (2 months) and aged (20 months) male mice. Nanoparticle tracking analysis (NTA) revealed that the sEVs were present in young and aged plasma at a same order of magnitude (1.7 × 10^9^ particles per milliliter in young plasma versus 1.04 × 10^9^ particles per milliliter in aged plasma) and at a similar particle size (peaking at ~100 nm) (Supplementary Fig. 1a). Transmission electron microscopy (TEM) confirmed that the purified young sEVs exhibited the characteristic morphology and size of sEVs (Supplementary Fig. 1b). Immunoblotting analysis showed that the purified young and aged sEVs were highly enriched for classical sEV markers (CD9, CD63, Alix and TSG101) but devoid of the major plasma protein albumin and the endoplasmic reticulum protein calnexin (Supplementary Fig. 1c), indicating that the isolated sEVs were not contaminated by plasma and intracellular components. To simulate the natural concentration of sEVs in plasma, the purified sEVs were dissolved in PBS and adjusted to a concentration of 1.80 μg of total protein per microliter.

**Fig. 1:**
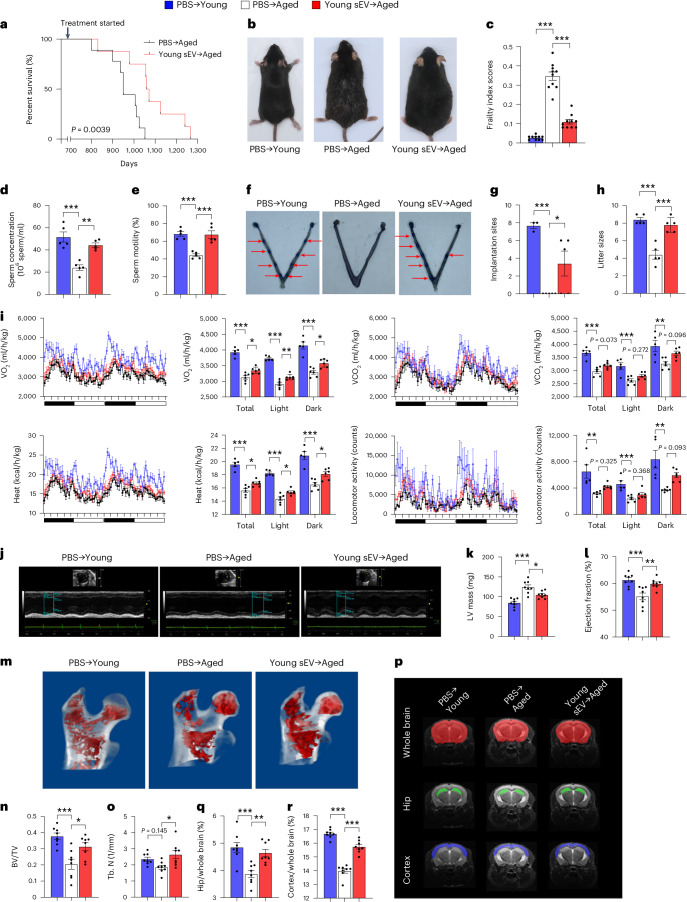
Long-term effects of young sEV injection on the lifespan and whole-body physiology of aged mice. Aged male mice (20 months) were intravenously injected with 200 μl of PBS or young sEVs (2 months) once a week, and they were monitored to determine either the survival time or whole-body physiology. Young male mice (2 months) were simultaneously injected with PBS to serve as a control group. **a**, Kaplan–Meier survival curves in each group (*n* = 8–9). **b**, Representative images of mice in each group after 7 months of treatment. **c**, Mean frailty index scores in each group after 4 months of treatment (*n* = 10). **d**,**e**, Sperm counts and motility in each group (*n* = 5). **f**,**g**, The number of implantation sites visible as blue bands in the uterus of female mice that were mated with the male mice from each group. Representative images (red arrows indicate implantation sites) and quantitative data (*n* = 3 for PBS → Young; *n* = 5 for else) are shown. **h**, The number of offspring sired by the male mice in each group (*n* = 5). **i**, Indirect calorimetry measurements of O_2_ consumption, CO_2_ release, heat production and locomotor activity in each group (*n* = 5 for PBS → Young; *n* = 6 for else). **j**–**l**, Echocardiographic measurements of cardiac dimensions and indices of cardiac function in each group. Representative M-mode echocardiographs and quantitative values of LV mass and EF (*n* = 8) are shown. **m**–**o**, Micro-CT analysis of the trabecular microarchitecture of the proximal femur in each group. Representative 3D images of the proximal femur and quantitative values of BV/TV and Tb.N (*n* = 8) are shown. **p**–**r**, MRI-based morphometric analyses of the hippocampus and cortex in each group. A representative MRI scan of a slice is shown, and the volume ratios (hippocampus/whole brain and cortex/whole brain) were calculated (*n* = 8). Significance was determined using the log-rank test in **a** and using one-way ANOVA followed by Dunnett’s multiple comparison test in **c**–**e**, **g**–**i**, **k**, **l**, **n**, **o**, **q** and **r**. *P < 0.05, **P < 0.01 and ***P < 0.005. Source data

Next, we determined whether the purified sEVs derived from young mouse plasma possess the capability to extend the lifespan of aged mice. An equal volume (200 μl) of PBS or young sEVs (1.80 μg of total protein per microliter) was intravenously injected into aged male mice (20 months) once a week until death. Survival analysis showed that weekly injection of young sEVs significantly extended the median lifespan of aged mice by 12.42% (34.4 months for young sEV-injected mice versus 30.6 months for PBS-injected mice) (Fig. 1a). Moreover, young-sEV-treated aged mice generally maintained a much healthier appearance than the age-matched control mice treated with PBS. Specifically, although young mice exhibited black and glossy hair on the dorsal back skin and aged mice displayed gray hair and patches of hair loss, injection of young sEVs substantially prevented hair loss in aged mice (Fig. 1b).

Frailty is an aging-related syndrome characterized by decline in many physiological systems, leading to loss of homeostasis and vulnerability to adverse health outcomes^12^. Previous studies established a non-invasive frailty index to quantify the frailty status of aged mice through clinical assessment of deficit accumulation^13^. With the use of this objective metric, we determined the frailty index scores in young-sEV-treated aged mice and calculated their biological age (an estimated age based on the extent of physiological degeneration) independent of their chronological age (defined by birth date). As anticipated, the average frailty index score was much higher in aged mice than in young mice (Fig. 1c). However, treatment with young sEVs markedly reduced the frailty index in aged mice (Fig. 1c), resulting in an estimate of biological age of approximately 15.1 months for these aged mice compared to their chronological age of 24 months. Particularly, although all clinical signs of deficits were alleviated in young-sEV-treated aged mice, the most obviously reduced deficits were observed in integument and the physical/musculoskeletal system (Supplementary Table 1).

### Long-term effects of young sEVs on whole-body physiology

To assess the long-term rejuvenating effects of young sEVs on distinct tissues and organs, aged male mice were intravenously injected with 200 μl of PBS or young sEVs once a week (Supplementary Fig. 2a). Because advanced male age negatively impacts fertility and is closely related to low sperm count, reduced sperm motility and high DNA fragmentation^14^, we first evaluated the rejuvenating effects of young sEVs on sperm quality and male fertility. Compared to young mice, aged mice exhibited markedly lower testosterone concentrations in the plasma and testis (Supplementary Fig. 2b,c), indicating the presence of age-related testosterone secretion insufficiency. However, treatment with young sEVs significantly increased circulating and intratesticular testosterone concentrations in aged mice (Supplementary Fig. 2b,c). Both total sperm counts and the percentage of sperms displaying forward progressive movement were substantially reduced in aged mice compared to young mice, but young sEV injection significantly improved sperm counts and enhanced sperm motility in aged mice (Fig. 1d,e). When the susceptibility of sperm DNA to denaturation was evaluated using a sperm chromatin structure assay, there was a notable increase in sperm DNA fragmentation observed in aged mice (Supplementary Fig. 2d,e). However, treatment with young sEVs significantly improved the integrity of sperm chromatin and reduced the proportion of sperm DNA fragmentation in aged mice (Supplementary Fig. 2d,e). To evaluate the fertility of young-sEV-treated aged mice, sexually mature female mice of proven fertility were mated with aged or young male mice for 12 h, and then the embryo implantation was examined using vascular blue dye injection at 4.5 days post-coitus (dpc) to visualize implantation sites in the uterus. Young males resulted in an average of 7.7 ± 0.3 implantation sites per pregnant female, whereas aged males failed to establish visible implantation sites in the uterus (Fig. 1f,g). Young sEV injection substantially alleviated the male fertility defects in aged mice, improving the success rate of embryo implantation to 3.4 ± 1.4 sites per pregnant female (Fig. 1f,g). In addition, we conducted a continuous mating experiment using proven fertile females paired with aged or young males to determine the reproductive outcomes of young-sEV-treated aged mice. After a 1-month mating period, young males produced an average of 8.4 ± 1 pups per litter, whereas aged males produced an average of 4.4 ± 1 pups per litter (Fig. 1h). After the injection of young sEVs, aged males produced nearly equal numbers of pups (7.8 ± 1 pups per litter) as young males (Fig. 1h).

Second, we employed an indirect calorimetry system to assess the metabolic health of mice. Consistent with previous reports that energy expenditure and locomotor activity decline with the aging process^15^, aged mice had a lower oxygen consumption (VO_2_) and carbon dioxide production (VCO_2_) and exhibited decreased locomotor activity and heat production in both dark and light cycles (Fig. 1i). Young-sEV-treated aged mice exhibited consistently higher VO_2_ and VCO_2_ and displayed elevated locomotor activity and heat production than age-matched control mice treated with PBS (Fig. 1i). These results suggest that the injection of young sEVs into aged mice partially restored their metabolic health to the level of young mice.

Third, we examined the cardiac performance of mice using echocardiography, as progressive left ventricular (LV) hypertrophy and systolic/diastolic dysfunction are hallmarks of cardiac aging^16^. In echocardiography, cardiac aging is usually reflected by an increased LV mass, a reduction in the ejection fraction (EF) and fractional shortening (FS) and an increase in LV volumes at end-diastolic and end-systolic phases (LV Vol;d and LV Vol;s, respectively)^17^. As anticipated, aged mice exhibited significant decreases in EF and FS and increases in LV mass, LV Vol;d and LV Vol;s compared to young mice (Fig. 1j–l and Supplementary Fig. 2f–h), indicating the prevalence of LV hypertrophy and impaired ventricular filling and myocardial contractility in aged mice. However, after the injection of young sEVs, an apparent improvement in the echocardiography parameters was observed in aged mice (Fig. 1j–l and Supplementary Fig. 2f–h).

Fourth, we evaluated whether young sEVs could protect aged mice from accelerated bone loss. On microcomputed tomography (micro-CT), age-related bone loss and architecture deterioration are usually manifested as a decrease in the number of trabeculae of cancellous bone, an increase in intertrabecular distance and a loss of trabecular connectivity^18^. Accordingly, micro-CT images showed that aged mice had a porous trabecular bone structure at the proximal femur, whereas young mice had a well-structured and dense trabecular bone (Fig. 1m). Quantitative analyses revealed that the bone volume per tissue volume (BV/TV), trabecular number (Tb.N) and trabecular thickness (Tb.Th) were lower and the trabecular separation (Tb.Sp) was higher in aged mice than in young mice (Fig. 1n,o and Supplementary Fig. 2i,j). Treatment with young sEVs substantially repaired the bone architecture in aged mice, bringing the trabecular bone parameters to a similar level as observed in young mice (Fig. 1m–o and Supplementary Fig. 2i,j).

Finally, we employed magnetic resonance imaging (MRI) to monitor the volumetric alterations in the hippocampal and cortical brain areas in aged mice. The hippocampus and cerebral cortex play key roles in learning, memory and executive functions, and these brain regions are particularly vulnerable to aging at the morphological level, which is characterized by progressive hippocampal and cortical atrophy resulting from neuronal shrinkage and loss^19^. Both the absolute hippocampal volume and the ratio of hippocampal volume to whole-brain volume were reduced in aged mice compared to young mice, whereas the young sEV injection significantly attenuated hippocampal loss in aged mice (Fig. 1p,q and Supplementary Fig. 2k–n). Similarly, apparent cortical atrophy was observed in aged mice, but young sEV injection mitigated the degree of atrophy and alleviated the decrease in cortical volume (Fig. 1p,r and Supplementary Fig. 2k–m,o). Taken together, the above results suggest that young sEVs have the capacity to revitalize the morphology and performance of aged tissues and organs to a younger state.

### Short-term effects of young sEVs on cognition and endurance

**Fig. 2:**
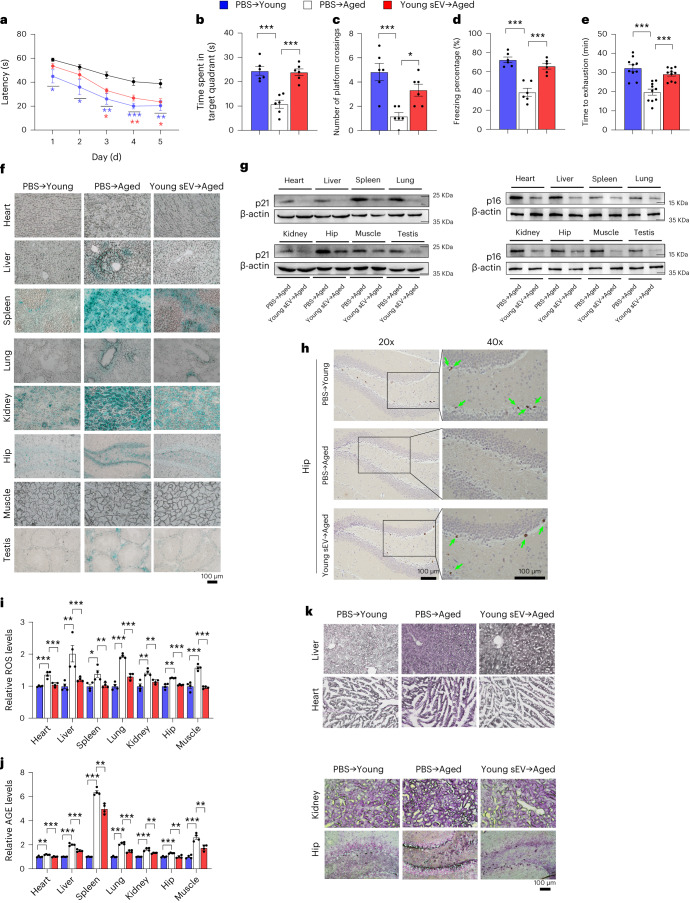
Short-term effects of young sEV injection on memory ability, endurance performance and senescent phenotypes of aged mice. Aged male mice (21 months) were intravenously injected with 200 μl of PBS or young sEVs (1.80 μg of total protein per microliter, from 2-month-old male mice) seven times over 2 weeks, and then the two groups of aged mice were either assessed by a series of behavioral paradigms to determine memory ability and endurance performance or assessed by multiple senescence biomarkers to determine senescent phenotypes. Young male mice (2 months) were simultaneously injected with PBS to serve as a control group. **a**, The escape latency of each group in the training phase of Morris water maze test (*n* = 6). Blue and red asterisks indicate statistically significant differences between PBS → Young versus PBS → Aged and between Young sEV → Aged versus PBS → Aged, respectively. **b**,**c**, Time spent in the target quadrant and the number of platform crossings by each group in the probe trial of Morris water maze test (*n* = 6). **d**, Freezing levels of each group in the contextual fear conditioning test (*n* = 6). **e**, Running time to exhaustion for each group in the treadmill running test (*n* = 10). **f**, Representative images of SA-β-gal staining in tissue sections. Scale bar, 100 μm. **g**, Western blot analysis of p21 and p16 protein levels in various tissues. Representative western blots are shown. **h**, Immunohistochemistry staining of Ki67 in the hippocampus sections. Representative immunohistochemistry staining images are shown. Scale bar, 100 μm. Ki-67-positive staining is identified by the presence of brown nuclear staining (green arrows) in cells. **i**–**j**, Relative ROS and AGE levels in various tissues (*n* = 4). **k**, Representative images of aldehyde fuchsine staining for lipofuscin in tissue sections. Aldehyde fuchsine-positive staining appears dark purple in the sections. Scale bar, 100 μm. Each experiment was independently repeated four times with similar results in **f** and **k**. Significance was determined using one-way ANOVA followed by Dunnett’s multiple comparison test in **a**–**e**, **i** and **j**. *P < 0.05, **P < 0.01 and ***P < 0.005. Source data

Because the effects of aging on the brain and skeletal muscle are particularly deleterious, leading to cognitive dysfunction and sarcopenia^20,21^, we employed a series of behavioral paradigms to investigate whether young sEV injection can rapidly improve cognitive performance and endurance capacity in aged mice. In the Morris water maze test to assess hippocampus-dependent learning and memory function, aged mice reached the hidden platform with longer latency, spent less time in the target quadrant and had a decreased frequency of platform crossings than young mice (Fig. 2a–c). The injection of young sEVs significantly improved the ability of aged mice to find the hidden platform and increased their time spent in the target quadrant, along with more platform crossings (Fig. 2a–c), indicating that spatial learning and memory performance are improved in aged mice after young sEV treatment. In the contextual fear conditioning test to evaluate hippocampus-dependent associative memory, although young and aged mice exhibited equivalent freezing responses in the baseline training process, aged mice showed significantly lower levels of freezing during the contextual memory task (Fig. 2d). After the injection of young sEVs, aged mice displayed significantly enhanced contextual fear memory (Fig. 2d), suggesting that young sEVs mitigate hippocampus-related memory deficits in aged mice. In an incremental treadmill test to assess endurance performance, young mice ran up to 32.34 ± 0.73 min until exhaustion, whereas aged mice achieved a shorter running time of 19.77 ± 2.76 min (Fig. 2e). Injection of young sEVs significantly prolonged the total treadmill running time to 29.19 ± 0.27 min for aged mice (a 47.6% increase in running time) (Fig. 2e). As the basal physiological values and behavioral activity of aged mice may vary greatly, we compared the behavioral changes of the same batch of aged mice before and after the seven injections of PBS or young sEVs (Supplementary Fig. 3a). No changes in behavioral performance were observed in the aged mice before and after the injection of PBS (Supplementary Fig. 3b–f). However, compared to their performance before sEV treatment, aged mice exhibited enhanced spatial memory of the hidden platform location in the Morris water maze test, had improved associative memory in the contextual fear conditioning test and were more resistant to exhaustion during treadmill running after injection of young sEVs (Supplementary Fig. 3b–f). Furthermore, we performed a head-to-head comparison of the effects of young sEVs versus aged sEVs on behavioral phenotypes in aged mice. Aged male mice were intravenously injected with equal amounts of young or aged sEVs seven times over 2 weeks (Supplementary Fig. 4a). PBS-treated aged mice and aged sEV-treated aged mice exhibited similar patterns in behavioral tests, whereas PBS-treated young mice and young-sEV-treated aged mice performed consistently better in these behavioral paradigms (Supplementary Fig. 4b–f). This result rules out the possibility that the sEV administration itself, regardless of the age of the donor mouse, can induce physiological responses and affect aging process. Overall, these behavioral paradigms indicate that young sEVs rapidly ameliorate the pre-existing functional decline in aged mice.

Given that young sEVs have demonstrated the capability to rejuvenate aged tissues at both physiological and behavioral levels, it raises the question of whether aged sEVs might exert completely opposite effects, potentially accelerating the aging process. To elucidate this, we reversed the protocol and intravenously injected young mice with 200 μl of PBS or aged sEVs seven times over 2 weeks (Supplementary Fig. 4g). Compared to PBS-treated young mice, aged sEV-treated young mice exhibited a higher escape latency and had less swimming time in the target quadrant of the Morris water maze test (Supplementary Fig. 4h–j). Likewise, aged sEV-treated young mice displayed impaired associative memory in the contextual fear conditioning test and were more susceptible to exhaustion in the treadmill running test (Supplementary Fig. 4k,l). These behavioral results suggest that aged sEVs induce a rapid decline in memory function and endurance capacity in young mice.

Previous studies showed that young blood reverses age-related impairments in cognitive function and synaptic plasticity in aged mice^22^. Particularly, systemic administration of young blood plasma (100 µl per intravenous injection, from 3-month-old male mice) into aged male mice (18 months) eight times over 3 weeks rejuvenates cognitive function in both contextual fear conditioning and radial arm water maze paradigms^22^. To test whether young sEVs mediate the rejuvenating effects of young plasma, we intravenously injected aged mice with 200 μl of PBS or young plasma seven times over 2 weeks (Supplementary Fig. 5a). Injection with young plasma alleviated the characteristic behavioral traits of aging to a similar extent as injection with young sEVs (Supplementary Fig. 5b–f). On the other hand, similar results to those using aged sEVs were observed in the experiment injecting aged plasma into young mice (Supplementary Fig. 5g–l). Thus, young/aged sEVs recapitulate most of the behavioral phenotypes conferred by young/aged plasma, indicating that the beneficial/detrimental effects of young/aged plasma observed in heterochronic parabiosis models and plasma infusion are mediated, at least in part, by their sEV component.

### Effects of young sEVs on senescent phenotypes

One of the most well-characterized contributors to aging is senescent cells^2^. We determined the alteration of senescence-associated phenotypes in aged mice after young sEV injection. Senescent cells usually switch on the expression of acidic β-galactosidase, which is known as senescence-associated β-galactosidase (SA-β-gal), and the presence of this active enzyme indicates the senescent state of cells^23^. Although the percentage of SA-β-gal-stained cells in the heart and muscle of aged mice is quite low, aged mice indeed exhibited an apparent increase in SA-β-gal activity in the liver, spleen, lung, kidney, hippocampus and testis compared to young mice (Fig. 2f). However, young sEV treatment, even when administered for a short time of 2 weeks, rapidly reduced the level of SA-β-gal staining in the liver, spleen, lung, kidney, hippocampus and testis (Fig. 2f). Another well-established marker of cellular senescence is high expression of cell cycle inhibitors p21 and p16, which renders the senescence growth arrest irreversible^24^. As expected, aged mice displayed increased p21 and p16 expression levels in various tissues (Supplementary Fig. 6a,b). However, short-term treatment with young sEVs significantly attenuated p21 and p16 expression in these tissues (Fig. 2g and Supplementary Fig. 6c). Furthermore, because senescent cells are known to be non-proliferative^25^, Ki67, an endogenous marker of cell proliferation, was specifically examined in the hippocampus region of young-sEV-treated aged mice. Compared to young mice, the percentage of Ki67-positive cells was significantly lower in the hippocampus of aged mice (Fig. 2h and Supplementary Fig. 6d). However, young sEV treatment rapidly alleviated this senescent phenotype in aged mice, as reflected by the emergence of more Ki67-positive proliferating cells in aged hippocampus (Fig. 2h and Supplementary Fig. 6d). Because senescent cells are also characterized by excess production of reactive oxygen species (ROS) due to the dysfunctional mitochondria^26^, we quantified ROS levels in young-sEV-treated aged mice. Intracellular ROS levels were substantially increased in a variety of tissues of aged mice, but young sEV treatment restored this marker to the normal levels detected in young mice (Fig. 2i). In addition, some additional morphological and molecular features of aging, including accumulation of advanced glycation end products (AGEs) and aggregation of lipofuscin, were evaluated in young-sEV-treated aged mice. AGEs, formed by a non-enzymatic reaction between the ketone or aldehyde groups of sugars and the amino groups of proteins, are accumulated as a sign of aging or degenerative disorder^27^. Although AGE content was significantly increased in various tissues of aged mice, young sEV treatment rapidly eliminated the accumulation of excessive AGEs in aged mice (Fig. 2j). Lipofuscin is a pigment that accumulates with age in the lysosomal compartment of post-mitotic cells^28^. Although the liver, heart, kidney and hippocampus of aged mice exhibited strong positive staining for lipofuscin, manifested as dark brown–black granules in the sections, aged mice that received a short-term injection of young sEVs clearly exhibited fewer perinuclear and cytoplasmic aggregates of lipofuscin in these tissues (Fig. 2k).

On the other hand, long-term injection of young sEVs was also efficient in rejuvenating the aged tissues and inhibiting cellular senescence. As shown by SA-β-gal staining, young-sEV-treated aged mice exhibited a substantial reduction in SA-β-gal activity in various tissues, especially in the spleen, kidney, hippocampus and testis, where very few SA-β-gal-positive cells were observed (Supplementary Fig. 7a). Consistently, long-term treatment with young sEVs resulted in a marked reduction in p21 and p16 expression in each aged tissue tested (Supplementary Fig. 7b,c).

### Young sEVs alter the proteomes of multiple aged tissues

**Fig. 3:**
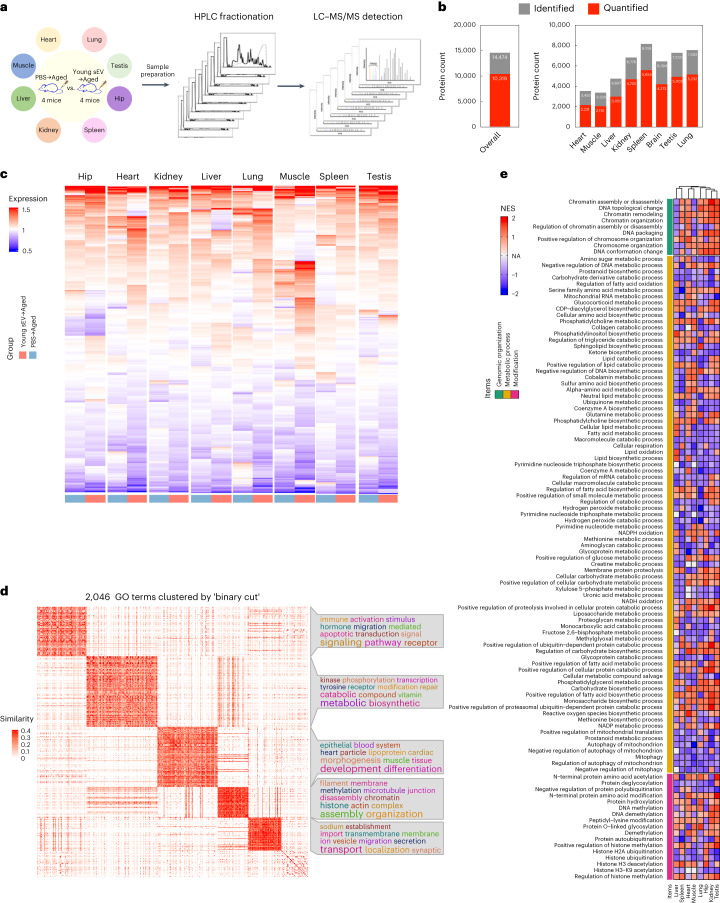
Young sEV treatment affects the proteomes of various aged tissues. **a**, Flow chart of the experimental design. Aged male mice (21 months) were intravenously injected with 200 μl of PBS or young sEVs (from 2-month-old male mice) seven times over 2 weeks, and then the two groups of aged mice were euthanized to extract total protein from eight tissues (heart, liver, spleen, lung, kidney, hippocampus, muscle and testis). Approximately 100 μg of protein isolated from each tissue was used for iTRAQ experiments. **b**, The numbers of proteins identified and quantified overall and in each tissue are shown. **c**, Heatmap showing the distribution of proteins in each aged tissue from groups treated with young sEVs and PBS. **d**, Heatmap of the similarities of 2,046 GO terms exported from the leading-edge analysis of GSEA was generated using the ‘binary cut’ algorithm with the R package simplifyEnrichment. The ‘binary cut’ algorithm automatically extracts the biological descriptions for GO terms, clusters the similarity matrices of functional terms into groups and visualizes the biological functions of each group as word clouds in a similarity heatmap. The word cloud annotation visualizes the summaries of biological functions in each GO cluster. **e**, Heatmap of the differentially expressed GO terms in each aged tissue after treatment with young sEVs. The GO terms with a similar enrichment condition (>6 of 8 tissues had enrichment scores greater or less than 0) in clusters 2 and 4 were finally chosen for heatmap rendering. HPLC, high-performance liquid chromatography. NES, normalized enrichment score; NA, not apply.

To reveal the mechanism of young sEV treatment at the proteome level, we collected eight tissues from PBS-injected and young sEV-injected aged mice and performed isobaric tags for relative and absolute quantification (iTRAQ)-based quantitative proteomic analyses for individual tissues (Fig. 3a). A total of 14,474 proteins were identified, and 10,319 proteins were quantified (Fig. 3b). A heatmap was generated with the R package clusterProfiler to obtain an overview of the proteome-level changes induced by young sEVs (Fig. 3c). Although the expression levels of most proteins showed no clear separation between groups treated with PBS and young sEVs, some proteins showed substantial upregulation or downregulation after treatment. Accordingly, uniform manifold approximation and projection (UMAP) annotation of the iTRAQ quantitative proteomic data showed a clear separation between the spleen, hippocampus and lung samples from PBS-injected and young-sEV-injected aged mice (Supplementary Fig. 8).

Subsequently, we employed gene set enrichment analysis (GSEA) software to conduct Gene Ontology (GO) enrichment analysis, aiming to further elucidate the effects of young-sEV-induced proteome changes on biological processes. After GSEA, leading-edge analysis was applied to identify the enriched terms or gene sets considered of high biological interest in all eight tissues. Then, the 2,046 GO terms exported from leading-edge analysis were clustered with the ‘binary cut’ algorithm using the R package simplifyEnrichment. Five GO term clusters were enriched, and the word cloud nicely highlighted the common biological functions involved in each cluster (Fig. 3d). (1) Cluster 1 was more specifically enriched in ‘signal transduction and signaling pathway’ (typical terms: regulation of intracellular signal transduction, regulation of apoptotic signaling pathway and androgen receptor signaling pathway). (2) Cluster 2 was strongly linked to ‘metabolic process’ (typical terms: mitochondrial RNA metabolic process, cellular respiration, NADH oxidation and lipid biosynthetic process) and ‘protein/DNA modification’ (typical terms: DNA methylation, DNA demethylation, protein hydroxylation and protein deglycosylation). (3) Cluster 3 was tightly linked to ‘tissue development, morphogenesis and differentiation’ (typical terms: muscle tissue development, platelet morphogenesis and leukocyte differentiation). (4) Cluster 4 was specifically related to ‘organization and assembly’, including ‘chromosome organization and modification’ (typical terms: chromosome organization, chromatin assembly or disassembly, chromatin remodeling, histone ubiquitination, histone H3 deacetylation and regulation of histone methylation) and ‘organelle assembly and disassembly’ (typical terms: nuclear membrane organization, organelle disassembly, protein–lipid complex assembly, mitophagy, proteasome assembly and mitotic spindle assembly). (5) Cluster 5 was tightly linked to ‘transport, localization and secretion’ (typical terms: growth hormone secretion, synaptic vesicle exocytosis, protein localization to Golgi apparatus and regulation of intracellular lipid transport). Among these five clusters, deregulation of the GO terms in clusters 2 and 4 was closely related to three typical features of aging, including mitochondrial dysfunction, epigenetic alterations and genomic instability. Furthermore, the clustered GO terms closely related to the hallmark of aging in clusters 2 and 4 were integrated and visualized as a heatmap (Fig. 3e), which revealed that young sEV treatment resulted in similar trends for alterations in multiple GO terms among different tissues. Overall, these results reflect that young sEVs may reverse age-related degenerative changes through multiple mechanisms and exert rejuvenating effects in a comprehensive manner.

### Young sEVs counteract mitochondrial deficiency in vivo

**Fig. 4:**
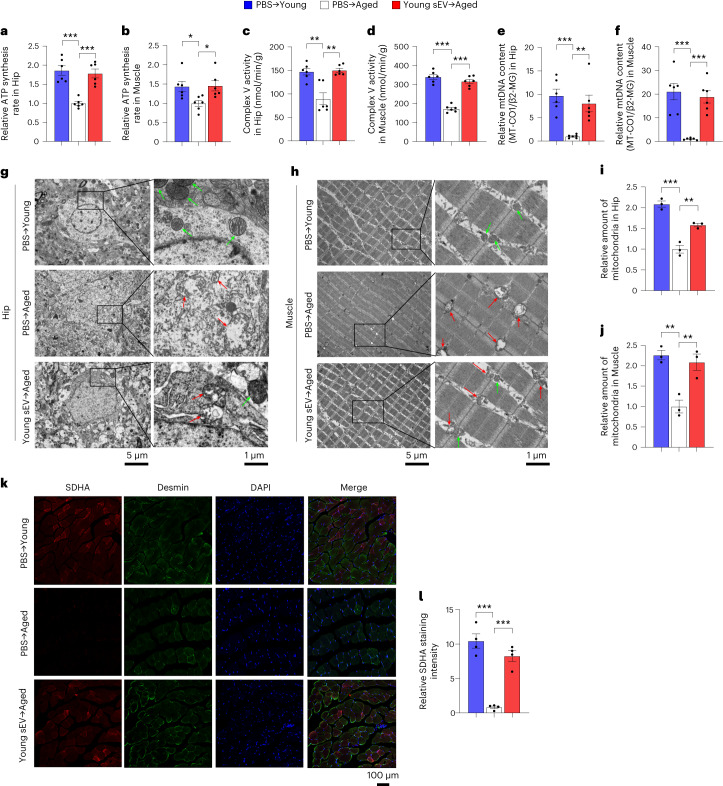
Young sEV injection counteracts mitochondrial deficiency and improves metabolic health in aged mice. Aged male mice (21 months) were intravenously injected with 200 μl of PBS or young sEVs (from 2-month-old male mice) seven times over 2 weeks, and then the two groups of aged mice were subjected to assessments of mitochondrial functional parameters and metabolic phenotypes. Young male mice (2 months) were simultaneously injected with PBS to serve as a control group. **a**,**b**, ATP synthesis rates in the hippocampus and muscle of each group (*n* = 6). **c**,**d**, Mitochondrial complex V activity in the hippocampus and muscle of each group (*n* = 6). **e**,**f**, Relative mtDNA content in the hippocampus and muscle of each group (*n* = 6). MT-CO1, normalized to β2-MG, was used to measure mtDNA copy number. **g**,**h**, Representative TEM images showing the structure and density of mitochondria in the hippocampus and muscle of each group. Normal mitochondria are round or oval shaped and contain well-defined cristae, whereas aged mitochondria become swollen, vacuolated and even broken, with cracked mitochondrial cristae. The green arrow indicates morphologically normal mitochondria, and the red arrow indicates morphologically damaged mitochondria. Scale bars, 5 µm in the left panel and 1 µm in the right panel. **i**,**j**, Quantification of the numbers of mitochondria in the sections (at low magnification) of hippocampus and muscle (*n* = 3). **k**,**l**, Immunofluorescence staining of SDHA (red), Desmin (green) and DAPI (blue) in the muscle fibers of each group. Representative images (scale bar, 100 μm) and quantification results (*n* = 4) are shown. Significance was determined using one-way ANOVA followed by Dunnett’s multiple comparison test in **a**–**f**, **i**, **j** and **l**. *P < 0.05, **P < 0.01 and ***P < 0.005. Source data

Mitochondria are ubiquitous intracellular organelles whose primary role is to supply cells with adenosine triphosphate (ATP)^29^. Despite their central role in cell metabolism, mitochondria are the most frequently damaged organelles during aging, which deteriorate with age, lose respiratory activity to produce ATP and accumulate damage to mitochondrial DNA (mtDNA), eventually leading to metabolic abnormalities and functional decline in various tissues^30^. Because the GO term cluster of ‘metabolic process’ was identified as a cardinal feature associated with proteome alterations in young-sEV-treated aged mice, we examined the alterations in mitochondrial functions and metabolic phenotypes in these mice. Compared to young mice, aged mice exhibited a significantly lower ATP synthesis rate and respiratory complex V activity in the hippocampus and muscle (Fig. 4a–d). Young sEV treatment led to a significant increase in ATP production and complex V activity in the hippocampus and muscle of aged mice (Fig. 4a–d). In addition, mtDNA copy number, a classical biomarker that reflects the abundance of mitochondria within a cell^31^, was assessed. Aged mice displayed reduced mtDNA copy number in the hippocampus and muscle compared to young mice (Fig. 4e,f and Supplementary Fig. 9a,b). In contrast, treatment with young sEVs reversed the reduction in mtDNA copy number in the hippocampus and muscle of aged mice (Fig. 4e,f and Supplementary Fig. 9a,b). Besides hippocampus and muscle, an apparent decline in mtDNA content was also observed in the heart, liver, spleen, lung, kidney and testis of aged mice, and young sEVs could also mitigate the loss of mtDNA content in these tissues after a short-term injection (Supplementary Fig. 9c–h). Conversely, aged sEVs did not induce any alteration of cellular ATP production, respiratory complex V activity and mtDNA content in hippocampus and muscle of aged mice, whereas young sEVs did (Supplementary Fig. 10a–f), which further confirmed the beneficial effects of young, but not aged, sEVs on mitochondrial energy metabolism.

During aging, damaged mitochondria with absent or disorganized cristae accumulate within cells^32^. To evaluate the effects of young sEVs on mitochondrial mass and morphology, we visualized the mitochondria at the ultrastructural level by TEM. The hippocampus and muscle of aged mice had fewer mitochondria than those of young mice, whereas more mitochondria were observed in the hippocampus and muscle of aged mice treated with young sEVs (Fig. 4g–j). Particularly, whereas the ultrastructure of the mitochondria present in the hippocampus and muscle of aged mice was very unhealthy, as manifested by many swollen or broken mitochondria, the percentage of abnormal mitochondria decreased significantly after young sEV treatment (Fig. 4g,h). Moreover, mitochondrial density and morphology were determined in the heart, liver, spleen, lung and kidney of young-sEV-treated aged mice by TEM. The proportion of mitochondria was significantly increased and the rate of swollen mitochondria and broken mitochondrial matrix was decreased in these tissues of aged mice after treatment with young sEVs (Supplementary Fig. 11). In addition, we performed immunofluorescence staining with antibodies against succinate dehydrogenase complex subunit A (SDHA, a major catalytic subunit of complex II of the mitochondrial respiratory chain, which served as a sensitive marker for mitochondria) and Desmin (a myogenic marker) to visualize mitochondrial distribution in muscle fibers^33^. Whereas the intensity of SDHA staining was significantly reduced in the muscle of aged mice, young-sEV-treated aged mice showed a significant increase in SDHA staining intensity in the muscle (Fig. 4k,l), indicating a recovery of mitochondrial content after treatment. Overall, these results suggest that young sEVs may increase mitochondrial mass and maintain mitochondrial function to increase ATP supply, thereby compensating for mitochondrial deficiency in aged tissues.

Conversely, when aged plasma sEVs were injected into young mice, they caused a significant reduction of cellular ATP production, respiratory complex V activity and mtDNA content in hippocampus and muscle of young mice (Supplementary Fig. 10g–l). In TEM images, both a decrease in mitochondria density and damaged mitochondrial structure were observed in hippocampus and muscle of young mice after treatment with aged sEVs (Supplementary Fig. 10m–p). These results indicate that aged sEVs can accelerate mitochondrial deficit and energy deficiency in vivo.

### Young sEVs enhance respiratory capacity in vitro

To assess the rejuvenating effects of young sEVs on cellular energy metabolism, we established an ex vivo model by isolating sEVs from the plasma of young mice and incubating them with a neuroepithelial NE-4C cell line or a myogenic C2C12 cell line (Supplementary Fig. 12a). Young sEV treatment accelerated the ATP synthesis rate, increased the enzymatic activity of complex V and increased the mtDNA copy number in NE-4C and C2C12 cells (Supplementary Fig. 12b–g). Additionally, the oxygen consumption rate (OCR), an indicator of oxidative phosphorylation (OXPHOS) and mitochondrial respiration^34^, was examined. Young-sEV-treated cells exhibited a significant increase in the basal OCR, ATP-coupled OCR and maximal OCR (Supplementary Fig. 12h–k), reflecting an enhanced OXPHOS capacity and electron transport activity. In addition, young sEVs inhibited cell senescence and rejuvenated cell proliferation in both cell lines, as manifested by reduced p21 expression and increased 5-ethynyl-2′-deoxyuridine (EdU) incorporation (Supplementary Fig. 12l–q).

**Fig. 5:**
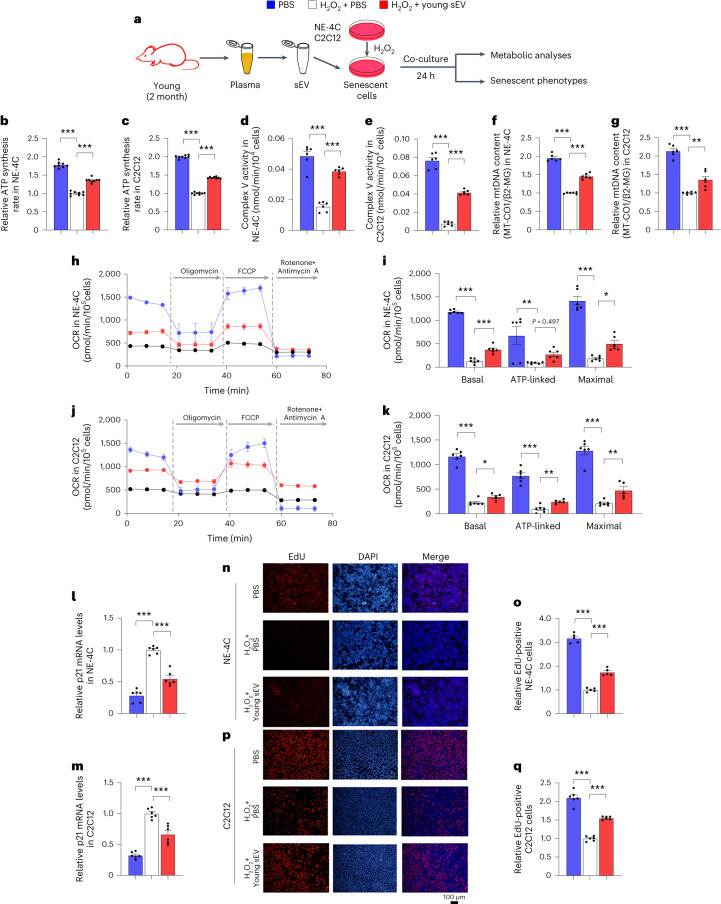
Young sEV treatment improves mitochondrial functions and attenuates H_2_O_2_-induced senescent phenotypes in cultured cells. **a**, Flow chart of the experimental design. NE-4C or C2C12 cells (1 × 10^6^ cells) were pretreated with H_2_O_2_ to induce cellular senescence and then incubated with 100 μl of PBS or young sEVs (from 2-month-old male mice) for 24 h, and then the cells were subjected to assessments of mitochondrial functional parameters and senescent phenotypes. Control cells were not treated with H_2_O_2_ but only with PBS. **b**,**c**, ATP synthesis rates in H_2_O_2_-induced NE-4C and C2C12 cells (*n* = 8). **d**,**e**, Mitochondrial complex V activity in H_2_O_2_-induced NE-4C and C2C12 cells (*n* = 6). **f**,**g**, Relative mtDNA content (MT-CO1/β2-MG) in H_2_O_2_-induced NE-4C and C2C12 cells (*n* = 6). **h**–**k**, Measurement of OCR in H_2_O_2_-induced NE-4C and C2C12 cells. After measurement of basal OCR, oligomycin, FCCP and rotenone+antimycin A were sequentially added, and the alterations in OCR were recorded and normalized to cell number. Quantification of the basal OCR, ATP-coupled OCR and maximal OCR is shown (*n* = 6). **l**,**m**, Quantitative RT–PCR analysis of p21 mRNA levels in H_2_O_2_-induced NE-4C and C2C12 cells (*n* = 6). **n**–**q**, EdU incorporation assay showing the proportion of proliferating cells in H_2_O_2_-induced NE-4C and C2C12 cells. Representative images (scale bar, 100 µm) and quantitative analysis of the percentage of EdU-positive cells (*n* = 5) are shown. Significance was determined using one-way ANOVA followed by Dunnett’s multiple comparison test in **b**–**g**, **i**, **k**–**m**, **o** and **q**. *P < 0.05, **P < 0.01 and ***P < 0.005. Source data

To ensure that young sEVs can rescue the dysfunction of aged mitochondria, H_2_O_2_ was used to induce cell senescence in NE-4C and C2C12 cells (Fig. 5a). Induction of cell senescence by H_2_O_2_ markedly reduced ATP synthesis rate, complex V activity and mtDNA copy number in NE-4C and C2C12 cells (Fig. 5b–g). Exposure to H_2_O_2_ also caused severe respiratory chain deficiency in cells, as indicated by the disruption of mitochondrial respiration during OCR measurements (Fig. 5h–k). However, treatment with young sEVs resulted in increased ATP production and mtDNA content in H_2_O_2_-induced cells (Fig. 5b–g). Likewise, treatment with young sEVs protected against H_2_O_2_-induced mitochondrial respiratory dysfunction in cells, as evidenced by the improved mitochondrial respiration tested by OCR measurements (Fig. 5h–k). In addition, whereas H_2_O_2_ enhanced p21 expression and reduced EdU incorporation in NE-4C and C2C12 cells, treatment with young sEVs suppressed cell senescence and rejuvenated cell proliferation ability in H_2_O_2_-induced cells (Fig. 5l–q). Overall, these results indicate that young sEVs can affect the key regulatory nodes of mitochondrial biogenesis and function to prevent mitochondrial dysfunction in senescent cells.

### Young human sEVs ameliorate age-related impairments in mice

Because the biological activity of sEVs exhibits little to no species specificity^35,36^, we investigated whether young human sEVs could ameliorate age-related impairments in mice. To this end, we purified human sEVs from the plasma of young male donors and intravenously injected them into aged mice at the same dose and frequency as mouse sEVs (Supplementary Fig. 13a). Behavioral tests revealed that young human sEVs efficiently ameliorated cognitive deficits and improved endurance capacity in aged mice (Supplementary Fig. 13b–f). Young human sEVs also significantly accelerated ATP production, stimulated complex V activity and increased the mtDNA content in the hippocampus and muscle of aged mice (Supplementary Fig. 13g–i). In particular, the loss of mitochondrial mass and structural integrity in aged hippocampal and muscle cells was substantially rescued by young human sEVs, as shown in TEM images of the ultrastructure of mitochondria (Supplementary Fig. 13j–m). Likewise, mitochondrial activity in muscles, as determined using succinic dehydrogenase (SDH) staining, was markedly recovered by young human sEVs (Supplementary Fig. 13n,o). Moreover, young human sEVs increased ATP production, complex V activity and mitochondrial content (Supplementary Fig. 14a–g); enhanced basal, ATP-coupled and maximal respiration during OCR measurements (Supplementary Fig. 14h–k); and caused a decrease in p21 levels and an increase in cell proliferation in NE-4C and C2C12 cells (Supplementary Fig. 14l–q). Overall, these results reveal that young human sEVs can rescue the dysfunction of aged mouse tissues and ameliorate age-related deficiencies in mitochondria in a similar manner to that of young mouse sEVs.

### Specific microRNAs are altered in plasma sEVs along with age

**Fig. 6:**
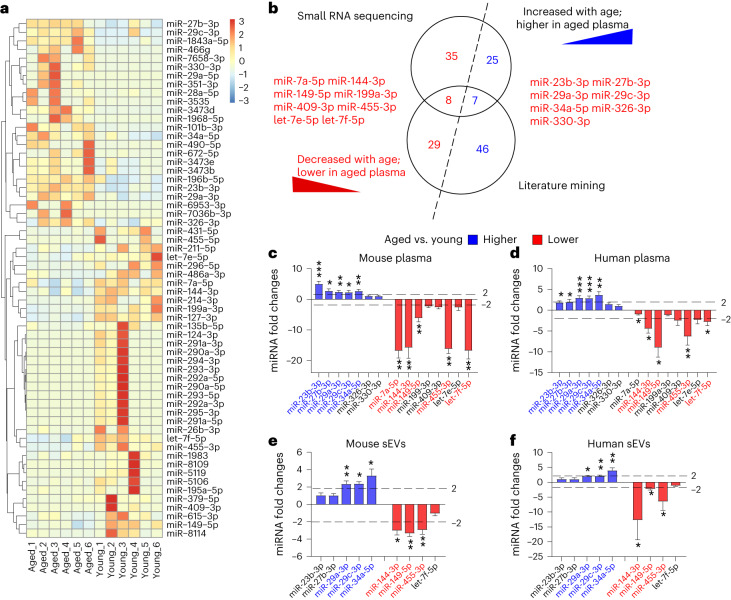
Alteration in the miRNA contents in plasma and sEVs during the aging process. **a**, Heatmap illustrating the differentially expressed plasma miRNAs (mean reads > 50, |fold change| > 2 and *P* < 0.05) in aged mouse plasma versus young mouse plasma (*n* = 6). **b**, Venn diagram showing the overlap of age-related circulating miRNAs that are identified by small RNA sequencing and literature mining. **c**,**d**, Fold changes of miRNAs in aged mouse plasma relative to young mouse plasma (*n* = 12) and in aged human plasma relative to young human plasma (*n* = 20). The blue box represents high relative expression level, and the red box represents low relative expression level. The names of miRNAs are marked with blue and red if they were significantly higher or lower in aged plasma versus young plasma (|fold change| > 2 and *P* < 0.05). **e**,**f**, Fold changes of miRNAs in aged mouse plasma sEVs relative to young mouse plasma sEVs (*n* = 4) and in aged human plasma sEVs relative to young human plasma sEVs (*n* = 6). The blue box represents high relative expression level, and the red box represents low relative expression level. The names of miRNAs are marked with blue and red if they were significantly higher or lower in aged plasma sEVs versus young plasma sEVs (|fold change| > 2 and *P* < 0.05). Significance was determined using two-sided Student’s *t*-test in **c**–**f**. *P < 0.05, **P < 0.01 and ***P < 0.005. Source data

Given the above-observed phenotypes, it remains unclear which component is responsible for the rejuvenating effects of young plasma sEVs. The inherent structure of sEVs enables the efficient transfer of informative molecules between cells, including proteins, lipids, DNAs and RNAs^37^. To date, a large number of studies have been directed at understanding the RNA components of sEVs, especially microRNAs (miRNAs)^38^. Indeed, the miRNA cargoes encapsulated within sEVs can be transferred to recipient cells, leading to important functional effects on target genes and cellular pathways, thereby regulating a previously unacknowledged mechanism of intercellular communication^39^. We hypothesized that some miRNAs in plasma sEVs may be the rejuvenation factor of young blood. To prove this, we first examined the miRNA profiles in aged and young mouse plasma by small RNA deep sequencing. According to the criteria of mean reads > 50, |fold change| > 2 and *P* < 0.05, 75 miRNAs were significantly differentially expressed (32 higher and 43 lower) in aged plasma compared to young plasma (Supplementary Table 2). Hierarchical clustering analysis clearly separated the aged plasma samples from young plasma samples based on plasma miRNA profiles (Fig. 6a). Second, we conducted a comprehensive review of the current literature on age-related circulating miRNAs (in plasma and serum) and summarized their changing patterns throughout the aging process. A total of 90 miRNAs were identified as the signature characterizing the aging process, among which 53 were increased and 37 were decreased along with age (Supplementary Table 3). Third, we compared the result of small RNA sequencing with that of literature mining. Specifically, 15 miRNAs (seven higher and eight lower in aged plasma versus young plasma) were shared by the two analysis strategies (Fig. 6b). Quantitative analysis further confirmed that 10 and nine miRNAs were significantly differentially expressed (|fold change| > 2 and *P* < 0.05) in aged versus young mouse plasma and in aged versus young human plasma, respectively, and nine miRNAs were consistently altered between mouse and human plasma (Fig. 6c,d). To further evaluate the biological relevance of age-related circulating miRNAs, we measured the nine miRNAs in the plasma sEVs from aged and young mice and humans. According to the criteria of |fold change| > 2 and *P* < 0.05, the contents of miR-29a-3p, miR-29c-3p and miR-34a-5p were significantly higher in the plasma sEVs from aged mice and humans, whereas miR-144-3p, miR-149-5p and miR-455-3p contents were significantly higher in the plasma sEVs from young mice and humans (Fig. 6e,f). Thus, miR-144-3p, miR-149-5p and miR-455-3p were considered as the representative cargoes of sEVs at the young state, whereas miR-29a-3p, miR-29c-3p and miR-34a-5p were characteristic of sEVs at the aged state.

Furthermore, we examined whether the miRNA cargoes of young sEVs can be taken up by aged tissues after young sEV injection. We labeled young sEVs with PKH26, a red fluorescent dye specifically designed to label the lipid bilayers of sEVs, and then intravenously injected PKH26-labeled sEVs into aged mice and tracked the distribution of young sEVs in vivo. Whereas the free PKH26 dye injection did not result in fluorescence accumulation in the tissue sections of aged mice, red fluorescence was clearly observed in the hippocampus and muscle of aged mice after young sEV injection (Supplementary Fig. 15), indicating the potential transfer of young sEVs from the bloodstream to tissues such as the hippocampus and muscle. Notably, aged sEVs injected into aged mice should have been labeled to assess the presence of donor aged sEVs across various tissues, mirroring the approach used for donor young sEVs. This additional step would have facilitated an investigation into whether aging affects the tropism of circulating sEVs, revealing a limitation left unaddressed in this study.

Next, we examined the uptake of sEV miRNAs by aged tissues. After the injection of young mouse sEVs into aged mice, both the mature and precursor forms of miR-144-3p, miR-149-5p and miR-455-3p were measured in recipient mice. Mature miR-144-3p, miR-149-5p and miR-455-3p were significantly upregulated in the heart, liver, spleen, lung, kidney, hippocampus, muscle and testis of aged mice (Supplementary Fig. 16a). However, no alteration in pre-miR-144, pre-miR-149 and pre-miR-455 levels was observed in these tissues (Supplementary Fig. 16b), suggesting that the endogenous levels of miR-144-3p, miR-149-5p and miR-455-3p were not induced by the injected young sEVs. Therefore, the elevation of miR-144-3p, miR-149-5p and miR-455-3p in aged tissues is likely due to sEV-mediated delivery of exogenous miR-144-3p, miR-149-5p and miR-455-3p to these tissues.

### miRNAs in young sEVs induce PGC-1α in vitro and in vivo

Next, we predicted the target genes that may be regulated by the miRNAs in young and aged plasma sEVs. By literature mining, we identified the key targets shared by the representative miRNAs in aged and young plasma sEVs. As shown in Supplementary Fig. 17a, peroxisome proliferator-activated receptor (PPAR) γ coactivator α (PGC-1α), a master regulator of mitochondrial biogenesis and function^40^, is a common target negatively regulated by miR-29a-3p, miR-29c-3p and miR-34a-5p^41–43^. In contrast, because the downstream target genes of miR-144-3p, miR-149-5p and miR-455-3p, including β-amyloid precursor protein (APP), poly(ADP-ribose) polymerase-2 (PARP-2) and hypoxia-inducible factor 1-alpha inhibitor (HIF1an), exhibit inverse correlation with PGC‐1α^44–47^, miR-144-3p, miR-149-5p and miR-455-3p can be considered as indirect stimulators of PGC‐1α expression (Supplementary Fig. 17a). The putative binding sites are illustrated in Supplementary Fig. 17b. Both the sequences of miRNAs and the sequences of miRNA binding sites are highly conserved across species (Supplementary Fig. 17b,c).

**Fig. 7:**
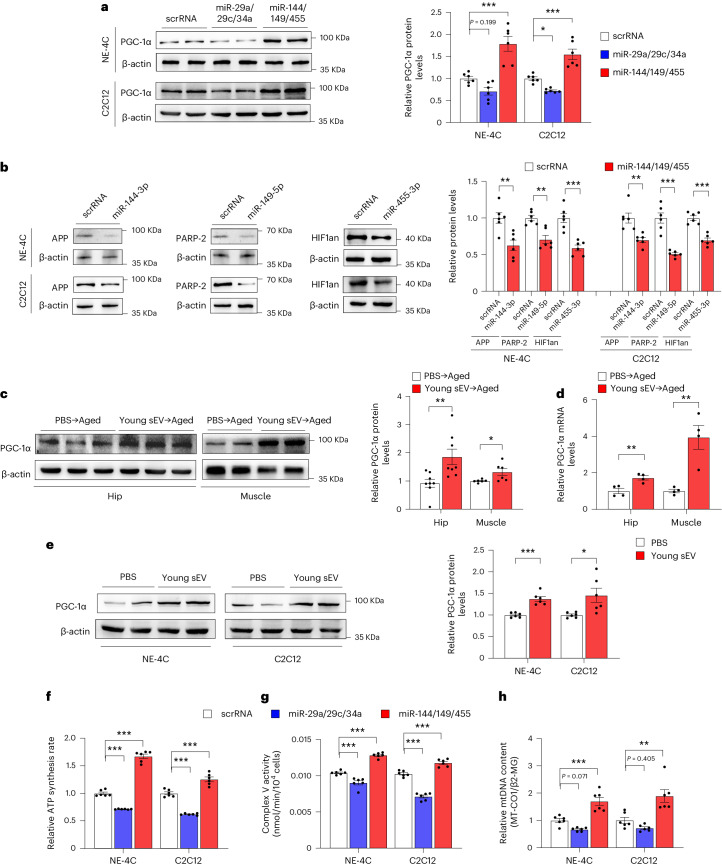
miRNAs in young and aged sEVs affect mitochondrial metabolism by regulating PGC-1α expression. **a**, Western blot analysis of PGC-1α protein levels in NE-4C and C2C12 cells transfected with the scrRNA, a combination of miR-29a-3p, miR-29c-3p and miR-34a-5p mimics (miR-29a/29c/34a) or a combination of miR-144-3p, miR-149-5p and miR-455-3p mimics (miR-144/149/455). Left, representative western blots. Right, densitometric analysis (*n* = 6). **b**, Western blot analysis of APP, PARP-2 and HIF1an protein levels in NE-4C and C2C12 cells transfected with the scrRNA or the corresponding miRNA mimic. Left, representative western blots. Right, densitometric analysis (*n* = 6). **c**, Western blot analysis of PGC-1α protein levels in the hippocampus and muscle of aged mice injected with 200 μl of PBS or young mouse sEVs seven times over 2 weeks. Left, representative western blots. Right, densitometric analysis (*n* = 8 for hippocampus; *n* = 6 for muscle). **d**, Quantitative RT–PCR analysis of PGC-1α mRNA levels in the hippocampus and muscle of aged mice injected with 200 μl of PBS or young mouse sEVs seven times over 2 weeks (*n* = 4). **e**, Western blot analysis of PGC-1α protein levels in NE-4C and C2C12 cells incubated with 100 μl of PBS or young mouse sEVs for 24 h. Left, representative western blots. Right, densitometric analysis (*n* = 6). **f**, ATP synthesis rates in NE-4C and C2C12 cells transfected with scrRNA, miR-29a/29c/34a or miR-144/149/455 (*n* = 6). **g**, Mitochondrial complex V activity in NE-4C and C2C12 cells transfected with scrRNA, miR-29a/29c/34a or miR-144/149/455 (*n* = 6). **h**, Relative mtDNA content (MT-CO1/β2-MG) in NE-4C and C2C12 cells transfected with scrRNA, miR-29a/29c/34a or miR-144/149/455 (*n* = 6). Significance was determined using two-sided Student’s *t*-test in **b**–**e** and one-way ANOVA followed by Dunnett’s multiple comparison test in **a** and **f**–**h**. *P < 0.05, **P < 0.01 and ***P < 0.005. Source data

As a key regulator of mitochondrial homeostasis, PGC-1α enhances mitochondrial biogenesis by coordinating the expression of mitochondrial proteins encoded by both nuclear and mitochondrial genomes, and it maintains the stability of the number and quality of mitochondria by regulating mitochondrial quality control and promoting mitochondrial regeneration^48^. The essential role of PGC-1α in mitochondrial biogenesis and OXPHOS motivates the hypothesis that the miRNA cargoes enriched in young plasma sEVs may exert a beneficial effect on mitochondrial energy metabolism by positively regulating PGC-1α, whereas the miRNAs enriched in aged plasma sEVs may exacerbate impaired mitochondrial biogenesis by negatively regulating PGC-1α. Consistent with this, PGC-1α expression was experimentally validated to be inhibited by miR-29a-3p, miR-29c-3p and miR-34a-5p mimics in NE-4C and C2C12 cells (Fig. 7a). In addition, miR-144-3p, miR-149-5p and miR-455-3p mimics directly suppressed APP, PARP-2 and HIF1an (Fig. 7b), which, in turn, led to increased PGC-1α expression in cells (Fig. 7a).

Subsequently, we investigated whether PGC-1α is associated with the rejuvenating effects of young sEVs in vitro and in vivo. Consistent with previous findings that PGC-1α is usually expressed at a high level in tissues with a great demand for ATP generation and decreases with age^48^, PGC-1α was indeed particularly vulnerable to aging compared to other metabolic genes and exhibited a significant reduction in the hippocampus and muscle of aged mice compared to young mice (Supplementary Fig. 18a). The injection of young sEVs derived from young mice into aged mice increased PGC-1α expression in the hippocampus and muscle (Fig. 7c,d), and young mouse sEVs also increased PGC-1α expression in NE-4C and C2C12 cells (Fig. 7e). Conversely, injection of aged sEVs into aged mice did not change PGC-1α expression in the hippocampus and muscle (Supplementary Fig. 18b). On the other hand, the stimulatory effect of young sEVs on PGC-1α expression in the hippocampus and muscle of aged mice and in NE-4C and C2C12 cells was validated by using the young sEVs derived from young human donors (Supplementary Fig. 18c–e). To determine whether the rejuvenating activities of young sEVs are exerted through their action on PGC-1α expression, a small interfering RNA (siRNA) targeting PGC-1α was transfected into NE-4C and C2C12 cells along with young sEVs. The siRNA-mediated PGC-1α silencing substantially diminished the beneficial effects of young sEVs on mitochondrial respiration (Supplementary Fig. 19). The findings indicate that the rejuvenating effects of young sEVs are at least partially mediated by modulation of PGC-1α expression.

To assess whether the miRNA cargo is indispensable to the rejuvenating activity of young sEVs, we pretreated young sEVs with Triton X-100 (to permeabilize the membranes of sEVs) and RNase, and then we incubated NE-4C and C2C12 cells with the resultant young sEVs. Previous studies showed that the membrane structure of sEVs is capable of protecting their RNA cargoes from degradation by RNase; hence, co-treatment with Triton X-100 and RNase rapidly degraded miRNA contents in sEVs^49^. Consistent with this, whereas intact young sEVs improved mitochondrial respiration in NE-4C and C2C12 cells, a significant reduction in mitochondrial OCR was detected in cells treated with the young sEVs derived from pre-incubation with Triton X-100 and RNase (Supplementary Fig. 20).

**Fig. 8:**
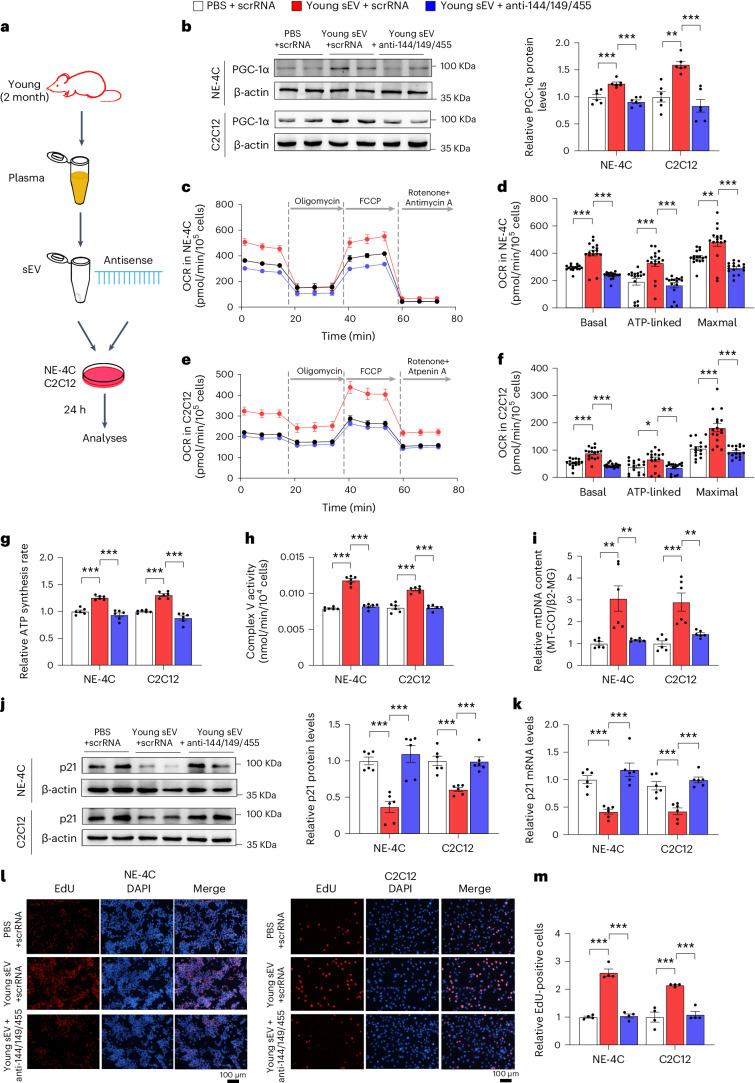
Antisense oligonucleotides of miR-144-3p, miR-149-5p and miR-455-3p block the beneficial effects of young sEVs on mitochondrial metabolism and cell senescence. **a**, Flow chart of the experimental design. NE-4C or C2C12 cells (1 × 10^6^ cells) were treated with PBS plus scrRNA, young sEVs plus scrRNA or young sEVs plus antisense oligonucleotides of miR-144-3p, miR-149-5p and miR-455-3p (anti-miR-144/149/455) for 24 h, and then the cells were subjected to assessments of mitochondrial functional parameters and senescent phenotypes. **b**, Western blot analysis of PGC-1α protein levels in NE-4C and C2C12 cells. Left, representative western blots. Right, densitometric analysis (*n* = 6). **c**–**f**, Measurement of OCR in NE-4C and C2C12 cells. After measurement of basal OCR, oligomycin, FCCP and rotenone+antimycin A were sequentially added, and the alterations in OCR were recorded and normalized to cell number. Quantification of the basal OCR, ATP-coupled OCR and maximal OCR is shown (*n* = 16). **g**, ATP synthesis rates in NE-4C and C2C12 cells (*n* = 6). **h**, Mitochondrial complex V activity in NE-4C and C2C12 cells (*n* = 6). **i**, Relative mtDNA content (MT-CO1/β2-MG) in NE-4C and C2C12 cells (*n* = 6). **j**, Western blot analysis of p21 protein levels in NE-4C and C2C12 cells. Left, representative western blots. Right, densitometric analysis (*n* = 6). **k**, Quantitative RT–PCR analysis of p21 mRNA levels in NE-4C and C2C12 cells (*n* = 6). **l**,**m**, EdU incorporation assay showing the proportion of proliferating cells in NE-4C and C2C12 cells. Representative images (scale bar, 100 µm) and quantitative analysis of the percentage of EdU-positive cells (*n* = 4) are shown. Significance was determined using one-way ANOVA followed by Dunnett’s multiple comparison test in **b**, **d**, **f**–**k** and **m**. *P < 0.05, **P < 0.01 and ***P < 0.005. Source data

Finally, we investigated whether the miRNA cargoes in young sEVs could improve mitochondrial metabolism by reinforcing PGC-1α expression. Induction of miR-144-3p, miR-149-5p and miR-455-3p in NE-4C and C2C12 cells by transfection with corresponding mimics led to increased respiratory rate, ATP production and mitochondrial mass (Fig. 7f–h). To specifically block the function of the internalized miR-144-3p, miR-149-5p and miR-455-3p, a combination of antisense oligonucleotides of miR-144-3p, miR-149-5p and miR-455-3p was transfected into NE-4C and C2C12 cells along with young plasma sEVs, and the cells were assessed for metabolic and senescent phenotypes in comparison with the cells treated solely with young sEVs (Fig. 8a). Whereas young sEVs exhibited considerable rejuvenating activity in stimulating PGC-1α expression, enhancing mitochondrial respiration and increasing ATP synthesis and mitochondrial content in NE-4C and C2C12 cells, antisense oligonucleotides completely reversed the effects of young sEVs on promoting PGC-1α expression and mitochondrial energy metabolism (Fig. 8b–i). Similarly, whereas young sEVs strongly prevented cell senescence and activated cell proliferation, as reflected by reduced p21 expression and increased EdU incorporation in cells, antisense oligonucleotides largely blocked these beneficial effects of young sEVs (Fig. 8j–m). Thus, miR-144-3p, miR-149-5p and miR-455-3p encapsulated in young plasma sEVs are the key rejuvenating miRNAs and have the potential to stimulate PGC-1α expression and improve mitochondrial energetic metabolism.

Conversely, we investigated whether the miRNA cargoes in aged sEVs could facilitate PGC-1α loss and drive age-related mitochondrial impairments. As expected, aged sEVs exhibited considerable inhibitory activity against PGC-1α expression in the hippocampus and muscle of young mice (Supplementary Fig. 18f). Likewise, direct transfection with miR-29a-3p, miR-29c-3p and miR-34a-5p mimics led to reduced PGC-1α expression in NE-4C and C2C12 cells (Fig. 7a), which was accompanied by loss of mitochondrial activity and mtDNA content in these cells (Fig. 7f–h). We then co-administrated antisense oligonucleotides of miR-29a-3p, miR-29c-3p and miR-34a-5p and aged sEVs to NE-4C and C2C12 cells. Aged sEVs strongly inhibited PGC-1α expression, caused impaired mitochondrial activity and reduced mtDNA content and promoted cellular senescence marker p21 in NE-4C and C2C12 cells; however, co-treatment with antisense oligonucleotides significantly rescued the detrimental effects of aged sEVs on mitochondrial biogenesis and metabolism, recovering PGC-1α expression, mitochondrial function and p21 level to a normal state (Supplementary Fig. 21), further proving that the pro-aging effects of aged sEVs are carried out, at least in part, through their miRNA cargoes. Therefore, miR-29a-3p, miR-29c-3p and miR-34a-5p in aged plasma sEVs are the key pro-aging miRNAs with an entirely opposite function to miR-144-3p, miR-149-5p and miR-455-3p encapsulated in young plasma sEVs.

## Discussion

Until very recently, aging was found to arise, in part, as a consequence of the alteration of EVs in peripheral blood. Indeed, blood-borne EVs are naturally positioned in multiple pathways affecting cell-to-cell signaling, and aberrant levels of EVs may alter intercellular communication and contribute to the characteristic features of aging^50^. Unlike soluble plasma factors, blood-borne EVs carry a payload of information that exerts a comprehensive and profound influence on systemic communication. In fact, previous studies provided substantial circumstantial evidence supporting the critical functions of EVs in aging. For example, EVs and their cargoes have been advocated as biomarkers for capturing the complexity of aging^51^. Additionally, induction of cellular senescence alters EV secretion and EV miRNA cargo in vivo^52^, and senescent cells release their EVs to the local tissue microenvironment or body fluids to exert an adverse effect on tissue homeostasis during aging^53^. Recently, direct in vivo evidence has emerged to provide preliminary insights into the contribution of EVs to aging. The most direct evidence is that EVs isolated from young mouse plasma have a clear pro-longevity role in aged mice. In detail, EV-mediated systemic delivery of extracellular nicotinamide phosphoribosyltransferase (eNAMPT) mitigates age-associated functional decline in specific tissues, delays the age-associated mortality rate and extends healthspan and lifespan in mice^54^. In the present study, we discovered a dominant rejuvenating effect of young sEVs on aged tissues. Several administrations of young sEVs led to a remarkable enhancement in the physical performance and physiological functions of aged mice, although aged blood was continuously produced to dilute, neutralize and remove the beneficial effects of young sEVs. Moreover, although the whole body improved rapidly within a few days after short-term injection of young sEVs, long-term injection of young sEVs even revitalized some properties and performance of aged tissues (hippocampus, muscle, heart, testis and bone) to an equivalent extent as in young mice. Consequently, young-sEV-treated aged mice exhibited a healthier appearance; a lower frailty index; enhanced cognitive ability, endurance performance and cardiac function; improved sperm quality and male fertility; increased energy expenditure; and decelerated bone loss at the physiological level. These results indicate that the rejuvenating effects of young EVs are pronounced and lasting.

The groundbreaking discoveries regarding the rejuvenating effects of young blood have inspired the concept of directly transfusing young blood to older adults suffering from age-related diseases. Recently, researchers have commenced the process of translating these findings from the laboratory to clinical practice. One pilot clinical study showed that a young plasma infusion into patients with Alzheimer’s disease was safe and produced some promising results, although no statistically significant improvements in cognition were detected^55^. Although blood products have been successfully used in clinical settings to treat various diseases over an extended period, their application in addressing age-related conditions presents distinct challenges and necessitates careful consideration before translation. These concerns relate to the potential risks and side effects inherent in blood infusion, particularly with respect to allergic reactions, hemolysis and transmission of infectious diseases. In addition, the limitations associated with identifying suitable young human donors for a potentially substantial population of aged patients cannot be underestimated. Hence, it is crucial to identify the precise rejuvenating factors inherent in young blood to simplify conventional blood products and develop a more effective component that can specifically target aging-related phenotypes. To this end, our findings suggest a possibility that uses sEVs as a medication for an effective anti-aging intervention. Indeed, sEVs have some innate advantages over other components of blood. First, as endogenous nanovesicles secreted from living cells, sEVs are biocompatible and non-cytotoxic and have low immunogenicity when administered in vivo. sEVs are also easy to store, have a long lifespan, possess high cargo loading capacity and can even access the central nervous system by crossing the blood–brain barrier. Second, sEVs offer a more refined alternative to whole blood. Their reduced complexity makes them safer and more accessible compared to the supply of whole blood. The side effects and potential risks associated with blood infusion may be considerably mitigated by the use of sEVs. Third, the volume of blood infusion is limited; however, because sEVs can be concentrated during purification, their rejuvenating effect can be amplified. However, despite the promising and encouraging results, there is a long journey ahead from experimental studies to the clinical application of young sEVs. Notably, the validation of the efficacy and safety of sEV injections in clinical trials is essential to establish them as a viable prophylactic or therapeutic approach for age-related diseases.

The discovery of the bioactivity of blood-borne sEVs is inspiring but raises the question of molecular mechanisms. Due to the pivotal role of sEVs in the transport of their cargoes, the search for active molecules within sEVs provides an opportunity to comprehensively delineate the effects of sEVs on the aging process. In the present study, we reported the activity of specific miRNAs in sEVs that partially recapitulate the revitalizing activity of young blood. In detail, miR-144-3p, miR-149-5p and miR-455-3p recapitulated the effect of young sEVs, reinforced PGC-1α expression and counteracted mitochondrial deficits, whereas miR-29a-3p, miR-29c-3p and miR-34a-5p attenuated PGC-1α expression and facilitated mitochondrial deficits. Thus, sEV miRNAs possibly play a dual role as a double-edged sword in the aging process: stimulating the cells and tissues to become more energetic in a young state while driving metabolic insufficiency to exacerbate functional decline in an aged state. The beneficial or detrimental aspects of sEV miRNAs will depend on their proportion in the blood circulation. The progressive accumulation of pro-aging miRNAs and loss of rejuvenating miRNAs during the aging process, which are manifested as increased vulnerability to mitochondrial deficits and increased accumulation of senescent cells, will eventually disrupt tissue homeostasis and aggravate energy deficits for physiological activity. However, sEVs are important for transferring not only miRNAs but also other cargoes, as the composition of sEV cargo is highly complex, consisting of hundreds to thousands of diverse proteins, lipids, DNAs and RNAs^56^. Although our study largely focused on the miRNA component of sEVs, we do not expect that miRNA is the sole active component in sEVs. Indeed, miRNA is only a class of small RNAs enriched in sEVs; small RNAs account for only a portion of total RNAs in sEVs; and RNA is only one type of component of sEVs. sEV miRNAs are unlikely to operate as an all-around factor in young blood; instead, more sEV components (other regulatory RNAs, mRNAs, DNAs, lipids, cytosolic proteins and membrane-bound proteins) likely function in a cooperative manner to regulate the aging process. Consistent with this, some previous studies focused on proteins enriched in young EVs and showed that the EV-enclosed eNAMPT and glutathione-S-transferase mu 2 (GSTM2) protein rejuvenated the regenerative capacity of aged tissues and declined with advanced age^54,57^. Likewise, it has been found that the impact of young EVs on muscle progenitor cell bioenergetics and myogenicity and skeletal muscle regeneration is dependent on the presence of abundant transcript levels of the pro-longevity protein α-Klotho in young, but not old, EVs^58^. Future studies should investigate the relative contributions of different components of blood-borne sEVs to the aging process, thereby providing a strong rationale for the therapeutic use of sEVs to treat age-related diseases. Nevertheless, the identification of miRNA profiles in sEVs derived from young and aged plasma represents an initial milestone in comprehending the molecular mechanisms underlying sEV-mediated aging. From the perspective of translation, elucidating the molecular mechanism governing the functional switch of miRNAs within sEVs and maintaining a stable pool of rejuvenating miRNAs in circulation may offer a therapeutic strategy for modulating the aging process and mitigating its impact on overall health and well-being.

Aging is associated with a reduction in ATP production through OXPHOS, primarily due to impaired mitochondrial function. In the present study, we provided compelling evidence that young plasma sEVs were internalized by aged tissues and cells, which increased PGC-1α expression, facilitated mitochondrial metabolism and preserved the high level of energy support for aged tissues. Induction of PGC-1α expression and activity by young sEVs is, therefore, expected to become a therapeutic strategy for circumventing age-related mitochondrial dysfunction. However, although this study largely focused on PGC-1α and mitochondrial metabolism, we do not expect that PGC-1α is the sole downstream target of the rejuvenating miRNAs in young sEVs. The involvement of other miRNA–target pairs must contribute to the repair of aged tissues and aging-related pathologies. For example, aging is also associated with a decline in autophagic capacity and an increase in oxidative stress^59^. By in-depth analysis of the proteomic data using the GO term GOBP_AGING (including GOBP_CELL AGING) in GSEA, several proteins related to autophagy dysfunction and antioxidant responses were found to be substantially altered in the hippocampus and muscle of young-sEV-treated aged mice (Supplementary Fig. 22). Future studies are needed to examine whether these altered proteins are downstream targets of the miRNAs in young sEVs.

Taken together, our findings reveal that young plasma sEVs counteract pre-existing aging at the molecular, mitochondrial, cellular and physiological levels. Because young sEVs are promising natural vehicles that stably transport rejuvenation factors without causing toxicity or immunogenicity, they provide a unique opportunity to serve as a versatile tool for rejuvenating aging tissues and improving lifelong health and well-being.

## Methods

### Animal model

Wild-type C57BL/6J mice were purchased from the Model Animal Research Center of Nanjing University. Male mice were exclusively used for all experiments conducted in this study. These mice were housed in a controlled environment with a 12-h light/dark cycle (illuminated from 7:00) and granted unrestricted access to both food and water. All experiments were approved by the Animal Ethical and Welfare Committee of Nanjing University. The approved animal ethical and welfare number is IACUC-2010001. All animal care and handling procedures were performed in accordance with National Institutes of Health Guidelines for the Care and Use of Laboratory Animals.

### Plasma and sEV isolation

Human blood was extracted from each donor by aseptic venipuncture and collected into a vacuum tube (~5 ml) containing EDTA as an anticoagulant. Mouse blood was collected by intracardiac puncture into an Eppendorf tube (~1.5 ml) containing trisodium citrate as an anticoagulant. Plasma was obtained by centrifuging whole blood at room temperature for 15 min at 1,600*g*. The purified plasma samples were immediately centrifuged to obtain the purified sEVs or stored at −80 °C.

Plasma sEVs were isolated by ultracentrifugation. In brief, the plasma was diluted with an equal volume of PBS and subjected to a series of centrifugation steps at different speeds and durations. Specifically, centrifugation was performed at 500*g* for 5 min at 4 °C, followed by 3,000*g* for 25 min at 4 °C, 12,000*g* for 60 min at 4 °C and, finally, 120,000*g* for 70 min at 4 °C (polycarbonate tubes, 355630, Beckman Coulter; MLA-55 rotor, Optima MAX-XP, Beckman Coulter). The sEV pellet was collected, and the remaining supernatant was further processed using the Total Exosome Isolation Kit (from plasma) (Invitrogen, 4484450) according to the manufacturer’s instructions. In brief, 0.2 volumes of Exosome Precipitation Reagent were added to the supernatant, which was then incubated at room temperature for 10 min. Subsequently, sEVs and the supernatant were separated via centrifugation at 10,000*g* for 30 min at 4 °C, with the resulting sEV pellets being collected and resuspended in PBS.

The yield of sEVs was assessed by quantifying the total protein content using the Bicinchoninic Acid (BCA) Kit (Thermo Fisher Scientific). Approximately 1,000 μg of total protein content of sEVs could be obtained from 1 ml of plasma. Because the recovery yield of sEVs from viscous body fluids (plasma and serum) using ultracentrifugation-based procedures has been estimated to range from 5% to 25%^60^, the genuine sEV content in plasma might be several folds higher than the measured value (1.00 μg of total protein per microliter). To simulate the natural concentration of sEVs in plasma, the volume of PBS solution used to dissolve sEVs was approximately 0.56 times the original volume of plasma, so the final concentration of sEVs in PBS was generally 1.80 μg of total protein per microliter. The sEV samples were promptly stored at 4 °C for short-term storage and at −20 °C for long-term storage.

The size and concentration of sEVs were analyzed using a NanoSight LM10 system, as previously described^61^. The morphology and size of sEVs were also examined using a Hitachi TEM system (Hitachi, HT7700), as previously described^61^. To analyze the purity and protein contents of sEVs, the sEVs supplemented with RIPA lysis buffer were subjected to western blotting for the classical sEV markers (CD63, ~50 kD; TSG101, ~47 kD; Alix, ~100 kD; and CD9, ~25 kD), along with the major plasma protein albumin (~70 kD) and the endoplasmic reticulum protein calnexin (~55 kD). To analyze the RNA contents of sEVs, total RNA was isolated from sEVs using TRIzol reagent, and the levels of sEV miRNA were quantified.

### Injection of sEVs

In long-term experiments, young sEVs were isolated from the plasma of 2-month-old male mice using ultracentrifugation and subsequently diluted in PBS to achieve a concentration of 1.80 μg of total protein per microliter. Aged male mice (20 months) were intravenously injected with 200 μl of PBS or young sEVs once a week. At different timepoints, changes of physiological activities and functions, including sperm quality and male fertility (22-month-old mice receiving eight injections); metabolic rate and energy expenditure (23-month-old mice receiving 12 injections); cardiac functions (23.5-month-old mice receiving 14 injections); bone microarchitecture (24-month-old mice receiving 16 injections); and brain volume alterations (24.5-month-old mice receiving 18 injections), were evaluated. As a control, a group of young male mice (2 months) receiving only PBS was simultaneously monitored.

In short-term experiments, sEVs were isolated from the plasma of 2-month-old or 20-month-old male mice using ultracentrifugation. These sEVs were then diluted in PBS to achieve a concentration of 1.80 μg of total protein per microliter. Aged male mice (21 months) were intravenously injected with 200 μl of PBS or young sEVs seven times over 2 weeks, and young male mice (2 months) were intravenously injected with 200 μl of PBS or aged sEVs seven times over 2 weeks. PBS was injected at the same volume (200 μl) and frequency (seven times over 2 weeks) as a control. Moreover, sEVs were purified from the plasma of young male donors (19–24 years) and intravenously injected into aged mice (21 months) at the same dose (1.80 μg of total protein per microliter, 200 μl) and frequency (seven times over 2 weeks) as mouse sEVs. Subsequently, these mice underwent a battery of behavioral tests to evaluate memory and endurance performance, or they were evaluated using multiple senescence biomarkers to assess senescent phenotypes.

### Lifespan

The aging mouse cohorts were accommodated in the animal core facility at Nanjing University and granted unrestricted access to a standard laboratory diet and water. Mice allocated for the survival study were exclusively designated for this purpose and were not subjected to any other biochemical, physiological or metabolic analyses. Aged mice received intravenous injections of an equal volume (200 μl) of either PBS or young sEVs once a week. These mice were carefully monitored daily until their natural death.

### Fertility assessment

Aged males injected with PBS or young sEVs were mated with 4-month-old female mice. Female mice were proven to be fertile after a minimum of one litter. To conduct a continuous mating study, male and female pairs were co-housed for a 30-d period. Each morning, the females were examined for the presence of copulation plugs, pregnancy and litter size. The presence of a vaginal plug was considered indicative of successful mating within the pairs. The number of offspring per litter was documented using a camera (Canon). To visualize implantation sites in the uterus, males and females were paired overnight. The presence of a vaginal plug the next morning signified successful mating, and the midpoint of that day was designated as 0.5 dpc. The embryo implantation was examined at 4.5 dpc in pregnant mice. Approximately 5 min after intravenously injecting 200 µl of 1% Evans Blue per mouse, the mice were euthanized, and pictures of the incised uterus were taken. The blue bands were recorded and counted as the implantation site.

### Quantification of testosterone levels by ELISA

Testosterone levels were quantified using ELISA kits (Thermo Fisher Scientific) following the manufacturer’s instructions. In brief, mouse plasma samples were collected in 1.5-ml centrifuge tubes containing trisodium citrate dihydrate, and tissue samples were homogenized in PBS (protease inhibitors were added to PBS) on ice. Then, the samples were competed with a fixed amount of target on the solid phase supporter, where the target sites were biotinylated detection antibodies specific to the target. Subsequently, the HRP-conjugated streptavidin–biotin complex (SABC) was added to each well of the microplate and incubated. The enzyme–substrate reaction was initiated by the addition of TMB (3,3′,5,5′-tetramethylbenzidine) substrate solution, and the resulting color change was measured at a wavelength of 450 nm after terminating the reaction with a sulfuric acid solution.

### Sperm motility

The caudal region of the epididymis from each mouse was placed in a centrifuge tube containing Hank’s medium. Subsequently, the caudal region was transferred to a fresh dish with Hank’s medium and punctured at multiple points along the longitudinal axis using a surgical dissection blade to release sperm into the medium. The dish was then incubated at 37 °C for 5 min to allow sperm dispersion. Sperm motility parameters were evaluated using a computer-assisted sperm analysis (CASA) system equipped with Sperm Vision HR software version 1.01 (Minitube, Ingersoll). Each sample was subjected to the analysis of 200 spermatozoa.

### Sperm DNA integrity

Sperm DNA fragmentation was assessed using the Sperm Nucleus DNA Integrity Kit (Huakang Biomed) following the manufacturer’s guidelines. In brief, mouse semen samples were extracted from the epididymis and mixed with fluidized agarose in a tube. Approximately 30 μl of this mixture was deposited onto a pretreated slide and immediately covered with a coverslip. After a 4-min incubation at 4 °C, the coverslip was removed, and the slide underwent acid denaturation for 7 min, followed by 20 min of lysis. Subsequently, the slide was rinsed for 3 min with distilled water and sequentially dehydrated in 70%, 90% and 100% ethanol baths for 2 min each. After Wright’s staining, spermatozoa were counted to assess sperm DNA fragmentation using a bright-field microscope (Nikon).

### Indirect calorimetry analyses

Continuous monitoring of O_2_ consumption, CO_2_ production, heat production and physical activity was conducted using a combined indirect calorimetry system (TSE phenoMaster, TSE Systems GmbH), as previously described^15^. The system was maintained at a constant environmental temperature of 22.0 ± 1.0 °C and followed a 12-h light/dark cycle (with lights off between 18:00 and 6:00). Individual housing in metabolic cages was provided for the mice, allowing unrestricted access to food and water.

Young and aged mice were acclimated to the metabolic cages for 48 h before data collection. All data were recorded during the next 48-h period in metabolic cages. Energy expenditure was determined by measuring heat production (kcal h^−1^ kg^−1^), O_2_ consumption (ml h^−1^ kg^−1^) and CO_2_ production (ml h^−1^ kg^−1^). Physical activity of each mouse was recorded over 48 h (counts per 48 h) using a multidimensional infrared light beam system. Bar graphs were generated based on the results obtained using the indirect calorimetry system, which mean light and dark phase results and all-time results.

### Echocardiography

Cardiac function was evaluated using transthoracic echocardiography employing a Visual Sonics Vevo 3100 instrument equipped with a 30-mHz linear transducer probe (Fujifilm), as previously described^62^. Young and aged mice were induced with 2.5% isoflurane delivered in 1 L min^−1^ of compressed air, and anesthesia was sustained through an operative circuit nose GeroScience cone (Harvard Apparatus). Body temperature was monitored and maintained at 37 °C using a heating platform. Respiratory and heart rates were continuously monitored through a four-lead limb electrocardiogram, with isoflurane adjusted in 1 L min^−1^ compressed air to maintain a heart rate higher than 350 beats per minute (bpm). The mouse’s chest was shaved, and a layer of acoustic coupling gel was applied to the thorax. Subsequently, two-dimensional parasternal short-axis (SA) M-mode views of the left ventricle were acquired following optimization of gain settings, slight angulation and transducer rotation to enable imaging of both epicardial and endocardial surfaces. Particular care was taken during the procedure to prevent excessive thoracic pressure that could induce bradycardia. Cine loop analysis of cardiac cycles between respirations was conducted using VevoLab software to mitigate respiratory artifacts. Measurements of LV Vol;d, LV Vol;s, EF, FS and LV mass were obtained from a minimum of four cardiac cycles. Two observers, who were blinded to each other’s results, conducted all echocardiographic measurements.

### Micro-CT scanning

In vivo micro-CT analysis was conducted to evaluate the microstructure of the proximal tibia and vertebrae in both young and aged mice. Anesthesia was induced using 2.5% isoflurane delivered in 1 L min^−1^ compressed air and maintained through an operative circuit nose cone provided by GeroScience. Respiratory and heart rates were continuously monitored using a four-lead limb electrocardiogram. Micro-CT images were acquired using a Hiscan XM Micro CT analyzer (Suzhou Hiscan Information Technology), following previously established protocols^63^. All scans were performed with an 80-kV tube voltage and a 100-A tube current, capturing images at a 25-µm resolution. A 0.5° rotation step was employed over a 360° angular range, with a 50-ms exposure per step. Image reconstruction was carried out using Hiscan Reconstruct software (version 3.0, Suzhou Hiscan Information Technology), and subsequent analysis of the original three-dimensional (3D) images of the proximal tibia and vertebrae was performed using Hiscan Analyzer software (version 3.0, Suzhou Hiscan Information Technology). From the CT scans, parameters including tTb.Th, Tb.Sp, Tb.N and BV/TV were calculated for each mouse. Two independent observers, who were blinded to each other’s results, conducted all measurements on the micro-CT images.

### MRI scanning

Anesthesia was induced in both young and aged mice using 2% isoflurane and maintained at 1% throughout the MRI experiments. An oxygen generator with a flow rate of 2 L min^−1^, consisting of 90% oxygen and 10% isoflurane, was used and remained unchanged throughout the experiment. The mice were positioned in a horizontal bore 9.4T BioSpec 94/20 USR animal scanner (Bruker, Billerica). The body temperature and respiration rate of young and aged mice were continuously maintained using a warming pad and monitored using an MRI-compatible monitoring system. After scanning, young and aged mice were again transferred to the recovery room. T2WI and DTI protocols were used to ensure that images covered the volume between the olfactory bulb and the anterior part of cerebellum (field of view: 2.6 cm × 2.6 cm; thickness: 0.3 mm; 0.0703 × 0.0703 × 0.6 mm voxels; and 30 slices with no interslice gaps). Regions of interest (ROIs) were manually traced for the whole brain, hippocampus and cortex using the professional 3D medical image segment software ITK-SNAP 3.8.0 package (University of Pennsylvania) and based on previously reported methods^64,65^. The segmentation and volume calculations of MRI images were performed independently by two observers who remained blinded to each other’s results.

### SA-β-gal staining

SA-β-gal staining was conducted using a commercial β-galactosidase staining detection kit (Solarbio)^66^. Cryosections were fixed with the supplied fixative solution for 15 min at room temperature. After fixation, the sections were immersed in a fresh β-gal staining solution in buffer and incubated at 37 °C overnight. Microscopic images of the stained sections were captured using a microscope (Nikon). The intensity of SA-β-gal staining in these images was quantified using ImageJ software (National Institutes of Health).

### Immunohistochemistry of Ki67

Immunohistochemistry staining for Ki67 in the hippocampal sections was performed following the established protocol^67^. Hematoxylin staining was used to visualize cellular nuclei, which manifest as a blue hue, and the detection of Ki-67-positive staining relied upon the presence of discernible brown staining.

### Quantification of AGE and ROS levels by ELISA

Quantification of AGE^27^ and ROS^26^ levels was performed using ELISA kits following the manufacturer’s instructions. In brief, samples were homogenized in cold PBS, and the total protein concentrations were determined using the BCA Kit (Thermo Fisher Scientific). The levels of AGEs were quantified using a commercially available ELISA kit (mouse AGE ELISA kit, FineTest Biotech) in accordance with the manufacturer’s protocol. This process involved the use of a multifunctional microplate reader (SpectraMax i3x, Molecular Devices). The content of ROS was measured using the same method and kit (mouse ROS ELISA kit, FineTest Biotech).

### Lipofuscin staining (aldehyde fuchsine method)

Lipofuscin staining was conducted using a commercially available lipofuscin staining detection kit (Solarbio) in accordance with the manufacturer’s instructions^68^. The liver, kidney, heart and hippocampus were fixed in 4% (v/v) paraformaldehyde for 1 h. After dehydration and cryosectioning, tissue sections were stained in aldehyde fuchsine dyeing solution overnight at 37 °C. After this incubation, microscopic images of the sections were acquired using a microscope (Nikon).

### Morris water maze test

The Morris water maze test was conducted in accordance with established protocols to evaluate spatial memory performance^69^. In brief, a circular water pool measuring 1.2 m in diameter and 50 cm in height was filled with water at a constant temperature of 24 °C. To facilitate accurate identification of the C57BL/6J mice, white food-grade stain was added to the pool. An imperceptible fixed platform, measuring 10 cm in diameter, was situated 1 cm below the water’s surface. The mice were trained to navigate and locate the concealed platform within the water pool. To achieve this, the mice were exercised to form a ‘spatial orientation map’ in the brain using different figure shapes and colors as visual stimuli from extra-maze cues along the sidewall of the swimming pool to accomplish this task.

During the spatial training phase (days 1–5), mice were given a 60-s time limit to locate the hidden platforms. The trial concluded automatically once a mouse successfully located the platform, climbed onto it within 60 s and remained on the platform for at least 5 s. Each day, the mice underwent four trials starting from different directions, with intervals of more than 20 min between trials. In case a mouse failed to find the platform within the allocated 60 s on the first day of training, gentle guidance was provided to lead it to the platform. The time taken by the mice to locate and ascend to the platform was documented as the escape latency, using a video camera for accuracy. After the spatial training phase, a probe trial was conducted on day 6 to evaluate memory consolidation. During this trial, the platform was removed from the pool, allowing the mice to swim freely for 60 s. The time spent by each mouse in the target quadrant, where the platform was originally positioned, as well as the number of times the mice crossed the location where the platform had been, were recorded by video and subsequently quantified.

During the acquisition training phase, aged mice consistently displayed prolonged escape latency and traversed longer paths in their quest to locate the concealed platform^70^. In contrast, young mice exhibited shorter escape latencies and covered shorter distances in comparison^70^. In the subsequent probe trial, young mice demonstrated a higher propensity to spend time in the target quadrant and made more frequent crossings over the original platform location, whereas their aged counterparts spent notably less time in the target quadrant and primarily navigated the tank without crossing over the platform’s initial position^70^. These observations collectively suggest compromised spatial learning and memory performance in the aged mice.

In this test, the variables were strictly controlled to ensure rigor and reproducibility of behavioral tests. The water temperature was kept constant, and appropriate lighting was provided. The experimental environment (for example, humidity, temperature, smell and sound) was kept constant in all behavioral sessions. The animals were consistently handled and tested by the same investigators throughout the entire duration of the Morris water maze tests. Moreover, the investigators conducting the behavioral experiments remained blinded to the group assignments. All experiments were conducted between 10:00 and 17:00.

### Contextual fear conditioning test

The contextual fear conditioning test was conducted following established protocols^71^. In this test, mice were trained to associate the fear conditioning chamber with an aversive stimulus (mild foot shock; unconditioned stimulus (US)) for the assessment of contextual fear conditioning. Additionally, they were conditioned to associate tone cues (conditioned stimulus (CS)) with mild foot shocks to evaluate cued fear conditioning. The mice’s conditioned fear behavior was assessed by measuring freezing behavior.

On the training day (day 1), mice were placed in a fear conditioning chamber and allowed to acclimate to the environment for 2 min. After this, they received a 30-s tone (70 dB, 2 kHz) paired with a 2-s foot shock (0.6 mA). The CS–US pairing was repeated twice with a 2-min intermission. On the testing day (day 2), mice were placed in the same fear conditioning chamber for 3 min without any tone or foot shock to assess contextual memory. Freezing time was recorded and analyzed for this purpose. After a 2-h period, the mice were transferred to a new chamber with different odor, floor texture, chamber wall texture and chamber shape. They were given 2 min to acclimate, followed by a 30-s tone (70 dB, 2 kHz), and the freezing behavior was recorded and analyzed for 2 min to assess cued memory. The intensity of the foot shock was set at a level that was not painful but generated an unpleasant feeling for the mice. Freezing behavior was quantified and analyzed using a video tracking system and software (Taimeng).

Young and aged mice typically displayed similar levels of baseline freezing behavior during the training phase. However, during contextual memory testing, aged mice consistently exhibited lower levels of freezing compared to young mice^72^. These observations suggest the presence of non-spatial deficits in learning and memory in aged mice.

### Exhaustive running test

A treadmill running test was used to measure the endurance exercise capacity and was performed as described previously^69^. In brief, acclimatization to the treadmill was carried out over a period of 2 d before the experimental phase. This involved a 10-min run at 10 m min^−1^, followed by a 2-min run at 20 m min^−1^.

On the day of the exhaustive exercise, the mice were subjected to a progressive running protocol on a fixed 10° slope. This protocol consisted of running at 10 m min^−1^ for 15 min, followed by 13 m min^−1^ for 3 min, 16 m min^−1^ for 3 min, 19 m min^−1^ for 3 min and 22 m min^−1^ for 3 min, after which they ran at 25 m min^−1^ until exhaustion. Forced running was induced by positioning an electric grid with a voltage of 35 V at the end of the treadmill. Exhaustion was defined as the mice remaining on the shock grid for a continuous period of 5 s. The time to exhaustion was recorded. All animals were exercised at the same time to minimize diurnal effects. The animals were consistently handled and tested by the same investigators throughout the entire duration of the exhaustive running tests. Moreover, the investigators conducting the behavioral experiments remained blinded to the group assignments. All experiments were conducted between 10:00 and 17:00.

### sEV labeling and tracking

sEVs were labeled using PKH26 fluorescence kits following the manufacturer’s guidelines. In brief, sEVs suspended in PBS were mixed with PKH26 dye. After ultracentrifugation at 120,000*g* for 70 min, the supernatant containing the unbound dye was discarded, and the pellet was resuspended in PBS. Labeled sEVs were then intravenously injected into wild-type C57BL/6J mice. On day 2 after administration, the mice were anesthetized and transcardially perfused with ice-cold 0.9% saline to eliminate any remaining blood-derived sEVs in tissues. Hippocampus and muscle were excised, and frozen sections were analyzed using fluorescence confocal microscopy to visualize the red fluorescent signals.

### Protein reduction, alkylation, digestion and iTRAQ labeling

Protein extraction, protein concentration measurement and iTRAQ labeling were performed as previously described^73^. In brief, 100 µg of protein isolated from eight different organs/tissues (including heart, liver, spleen, lung, kidney, hippocampus, muscle and testis) was used for iTRAQ experiments. After TCEP (AB SCIEX) reduction, MMTS (AB SCIEX) alkylation and trypsin (Promega) digestion, the resulting peptides from each organ/tissue were collected and subjected to iTRAQ labeling using an iTRAQ Reagent 8-Plex Multiplex Kit (AB SCIEX). In one individual iTRAQ experiment, four biological replicate samples from PBS-injected aged C57BL/6J male mice were labeled with iTRAQ tags 113, 114, 115 and 116, and four samples from young-sEV-injected aged mice were labeled with iTRAQ tags 117, 118, 119 and 121. For each iTRAQ experiment, all labeled peptides were finally separated into 16 samples, desalted and concentrated for liquid chromatography with tandem mass spectrometry (LC–MS/MS) analysis, as previously described^73^.

### LC–MS/MS and database searching

MS data were acquired using a TripleTOF 5600+ System (AB SCIEX), as previously described^73^. The original MS/MS data were submitted to ProteinPilot version 4.5 (AB SCIEX) for database searching by searching against *Mus musculus* sequences in the UniProt database concatenated with the reverse decoy database (4 March 2021, containing 55,366 sequences; http://www.UniProt.org/proteomes/UP000000589). The searching parameters were set as described in a previous study^73^. The MS proteomics data have been deposited to the ProteomeXchange Consortium^74^ via the PRIDE^75^ partner repository with project accession code PXD030350.

### Bioinformatics analysis

Intensive bioinformatics analyses were conducted with R, RStudio, GSEA and other requisite packages and online tools.

To get an overview of quantitative proteomics data from all eight groups, both the identified proteins and the quantified proteins were counted for each group, and UMAP analysis was performed using the R package ‘umap’. Moreover, a whole-proteome heatmap was also generated by the R package clusterProfiler^76^ to determine the homogeneity in four samples injected with PBS in all eight organs/tissues and four samples injected with young sEVs in the same iTRAQ experiment as a quality control of biological replicates.

For in-depth functional class scoring, GSEA (http://www.gsea-msigdb.org) was performed using the R package clusterProfiler^76^ and GSEA software^77^. The significantly upregulated or downregulated GO terms between four samples injected with PBS and four samples injected with young sEVs in all eight tissues or organs were retained for further analysis. The GO terms of GSEA were clustered with the ‘binary cut’ algorithm using the R package simplifyEnrichment to further analyze the GSEA results. In the process of ‘binary cut’ clustering, difference score, cluster numbers and other parameters were set following the R package documentation^78^. Heatmaps with word cloud annotations were generated to summarize the functions of each enriched GO cluster.

### Protein extraction and western blots

For cellular protein isolation, cells were rinsed with PBS (pH 7.4) and subsequently lysed in RIPA lysis buffer supplemented with PMSF (1:100, Beyotime Biotechnology) and a phosphatase inhibitor cocktail (1:100, Thermo Fisher Scientific) at 4 °C for 30 min. For tissue protein isolation, samples mixed with RIPA, PMSF and phosphatase inhibitor cocktail were homogenized using a grinder at 60 Hz for 60 s, followed by lysis at 4 °C for 30 min. Subsequently, cell lysates and tissue homogenates were centrifuged at 12,000*g* and 4 °C for 10 min. The resulting supernatant was collected, and protein concentrations were determined using the BCA Protein Assay Kit (Thermo Fisher Scientific).

Samples containing equivalent amounts of protein (40–80 μg) were loaded and separated on 10–12.5% SDS-PAGE gels and subsequently transferred to PVDF membranes (Millipore). These membranes were blocked for 1 h at room temperature with 5% non-fat dry milk in TBS containing 0.1% Tween 20. After blocking, the membranes were incubated overnight at 4 °C with the appropriate primary antibodies. After three washes with 1× TBST for 10 min each, secondary antibodies were incubated for 1 h at room temperature, followed by an additional three 10-min washes with 1× TBST. The membranes were treated with ECL substrate (Thermo Fisher Scientific) following the manufacturer’s instructions, and protein levels were visualized using the Tanon 5200 multi-detection system (Tanon). Data were quantified using ImageJ software, and the relative protein levels were normalized to the β-actin level. Additional information on primary antibodies is available in Supplementary Table 4.

### RNA isolation and quantitative RT–PCR assay

Total RNA was extracted from cell and tissue samples using TRIzol reagent (Invitrogen) following previously described methods^69^.

For mRNA expression analysis, cDNAs were synthesized from 1 μg of total RNA using the AMV reverse transcriptase (TaKaRa) and oligo dT primers (TaKaRa). The reverse transcription reaction was carried out at 16 °C for 5 min, 42 °C for 60 min and 85 °C for 5 min. Quantitative RT–PCR was performed on an Applied Biosystems 7300 Sequence Detection System using a SYBR Green PCR kit. The PCR reactions underwent initial denaturation at 95 °C for 10 min and were then amplified for 40 cycles, with denaturation at 95 °C for 15 s, annealing at 60 °C for 30 s and extension at 72 °C for 30 s. All reactions were run in triplicate.

For miRNA expression analysis, cDNAs were synthesized from 500 ng of total RNA using the miRNA 1st Strand cDNA Synthesis Kit (Vazyme) with the stem-loop method. The reverse transcription reaction was carried out at 16 °C for 30 min, 42 °C for 30 min and 85 °C for 5 min. Quantitative RT–PCR was performed on the Applied Biosystems 7300 Sequence Detection System using miRNA Universal SYBR qPCR Master Mix (Vazyme) in a 96-well optical plate. The thermal cycling conditions included an initial denaturation step at 95 °C for 10 min, followed by 40 cycles at 95 °C for 15 s and 60 °C for 1 min. All reactions were run in triplicate.

After completion of the reactions, the C_T_ values were determined using fixed threshold settings, and the mean C_T_ was calculated from triplicate PCR measurements. The 2^−ΔΔCT^ method was employed to determine the relative expression of RNAs, with β-actin mRNA serving as the internal control for quantification of mRNAs and miR-16 serving as the internal control for quantification of miRNAs. The primer sequences used are provided in Supplementary Table 5.

### Measurement of ATP

The rate of ATP synthesis was determined using an ATP Determination Kit (Biovision) following the manufacturer’s instructions. This assay employs luciferase to facilitate the production of light from ATP and luciferin, which is subsequently measured using a luminometer. In brief, samples were homogenized with a nuclear releasing buffer for 5 min at room temperature. After this, lysate was added to the ATP monitoring enzyme for the calculation of ATP synthesis within 1 min using a luminometer. Protein concentrations were determined using the BCA Protein Assay Kit (Thermo Fisher Scientific) and applied for data normalization.

### Measurement of relative mtDNA content

mtDNA copy number was quantified using quantitative PCR, following a previously described protocol^31^. In brief, total DNA was extracted from tissues and cells using a DNA extraction kit (Biomed), in accordance with the manufacturer’s instructions^79^. A total of 10 ng of DNA was used for quantitative PCR analysis. The relative mtDNA copy number was determined by measuring the amplification of mitochondrially encoded genes, including *cytochrome c oxidase I* (*MT-CO1*), *NADH dehydrogenase 1* (*MT-ND1*), *cytochrome c oxidase III* (*MT-CO3*) and the D-loop region. These measurements were normalized to *β2-microglobulin* (*β2-MG*). The primer sequences used in the mtDNA assay are provided in Supplementary Table 5.

### Measurement of mitochondrial complex V activity

The activity of mitochondrial complex V was assessed using a mitochondrial respiratory chain complex V activity detection kit (Yifeixue Bio Tech), in accordance with the manufacturer’s instructions. In brief, tissues or cells were homogenized or ground, and mitochondria were extracted. Sonication was performed on ice using an ultrasonic cell disruptor (Sonics) at 200 W for 3 s, repeated 30 times at 10-s intervals. After a 30-min enzymatic reaction and a 10-min chromogenic reaction, the absorbance was measured using a microplate reader (Molecular Devices).

### SDHA immunofluorescence staining and SDH staining

SDHA immunofluorescence staining and SDH staining were performed following previously established protocols with slight modifications^80^. Muscle tissues were fixed with 4% paraformaldehyde in PBS (pH = 7.4) for 48 h at 4 °C. The fixed tissues were then dehydrated using a 30% sucrose solution. Cryosectioning was performed using a cryomicrotome (Leica). The sections were subsequently incubated overnight with a mouse anti-SDHA primary antibody (1:500, Abcam, ab814715), followed by staining with fluorescent dye-conjugated secondary antibodies (Life Technologies) for 1 h at room temperature. Finally, the sections were imaged using a confocal microscope (Nikon).

For SDH staining, mouse muscle samples were immediately immersed in OCT solution and frozen at −80 °C. Frozen sections with a thickness of 7 μm were prepared using a cryostat (Leica). These sections were then incubated with SDH reaction reagent at 37 °C for 40 min. After the enzymatic reaction, the reaction was halted by a rapid immersion in ddH_2_O and subsequent air drying for 10 min in the absence of light. Capturing images of the sections was conducted using a microscope (Nikon). The staining intensity of SDH in the images was quantified using ImageJ software.

### TEM analysis of the mitochondrial ultrastructure

Hippocampal and muscle samples were trimmed to 2 mm^3^ and fixed in 2.5% glutaraldehyde overnight at 4 °C. Subsequently, the samples underwent treatment with 1% osmium tetroxide and dehydration through a gradient of ethanol solutions. After staining with 1% uranylacetate and dehydration in acetone, samples were sliced into ultra-thin sections (80 nm) and loaded on electron microscopy grids. Images were acquired using a Hitachi TEM system (Hitachi, HT7700) operating at an acceleration voltage of 80.0 kV. The number of mitochondria was calculated in three fields for each sample section at ×20,000 magnification. The ultrastructure of mitochondria was captured at ×70,000 magnification and was identified as damaged if samples had disrupted membranes, cristae depletion, matrix dissolution and vacuolization.

### Cell culture

The mouse embryonic neural stem cell line NE-4C and mouse myoblast cell line C2C12 were obtained from the Shanghai Institute of Cell Biology, Chinese Academy of Sciences. NE-4C cells were cultured in complete mouse neural stem cell medium (Procell) at 37 °C with 5% CO_2_. To facilitate NE-4C cell adhesion and growth, 10 μg ml^−1^ poly-l-lysine was applied to the flask for at least 2 h before cell passage and cultivation. Furthermore, NE-4C cells were passaged using 0.05% trypsin instead of 0.25% trypsin. C2C12 cells were maintained in high-glucose (4.5 g L^−1^) DMEM (Gibco) supplemented with 10% FBS (Gibco, 10099141C) and cultured at 37 °C with 5% CO_2_.

Upon reaching 70% confluence, NE-4C cells were subjected to neuronal differentiation by supplementing the culture medium with 10 μM retinoic acid. After a 6-d differentiation period, NE-4C cells were either co-cultured with sEVs or transfected with miRNA mimics and antisenses. Similarly, C2C12 cells were induced to undergo the myoblast–myotube transition by switching the growth medium to differentiation medium (DMEM supplemented with 2% horse serum) when they reached 90% confluence. After 1 d, C2C12 cells were either co-cultured with sEVs or transfected with miRNA mimics and antisenses.

### Induction of cell senescence with high dose of H_2_O_2_

Cell senescence was induced following a previously described protocol^81^. On the first day, cells were cultured in T175 flasks and assessed under an inverted microscope (Nikon) to ensure that they reached approximately 50% confluence. If the cells had reached the desired confluence, the complete growth media were replaced with pre-warmed basal media containing 1.0 mM H_2_O_2_. The cells were then incubated in a cell culture incubator at 37 °C for 1 h. Afterwards, the basal media were changed back to complete growth media, and the cells were further incubated at 37 °C for the next 23 h. The next day, the cells were examined under an inverted microscope to confirm their viability and health. Subsequently, the complete growth media were replaced with pre-warmed basal media containing 750 μM H_2_O_2_. The cells were once again incubated in a cell culture incubator at 37 °C for 1 h. After that, the basal media were changed back to complete growth media, and the cells were incubated at 37 °C for another 23 h. The aforementioned steps were repeated four times until day 6 to induce cell senescence.

### Co-culture with sEVs and transfection with synthetic RNAs

For the co-culture experiments with sEVs, sEVs (50 μg of total protein content) were incubated with 1 × 10^6^ NE-4C or C2C12 cells and cultured in a humidified atmosphere with 5% CO_2_ at 37 °C. After 24 h, total RNA and protein were extracted from the cells. For cell transfection experiments, 50 pmol of miRNA mimics, antisense miRNAs or corresponding scrambled RNA (scrRNA) (GenePharma) were transfected into NE-4C or C2C12 cells using Lipofectamine 2000 (Invitrogen) following the manufacturer’s instructions. Total RNA and protein were extracted 48 h after transfection. The sequences of the synthetic miRNA mimics and antisenses can be found in Supplementary Table 6.

### EdU incorporation assay

An EdU incorporation assay was conducted following the previously described protocol with slight modifications^82^. NE-4C or C2C12 cells were seeded in 48-well plates after co-culture with sEVs or transfection with miRNA mimics or antisenses. When cells reached 80% confluence, they were treated with EdU reagent (RiboBio, C10310-1) to assess cell proliferation according to the manufacturer’s instructions. Cell nuclei were stained using DAPI (Beyotime Biotechnology), and the stained cells were visualized using a BX51 fluorescence microscope (Olympus).

### Measurement of OCR

OCR was analyzed using an XF96 Analyzer (Seahorse Bioscience). In brief, 2 × 10^4^ cells per well were plated in Seahorse XF96 plates in 200 μl of DMEM supplemented with 1 mM sodium pyruvate, 2 mM glutamine and 10 mM glucose. The plates were incubated for 1 h at 37 °C in a humidified incubator without CO_2_. After assessing the OCR at baseline, the following compounds were sequentially added to the analyzer, and the change in OCR was measured: (1) oligomycin (1.5 μM), which blocks ATP synthase and reduces OCR, representing mitochondrial respiration associated with cellular ATP production (ATP-coupled OCR); (2) carbonyl cyanide 4-(trifluoromethoxy)phenylhydrazone (FCCP, 0.5 μM), a mitochondrial uncoupler that enables maximal rates of electron transport and measures the maximal respiratory capacity (maximal OCR); and (3) a combination of rotenone and antimycin A (0.5 μM), which blocks respiratory electron flux through complexes I and III and inhibits mitochondrial respiration. All OCR measurements were normalized to the cell number in each well.

### Small RNA deep sequencing

Small RNA deep sequencing was conducted by BGI (Shenzhen) using a TruSeq Small RNA Sample Prep Kit (Illumina) for the construction of small RNA libraries. After the quality validation of these libraries, raw data were generated using the Illumina HiSeq 4000 platform. The clean reads were obtained through data filtration. Sequences corresponding to mature miRNAs and their precursors were extracted from miRBase version 21. Subsequently, the clean reads were aligned to reference sequences and annotated using the Bowtie algorithm. Only candidates with no more than one mismatch and not exceeding two shifts were considered as miRNA matches. Statistical analysis was conducted using Student’s *t*-test, with statistical significance defined as uncorrected *P* < 0.05 and |fold change| > 2. The average expression threshold was set at more than 50 reads.

### Literature mining of age-related circulating miRNAs

Age-related circulating miRNAs were identified through a PubMed-based literature mining approach. In brief, we conducted searches using several keywords, such as ‘plasma miRNA AND aging’, ‘serum miRNA AND aging’, ‘plasma miRNA AND metabolism’ and ‘serum miRNA AND metabolism’, on PubMed, applying the ‘Original article’ filter. We extracted all miRNAs reported in the literature and consolidated them after removing duplicates. A total of 458 miRNAs were identified in the published studies and subjected to further manual classification analysis. miRNAs were included if the studies focused on mouse or human miRNAs, if miRNA levels were measured using RNA deep sequencing or quantitative RT–PCR and if miRNAs were examined in at least three published papers. miRNAs were excluded if contradictory reports existed regarding their alterations in aged plasma (serum) compared to young plasma (serum). Ultimately, we identified 90 age-related circulating miRNAs, comprising 53 that exhibited increased levels and 37 that showed decreased levels with advancing age.

### Statistics and reproducibility

Statistical analyses were conducted using GraphPad Prism version 8 (GraphPad Software) or the open-source statistical package R. Significance was determined using two-sided Student’s *t*-test or one-way ANOVA followed by Dunnett’s multiple comparison test. Significance was assumed at *P < 0.05, **P < 0.01 and ***P < 0.005. Details on the statistics used can be found in the figure legends. No animals or data points were omitted from the analysis for any particular reason. The data are presented as the means ± s.e.m. *n* represents the number of samples used in the experiments. All micrographs and blots were representative of three independent experiments.

### Reporting summary

Further information on research design is available in the Nature Portfolio Reporting Summary linked to this article.

### Supplementary information

Supplementary Figs. 1–22 and Supplementary Tables 1–6.

Reporting Summary

Supplementary Data Source Data for Supplementary Figs. 1–22

### Source data

Source Data Fig. 1 Unprocessed images and statistical source data.

Source Data Fig. 2 Unprocessed western blots, unprocessed images and statistical source data.

Source Data Fig. 4 Unprocessed images and statistical source data.

Source Data Fig. 5 Unprocessed images and statistical source data.

Source Data Fig. 6 Statistical source data.

Source Data Fig. 7 Unprocessed western blots and statistical source data.

Source Data Fig. 8 Unprocessed western blots, unprocessed images and statistical source data.

## Data Availability

Raw data of iTRAQ have been deposited in the ProteomeXchange Consortium under accession code PXD030350. Raw data of small RNA sequencing have been deposited in the Genome Sequence Archive in the National Genomics Data Center, China National Center for Bioinformation, under accession code CRA013558. Quantitative data that support the findings of this study are available within this article and its supplementary files. Other data or materials that support the findings of this study are readily available from the corresponding author upon reasonable request.
