# Supplementary material for: Small extracellular vesicles from young plasma reverse age-related functional declines by improving mitochondrial energy metabolism
Source: Nat Aging. 2024 Apr 16;4(6):814–38. doi: 10.1038/s43587-024-00612-4 (PMC11186790; doi:10.1038/s43587-024-00612-4)
Supplement: Supplementary file 1 — Supplementary Figs. 1–22 and Supplementary Tables 1–6. [file 43587_2024_612_MOESM1_ESM.pdf]

nature aging

Article https://doi.org/10.1038/s43587-024-00612-4

Small extracellular vesicles from young plasma reverse age-related functional declines by improving mitochondrial energy metabolism

In the format provided by the authors and unedited

Supplementary Information for

Small extracellular vesicles from young plasma reverse age-related functional declines by improving mitochondrial energy metabolism

This PDF file includes:

Supplementary Figure 1 to 22.

Supplementary Table 1 to 6.

Supplementary Figures

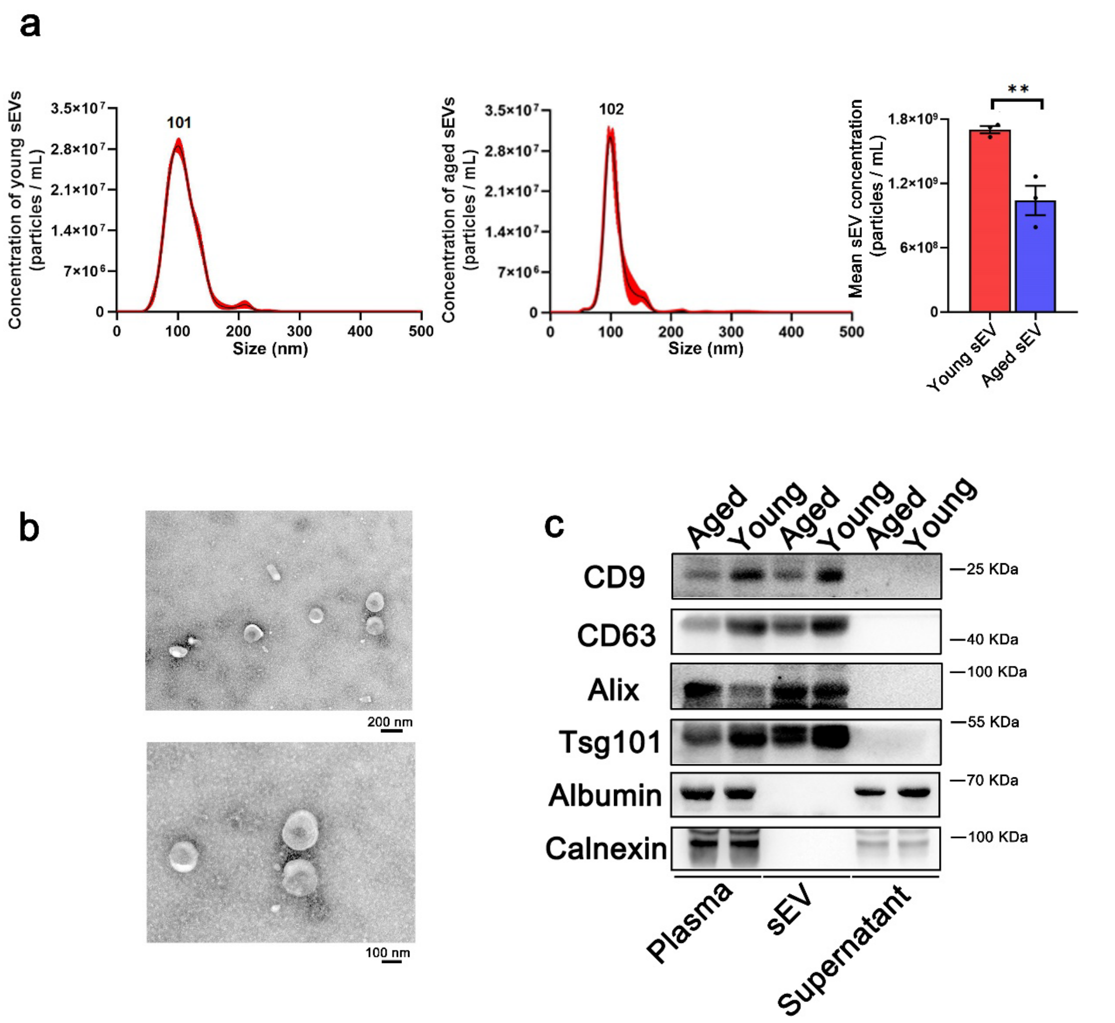

Supplementary Figure 1. Characterization of the properties of purified sEVs. sEVs were purified from the plasma of young (2 months) and aged (20 months) male mice and characterized using NTA, TEM and enrichment of sEV markers. **(a)** Determination of the size distribution and concentration of sEVs using NTA. NTA results revealed that the particles purified from the plasma of young and aged mice displayed a typical sEV size (approximately 100 nm in diameter) and were present in the original plasma of young and aged mice at a similar concentration (1.7×10^9 particles/mL in young plasma *vs.* 1.04×10^9 particles/mL in aged plasma). Left panel: representative NTA images. Right panel: quantitative analysis (n = 3). In conventional NTA analysis, each sample is automatically subjected to three measurements. The red range curve represents the standard deviation of the three measurements, while the black curve corresponds to the mean of these three measurements. **(b)** Representative TEM images of young sEVs. Scale bars: 200 nm in the upper panel and 100 nm in the lower panel. TEM results showed that the majority of particles purified from young mouse plasma exhibited a characteristic round-shaped vesicular morphology and were heterogeneous in size, similar to previously reported exosomes. **(c)** Western blot

analysis of sEV markers (CD9, CD63, Alix and Tsg101), the major plasma protein (Albumin) and endoplasmic reticulum protein (Calnexin) in whole plasma, purified sEVs and sEV-depleted supernatant. An equal amount of total protein was loaded in each lane. Significant enrichment of sEV markers but devoid of Albumin and Calnexin was detected in the sEV fraction. Each experiment was independently repeated three times with similar results for b and c. Significance was determined using two-sided Student's t-test in a. **$P < 0.01$.

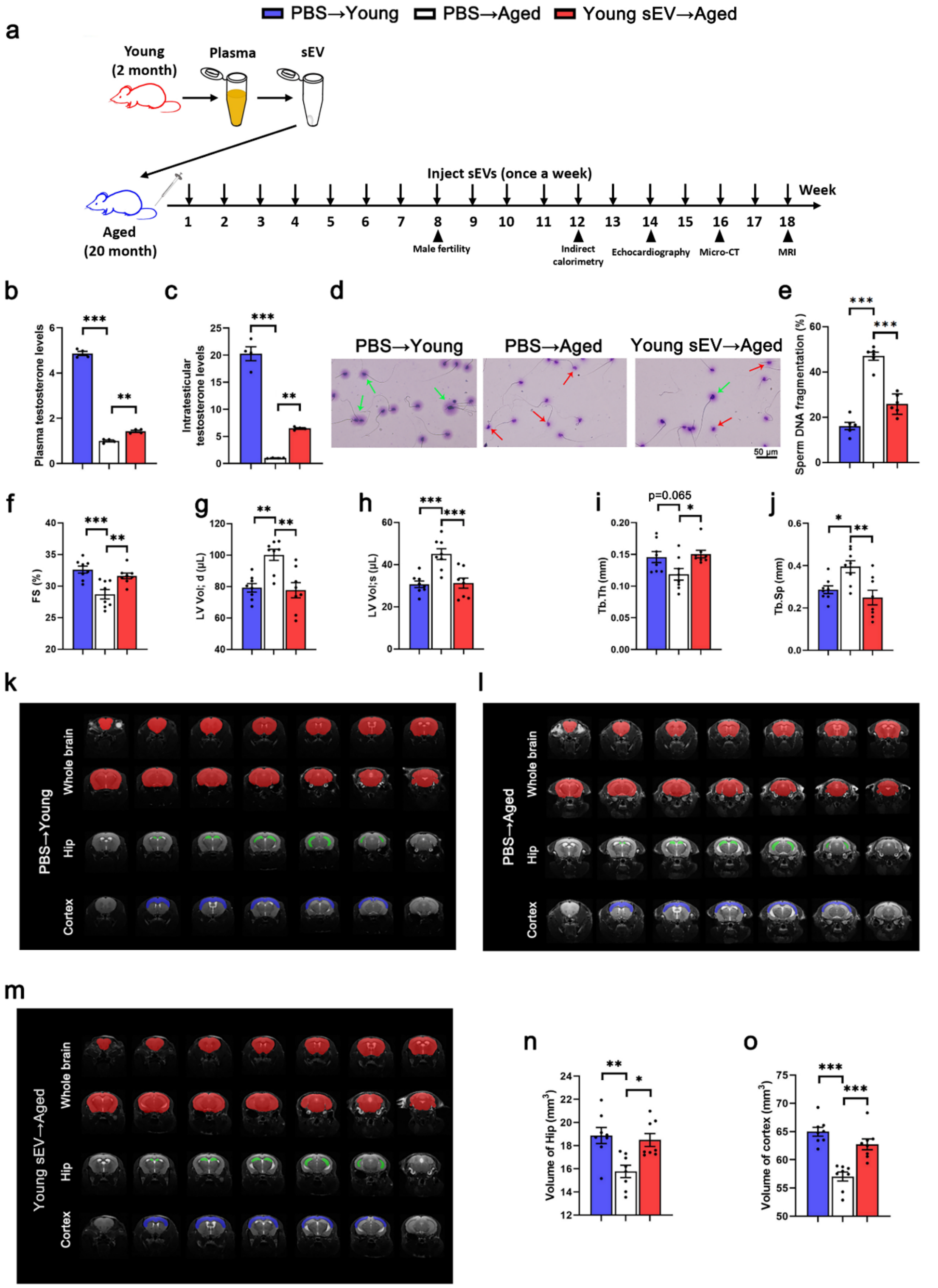

Supplementary Figure 2. Long-term effects of young sEV injection on the whole-body physiology of aged mice. (a) Flow chart of the experimental design. Aged male mice (20 months) were intravenously injected with 200 μL of PBS or young sEVs (1.80 μg of total protein/μL, from 2-month-old male mice) once a week. Young male mice (2 months) were simultaneously injected with PBS to serve as a control group. At different

time points, changes of physiological activities and functions, including sperm quality and male fertility (22-month-old mice receiving 8 injections), metabolic rate and energy expenditure (23-month-old mice receiving 12 injections), cardiac functions (23.5-month-old mice receiving 14 injections), bone microarchitecture (24-month-old mice receiving 16 injections) and brain volume alterations (24.5-month-old mice receiving 18 injections), were evaluated. **(b-c)** Plasma and intratesticular testosterone levels in each group (n = 4). **(d-e)** Assessment of sperm DNA fragmentation with the sperm chromatin dispersion test. Under a bright field microscope, sperm with fragmented DNA fail to produce the characteristic halo of dispersed DNA loops that is observed in sperm with nonfragmented DNA following acid denaturation and removal of nuclear proteins. Therefore, sperm with large halos are considered normal and nonfragmented, whereas sperm with small or no halos are considered to have significant DNA fragmentation. Representative images (green arrows indicate large halos, and red arrows indicate small halos; scale bar, 50 μm) and quantitative data (n = 6) are shown. **(f-h)** Echocardiographic measurements of cardiac dimensions and indices of cardiac function in each group. Quantitation of FS, LV Vol;d and LV Vol;s is shown (n = 8). **(i-j)** Micro-CT analysis of the trabecular microarchitecture of the proximal femur in each group. Quantitative values of Tb.Th and Tb.Sp are shown (n = 8). **(k-o)** MRI-based morphometric analyses of the hippocampus and cortex in each group. Outlines of the whole brain (red), hippocampus (green) and cortex (blue) used for semiautomatic volumetric analyses are depicted in colors. Representative MRI images of the brain in each group are shown, and the absolute volumes of the hippocampus and cortex were calculated (n = 8). Significance was determined using one-way ANOVA followed by Dunnett's multiple comparison test in b, c, e, f, g, h, i, j, n and o. *P < 0.05, **P < 0.01 and ***P < 0.005.

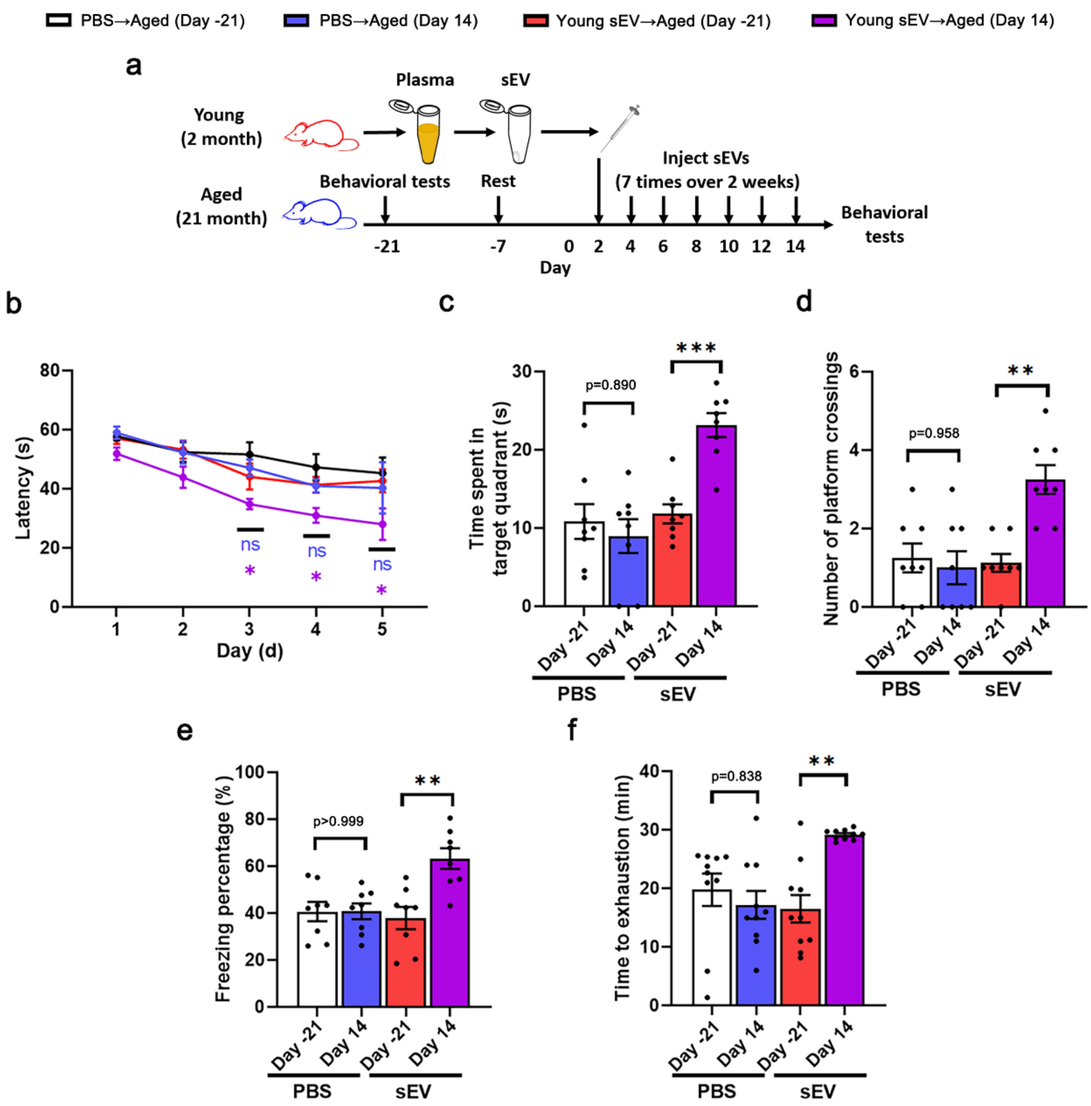

Supplementary Figure 3. Short-term effects of young sEV injection on memory ability and endurance performance of the same batch of aged mice. (a) Flow chart of the experimental design. A batch of aged male mice (21 months) were randomly divided into 2 groups and were intravenously injected with 200 μL of PBS or young sEVs (from 2-month-old male mice) 7 times over 2 weeks. Before (at day -21) and after (at day 14) the 7 injections of PBS or young sEVs, the two groups of aged mice were assessed by a series of behavioral paradigms to determine memory ability and endurance performance. **(b)** The escape latency of each group in the training phase of Morris water maze test (n = 8). Purple and blue asterisks (ns) indicate statistically significant differences between Young sEV→Aged (day 14) *vs.* Young sEV→Aged (day -21) and between PBS→Aged (day 14) *vs.* PBS→Aged (day -21), respectively. **(c-d)** Time spent in the target quadrant and the number of platform crossings by each group in the probe trial of Morris water maze test (n = 8). **(e)** Freezing levels of each group in the contextual fear conditioning test (n = 8). **(f)** Running time to exhaustion for each group in the treadmill running test (n = 10). Significance was determined using

two-sided Student's t-test in b, c, d, e and f. *$P < 0.05$, **$P < 0.01$ and ***$P < 0.005$. ns = not significant.

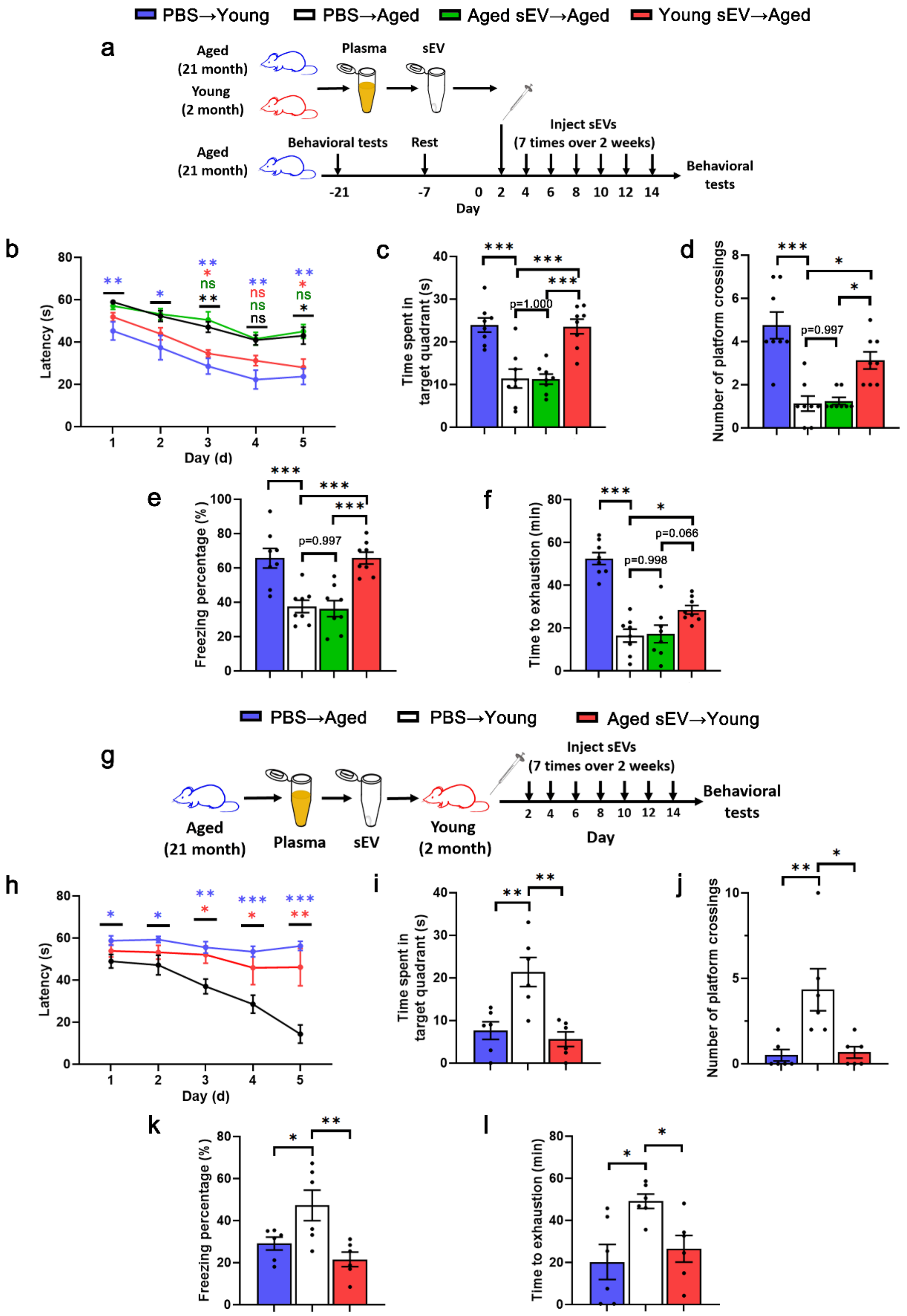

Supplementary Figure 4. Effects of aged sEV injection on memory ability and endurance performance of aged and young mice. (a) Flow chart of the experimental design. Young and aged sEVs were purified from the plasma of young (2 months) and aged male mice (21 months) and resuspended in PBS at a concentration of 1.80 μg of total protein/μL. Aged male mice (21 months) were intravenously injected with 200 μL

of PBS, aged sEVs or young sEVs 7 times over 2 weeks, and then the three groups of aged mice were assessed by a series of behavioral paradigms to determine memory ability and endurance performance. Young male mice (2 months) were simultaneously injected with PBS to serve as a control group. **(b)** The escape latency of each group in the training phase of Morris water maze test (n = 8). Blue, red, green and black asterisks (ns) indicate statistically significant differences between PBS→Young *vs.* PBS→Aged, between Young sEV→Aged *vs.* PBS→Aged, between Aged sEV→Aged *vs.* PBS→Aged and between Young sEV→Aged *vs.* Aged sEV→Aged, respectively. **(c-d)** Time spent in the target quadrant and the number of platform crossings by each group in the probe trial of Morris water maze test (n = 8). **(e)** Freezing levels of each group in the contextual fear conditioning test (n = 8). **(f)** Running time to exhaustion for each group in the treadmill running test (n = 8). **(g)** Flow chart of the experimental design. Aged sEVs were purified from the plasma of aged male mice (21 months) and resuspended in PBS at a concentration of 1.80 μg of total protein/μL. Young male mice (2 months) were intravenously injected with 200 μL of PBS or aged sEVs 7 times over 2 weeks, and then the two groups of young mice were assessed by a series of behavioral paradigms to determine memory ability and endurance performance. Aged male mice (21 months) were simultaneously injected with PBS to serve as a control group. **(h)** The escape latency of each group in the training phase of Morris water maze test (n = 6). Blue and red asterisks indicate statistically significant differences between PBS→Aged *vs.* PBS→Young and between Aged sEV→Young *vs.* PBS→Young, respectively. **(i-j)** Time spent in the target quadrant and the number of platform crossings by each group in the probe trial of Morris water maze test (n = 6). **(k)** Freezing levels of each group in the contextual fear conditioning test (n = 6). **(l)** Running time to exhaustion for each group in the treadmill running test (n = 10). Significance was determined using one-way ANOVA followed by Dunnett's multiple comparison test in b, c, d, e, f, h, i, j, k and l. $*P < 0.05$, $**P < 0.01$ and $***P < 0.005$. ns = not significant.

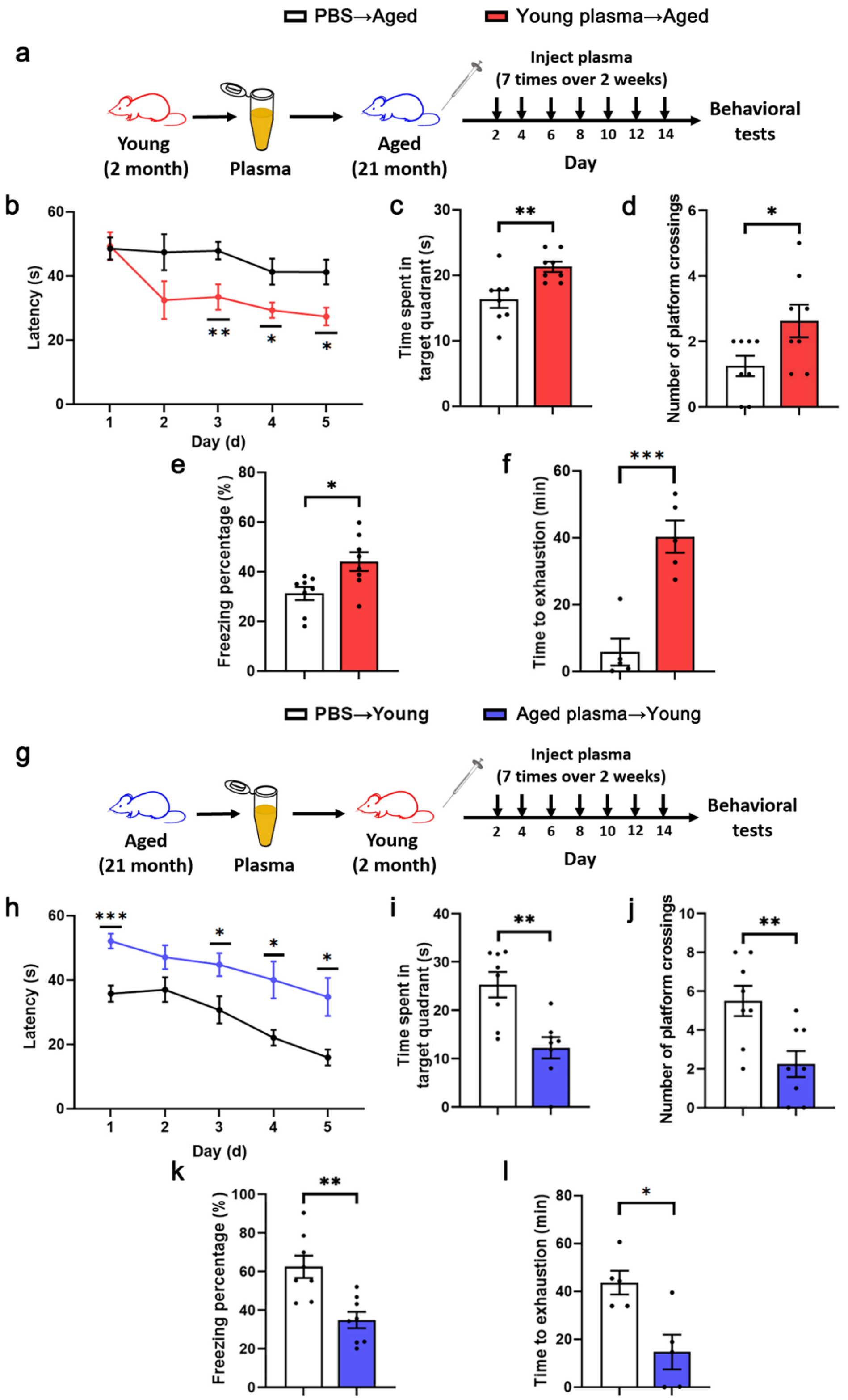

Supplementary Figure 5. Short-term effects of young/aged plasma injection on the cognitive function and muscle endurance of aged/young mice. (a) Flow chart of the experimental design. Aged male mice (21 months) were intravenously injected with 200 μL of PBS or young plasma (from 2-month-old male mice) 7 times over 2 weeks, and then the two groups of aged mice were assessed by a series of behavioral paradigms

to determine memory ability and endurance performance. **(b)** The escape latency of each group in the training phase of Morris water maze test (n = 9 for PBS→Aged; n = 8 for Young sEV→Aged). **(c-d)** Time spent in the target quadrant and the number of platform crossings by each group in the probe trial of Morris water maze test (n = 8). **(e)** Freezing levels of each group in the contextual fear conditioning test (n = 8). **(f)** Running time to exhaustion for each group in the treadmill running test (n = 5). **(g)** Flow chart of the experimental design. Young male mice (2 months) were intravenously injected with 200 μL of PBS or aged plasma (from 21-month-old male mice) 7 times over 2 weeks, and then the two groups of young mice were assessed by a series of behavioral paradigms to determine memory ability and endurance performance. **(h)** The escape latency of each group in the training phase of Morris water maze test (n = 8). **(i-j)** Time spent in the target quadrant and the number of platform crossings by each group in the probe trial of Morris water maze test (n = 8). **(k)** Freezing levels of each group in the contextual fear conditioning test (n = 8). **(l)** Running time to exhaustion for each group in the treadmill running test (n = 5). Significance was determined using two-sided Student's t-test in b, c, d, e, f, h, i, j, k and l. *P < 0.05, **P < 0.01 and ***P < 0.005.

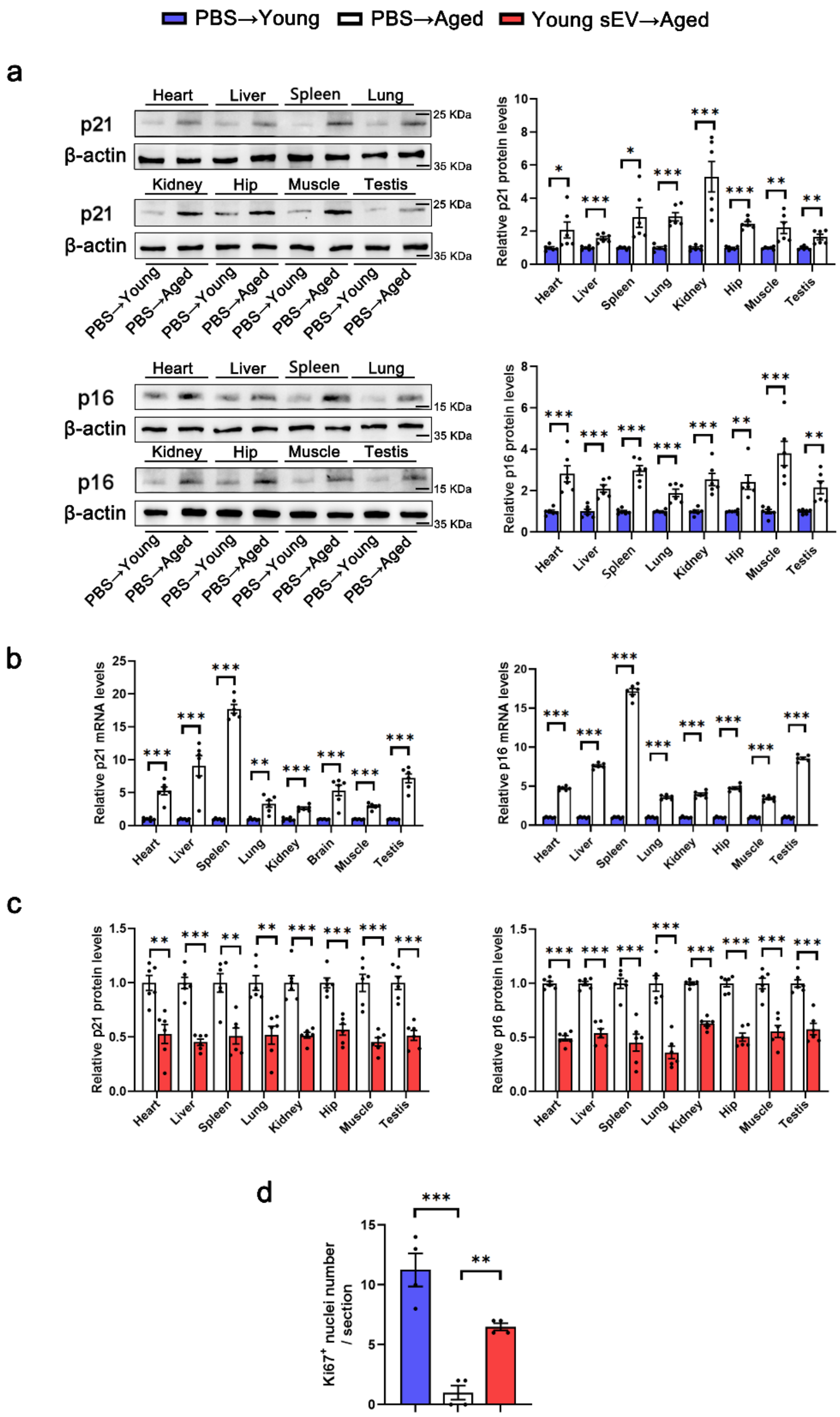

Supplementary Figure 6. Short-term effects of young sEV injection on the senescent phenotypes of aged mice. Aged male mice (21 months) were intravenously injected with 200 μL of PBS or young sEVs (from 2-month-old male mice) 7 times over 2 weeks. Young male mice (2 months) were simultaneously injected with PBS to serve as a control group. **(a)** Western blot analysis of p21 and p16 protein levels in the heart, liver, spleen, lung, kidney, hippocampus, muscle and testis derived from young and aged mice injected with PBS 7 times over 2 weeks. Left panel: representative Western blots. Right panel: densitometric analysis (n = 6). **(b)** Quantitative RT–PCR

analysis of p21 and p16 mRNA levels in the heart, liver, spleen, lung, kidney, hippocampus, muscle and testis derived from young and aged mice injected with PBS 7 times over 2 weeks (n = 6). **(c)** Western blot analysis of p21 and p16 protein levels in the heart, liver, spleen, lung, kidney, hippocampus, muscle and testis derived from aged mice injected with PBS or young sEVs. Densitometric analysis are shown (n = 6). **(d)** Immunohistochemistry staining of Ki67 in the hippocampus sections. Quantification of Ki67 staining intensity in the hippocampus sections are shown (n = 4). Significance was determined using two-sided Student's t-test in a, b and c and using one-way ANOVA followed by Dunnett's multiple comparison test in d. *P < 0.05, **P < 0.01 and ***P < 0.005.

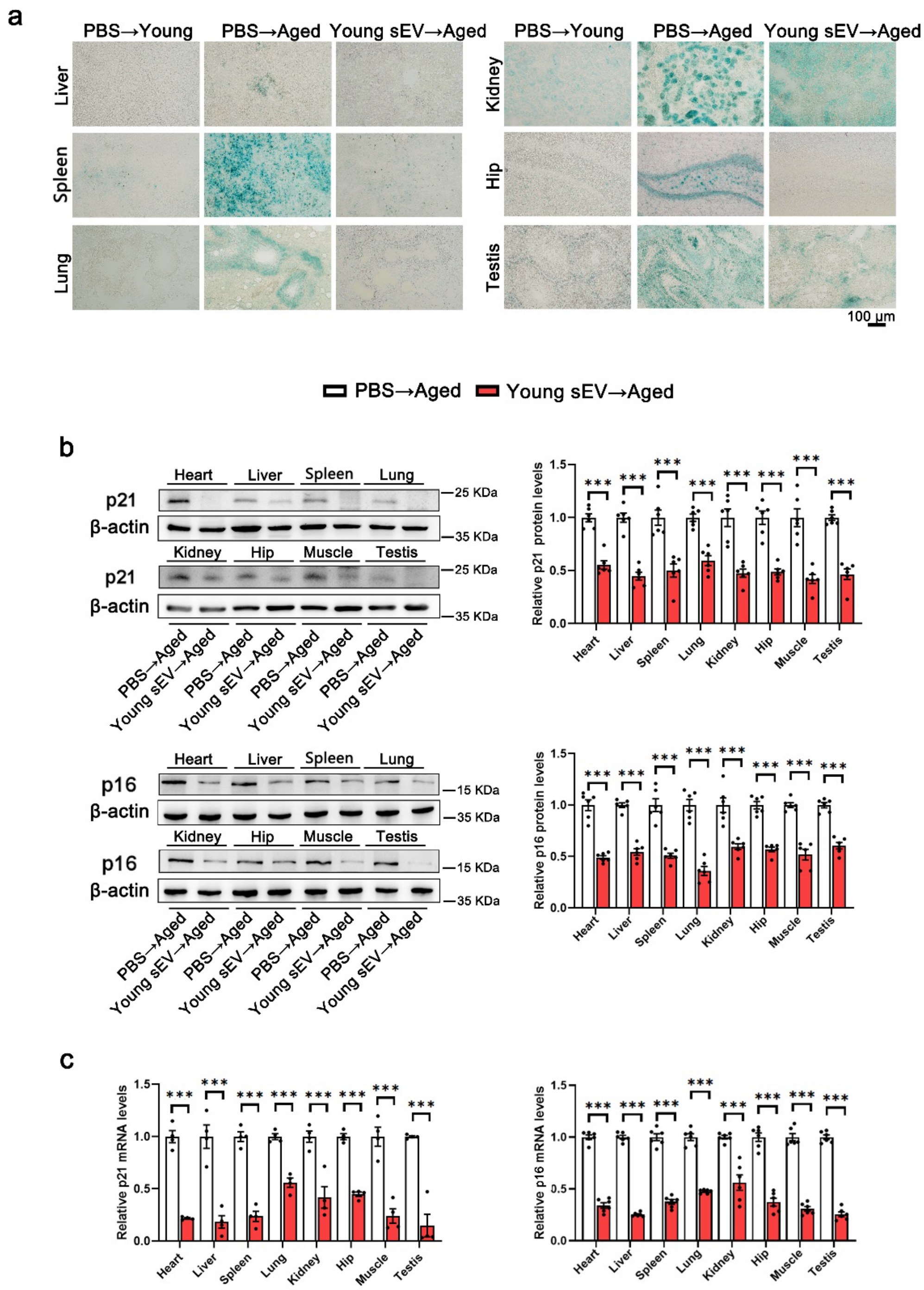

Supplementary Figure 7. Long-term effects of young sEV injection on the senescent phenotypes of aged mice. Aged male mice (20 months) were intravenously injected with 200 μL of PBS or young sEVs (1.80 μg of total protein/μL, from 2-month-old male mice) once a week for 15 weeks. Young male mice (2 months) were simultaneously injected with PBS to serve as a control group. **(a)** Representative images of SA-β-Gal staining in the sections of liver, spleen, lung, kidney, hippocampus and testis derived from aged mice injected with PBS or young sEVs. The tissue sections derived from young mice injected with PBS were taken as a control. Scale bar, 100 μm.

(b) Western blot analysis of p21 and p16 protein levels in the heart, liver, spleen, lung, kidney, hippocampus, muscle and testis derived from aged mice injected with PBS or young sEVs. Left panel: representative Western blots. Right panel: densitometric analysis (n = 6). **(c)** Quantitative RT–PCR analysis of p21 and p16 mRNA levels in the heart, liver, spleen, lung, kidney, hippocampus, muscle and testis derived from aged mice injected with PBS or young sEVs (n = 4 for p21; n = 6 for p16). Each experiment was independently repeated four times with similar results in a. Significance was determined using two-sided Student's t-test in b and c. *$P < 0.05$, **$P < 0.01$ and ***$P < 0.005$.

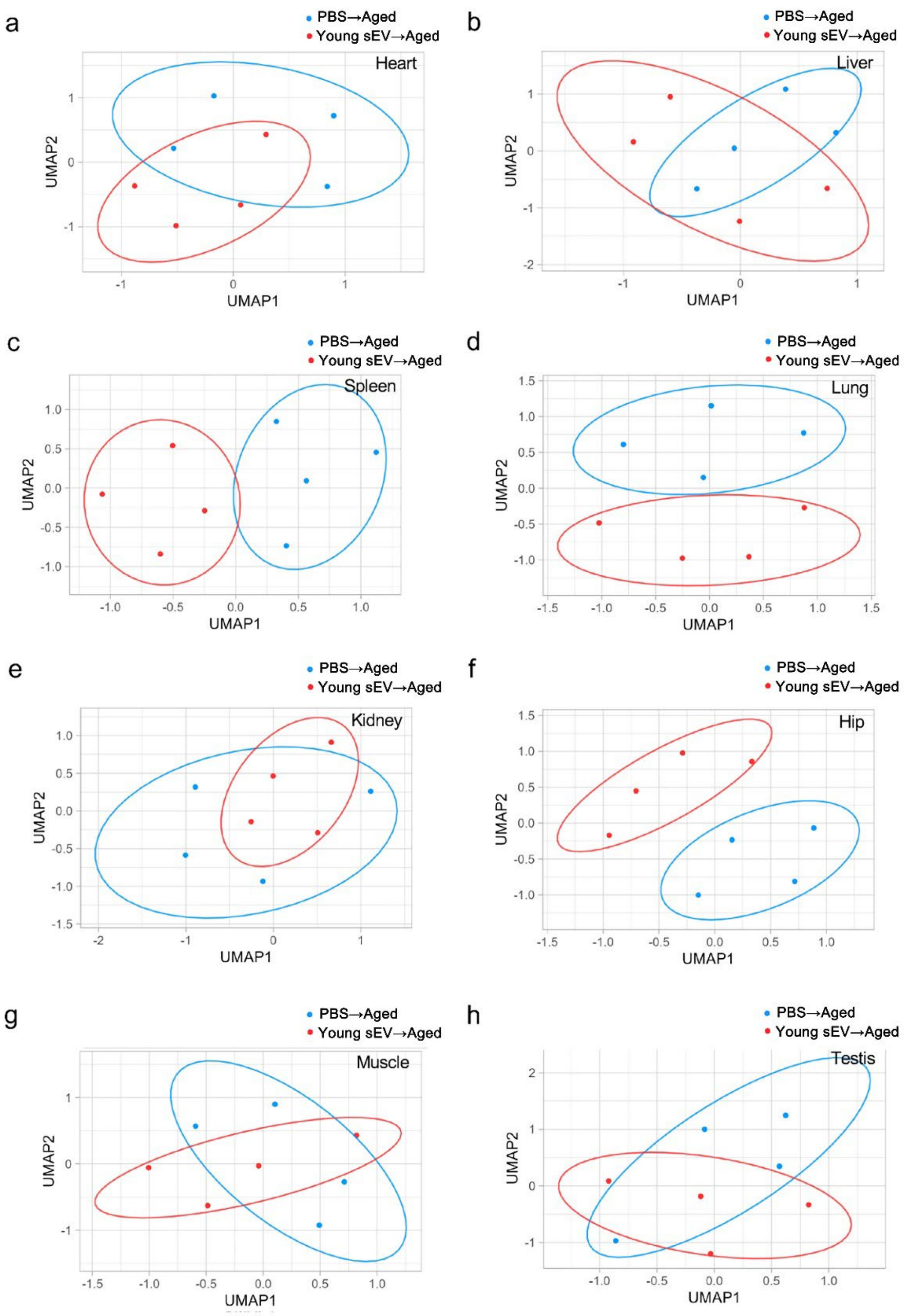

Supplementary Figure 8. UMAP plot of the iTRAQ quantitative proteomic data in eight tissues from PBS- and young sEV-injected aged mice. Each dot represents the overall protein expression in each tissue. The distance between dots indicates their dissimilarity.

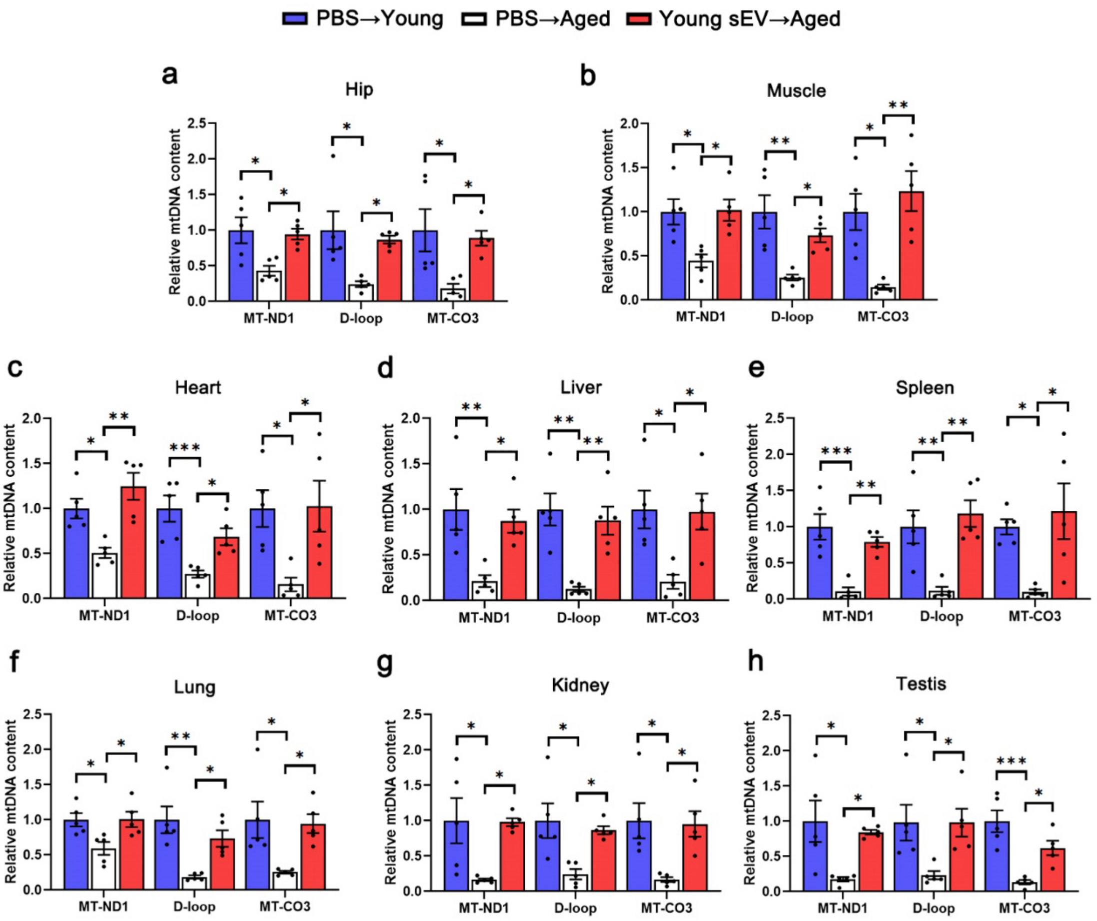

Supplementary Figure 9. Young sEV treatment mitigates the loss of mtDNA content in various tissues of aged mice. Aged male mice (21 months) were intravenously injected with 200 μL of PBS or young sEVs (from 2-month-old male mice) 7 times over 2 weeks. Young male mice (2 months) were simultaneously injected with PBS to serve as a control group. Mitochondrially encoded NADH dehydrogenase 1 (MT-ND1), cytochrome c oxidase III (MT-CO3) and D-loop region, normalized to β2-microglobulin (β2-MG), were used to measure relative mtDNA content. **(a-h)** Relative mtDNA content in the hippocampus, muscle, heart, liver, spleen, lung, kidney and testis of each group (n = 5). Significance was determined using one-way ANOVA followed by Dunnett's multiple comparison test in a-h. *P < 0.05, **P < 0.01 and ***P < 0.005.

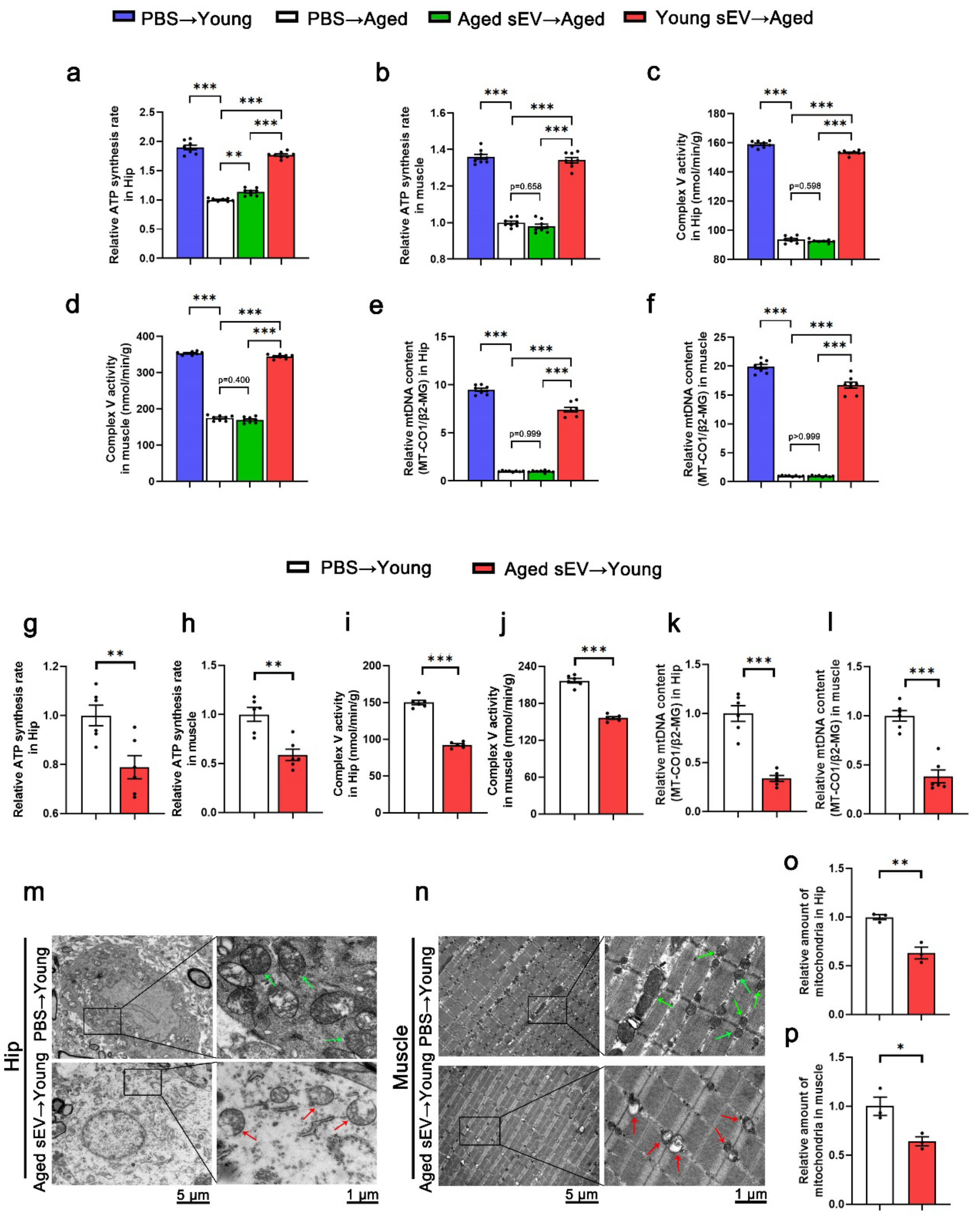

Supplementary Figure 10. Effects of aged sEV injection on metabolic phenotypes of aged and young mice. Aged male mice (21 months) were intravenously injected with 200 μL of PBS, aged sEVs (from 21-month-old male mice) or young sEVs (from 2-month-old male mice) 7 times over 2 weeks, and then the three groups of aged mice were subjected to assessments of mitochondrial functional parameters and metabolic phenotypes. Young male mice (2 months) were simultaneously injected with PBS to serve as a control group. **(a-b)** ATP synthesis rates in the hippocampus and muscle of each group (n = 8). **(c-d)** Mitochondrial complex V activity in the hippocampus and

muscle of each group (n = 8). **(e-f)** Relative mtDNA content (MT-CO1/β2-MG) in the hippocampus and muscle of each group (n = 8). **(g-p)** Young male mice (2 months) were intravenously injected with 200 μL of PBS or aged sEVs (from 21-month-old male mice) 7 times over 2 weeks, and then the two groups of young mice were subjected to assessments of mitochondrial functional parameters and metabolic phenotypes. **(g-h)** ATP synthesis rates in the hippocampus and muscle of each group (n = 6). **(i-j)** Mitochondrial complex V activity in the hippocampus and muscle of each group (n = 6). **(k-l)** Relative mtDNA content (MT-CO1/β2-MG) in the hippocampus and muscle of each group (n = 6). **(m-n)** Representative TEM images showing the structure and density of mitochondria in the hippocampus and muscle of each group. Normal mitochondria are round or oval-shaped and contain well-defined cristae, whereas aged mitochondria become swollen, vacuolated and even broken, with cracked mitochondrial cristae. The green arrow indicates morphologically normal mitochondria, and the red arrow indicates morphologically damaged mitochondria. Scale bars: 5 μm in the left panel and 1 μm in the right panel. **(o-p)** Quantification of the numbers of mitochondria in the sections (at low magnification) of hippocampus and muscle (n = 3). Significance was determined using one-way ANOVA followed by Dunnett's multiple comparison test in a, b, c, d, e, f and using two-sided Student's t-test in g, h, i, j, k, l, o and p. $^*P < 0.05$, $^{**}P < 0.01$ and $^{***}P < 0.005$.

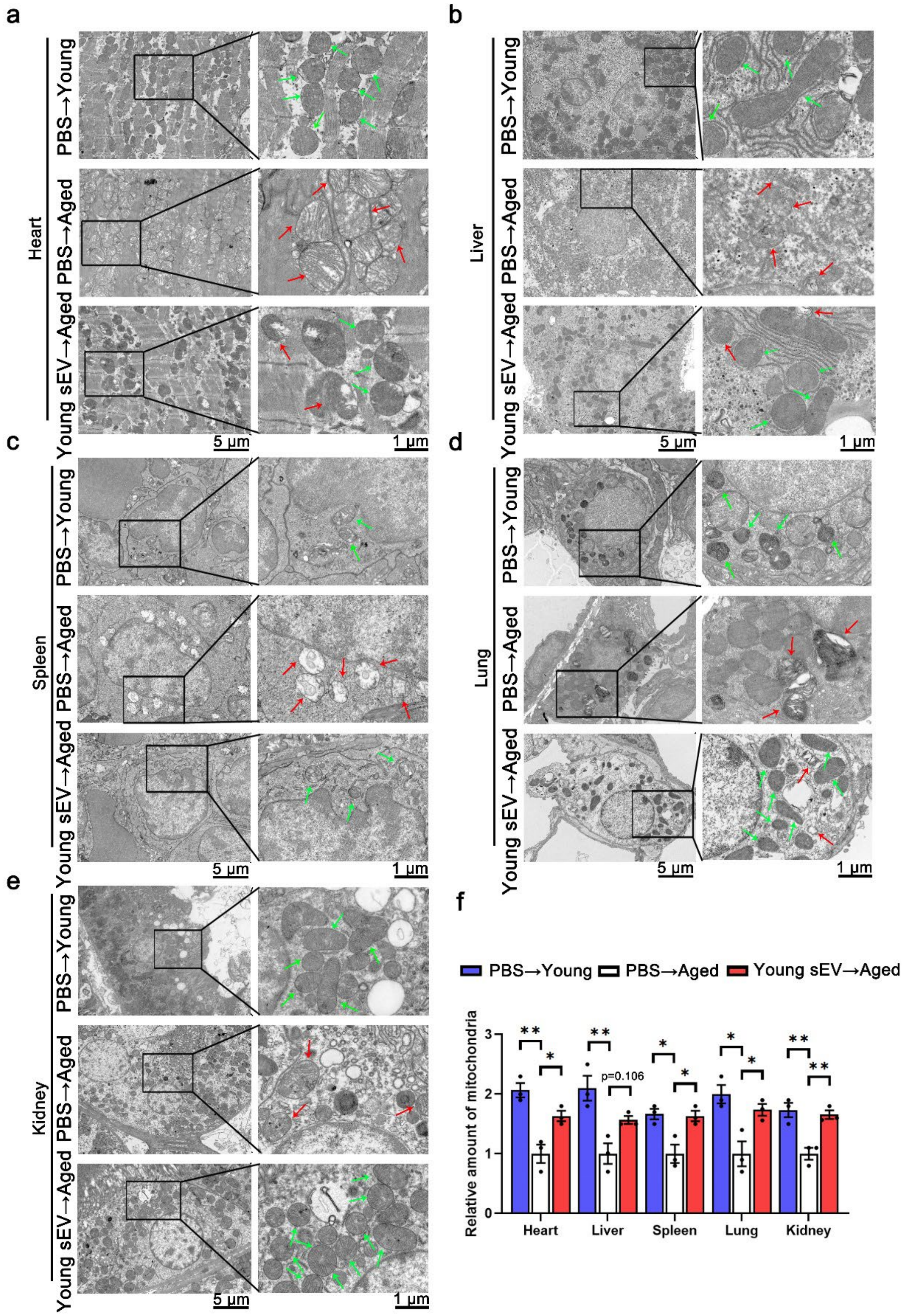

Supplementary Figure 11. The ultrastructure of mitochondria in various tissues of aged mice after treatment with young sEVs. Aged male mice (21 months) were intravenously injected with 200 μL of PBS or young sEVs (from 2-month-old male mice) 7 times over 2 weeks. Young male mice (2 months) were simultaneously injected with PBS to serve as a control group. TEM was employed to visualize the mitochondria

at the ultrastructural level. **(a-e)** Representative TEM images showing the structure and density of mitochondria in the heart, liver, spleen, lung and kidney of each group. Normal mitochondria are round or oval-shaped and contain well-defined cristae, whereas aged mitochondria become swollen, vacuolated and even broken, with cracked mitochondrial cristae. The green arrow indicates morphologically normal mitochondria, and the red arrow indicates morphologically damaged mitochondria. Scale bars: 5 μm in the left panel and 1 μm in the right panel. **(f)** Quantification of the numbers of mitochondria in the sections (at low magnification) of heart, liver, spleen, lung and kidney (n = 3). Significance was determined using one-way ANOVA followed by Dunnett's multiple comparison test in f. *P < 0.05 and **P < 0.01.

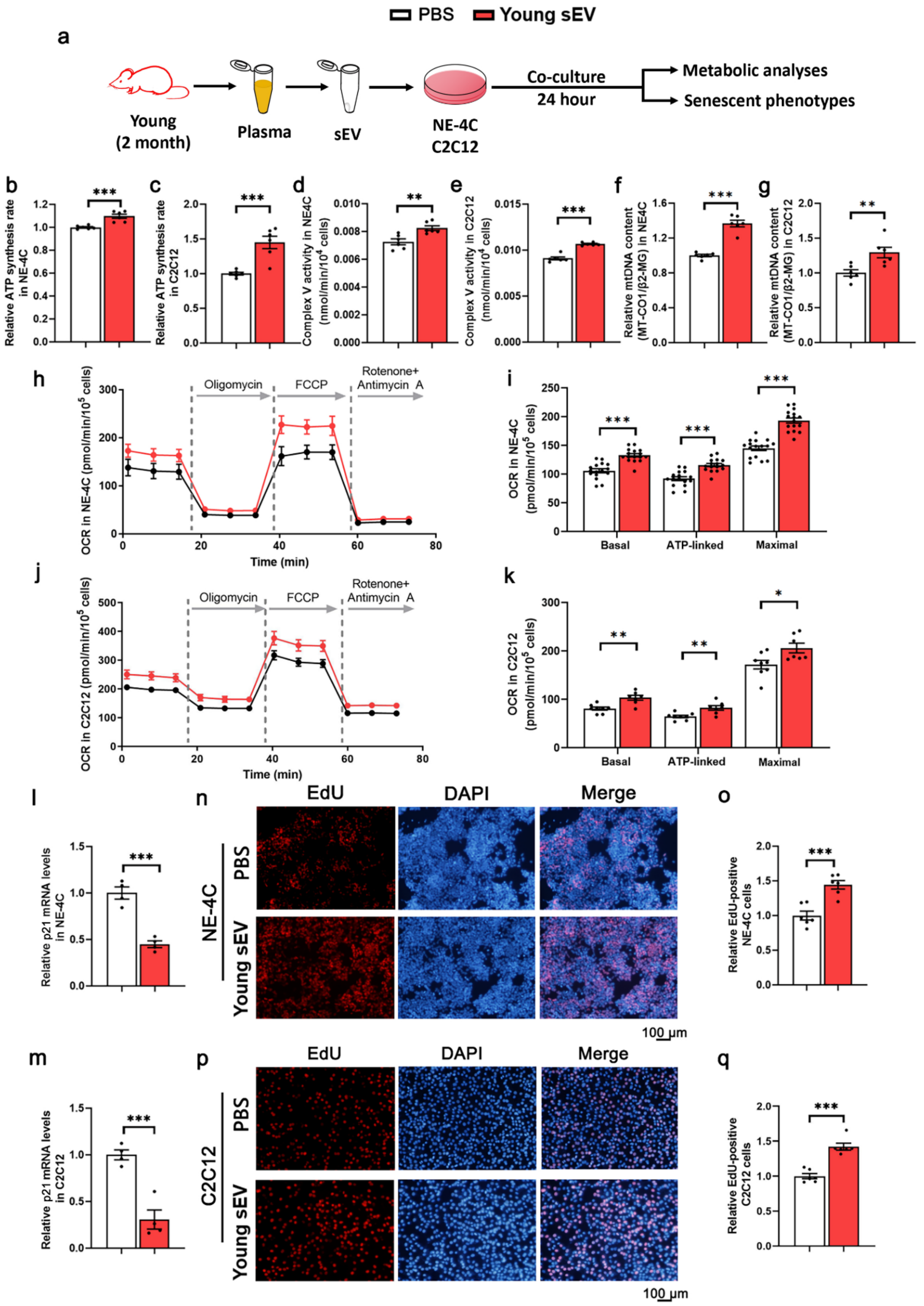

Supplementary Figure 12. Young sEV treatment improves mitochondrial functions and attenuates senescent phenotypes in cultured cells. (a) Flow chart of the experimental design. NE-4C or C2C12 cells (1×10^6 cells) were incubated with 100 μL of PBS or young sEVs (from 2-month-old male mice) for 24 hours, and then the cells were subjected to assessments of mitochondrial functional parameters and senescent phenotypes. **(b-c)** ATP synthesis rates in NE-4C and C2C12 cells (n = 6). **(d-**

e) Mitochondrial complex V activity in NE-4C and C2C12 cells (n = 6). **(f-g)** Relative mtDNA content (MT-CO1/β2-MG) in NE-4C and C2C12 cells (n = 6). **(h-k)** Measurement of OCR in NE-4C and C2C12 cells. After measurement of basal OCR, oligomycin, FCCP, and rotenone + antimycin A were sequentially added, and the alterations in OCR were recorded and normalized to cell number. Quantification of the basal OCR, ATP-coupled OCR and maximal OCR is shown (NE-4C, n = 16; C2C12, n = 8 for PBS, n = 7 for Young sEV). **(l-m)** Quantitative RT–PCR analysis of P21 mRNA levels in NE-4C and C2C12 cells (n = 4). **(n-q)** EdU incorporation assay showing the proportion of proliferating cells in NE-4C and C2C12 cells. Representative images (scale bar, 100 μm) and quantitative analysis of the percentage of EdU-positive cells (n = 6) are shown. Significance was determined using two-sided Student's t-test in b, c, d, e, f, g, i, k, l, m, o and q. *P < 0.05, **P < 0.01 and ***P < 0.005.

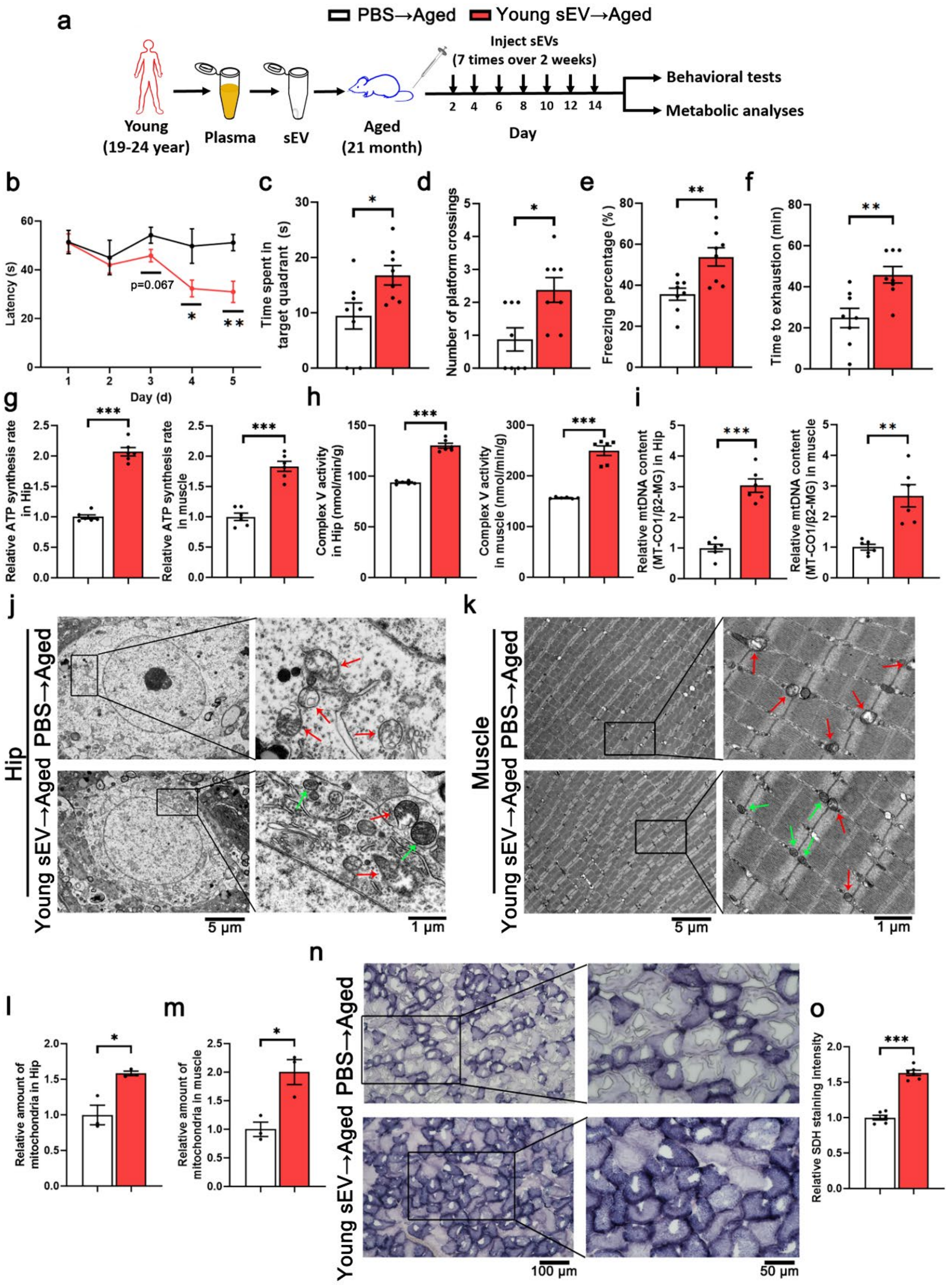

Supplementary Figure 13. Human sEVs derived from the plasma of young donors improve physiological functions and counteract mitochondrial deficiency in aged mice. (a) Flow chart of the experimental design. Young human sEVs were purified from the plasma of young male donors (19-24 years) and resuspended in PBS at a concentration of 1.80 μg of total protein/μL. Aged male mice (21 months) were intravenously injected with 200 μL of PBS or young human sEVs 7 times over 2 weeks, and then the two groups of aged mice were monitored to determine behavioral

performance and mitochondrial functional parameters. **(b)** The escape latency of each group in the training phase of Morris water maze test (n = 8). **(c-d)** Time spent in the target quadrant and the number of platform crossings by each group in the probe trial of Morris water maze test (n = 8). **(e)** Freezing levels of each group in the contextual fear conditioning test (n = 8). **(f)** Running time to exhaustion for each group in the treadmill running test (n = 8). **(g)** ATP synthesis rates in the hippocampus and muscle of each group (n = 6). **(h)** Mitochondrial complex V activity in the hippocampus and muscle of each group (n = 6). **(i)** Relative mtDNA content (MT-CO1/β2-MG) in the hippocampus and muscle of each group (n = 6). **(j-k)** Representative TEM images showing the structure and density of mitochondria in the hippocampus and muscle of each group. The green arrow indicates morphologically normal mitochondria, and the red arrow indicates morphologically damaged mitochondria. Scale bars: 5 μm in the left panel and 1 μm in the right panel. **(l-m)** Quantification of the amounts of mitochondria in the sections (at low magnification) of hippocampus and muscle (n = 3). **(n-o)** SDH staining of the muscle fibers in each group. Representative images (scale bars: 100 μm in the left panel and 50 μm in the right panel) and quantification of SDH staining intensity (n = 6) are shown. Significance was determined using two-sided Student's t-test in b, c, d, e, f, g, h, i, l, m and o. *$P < 0.05$, **$P < 0.01$ and ***$P < 0.005$.

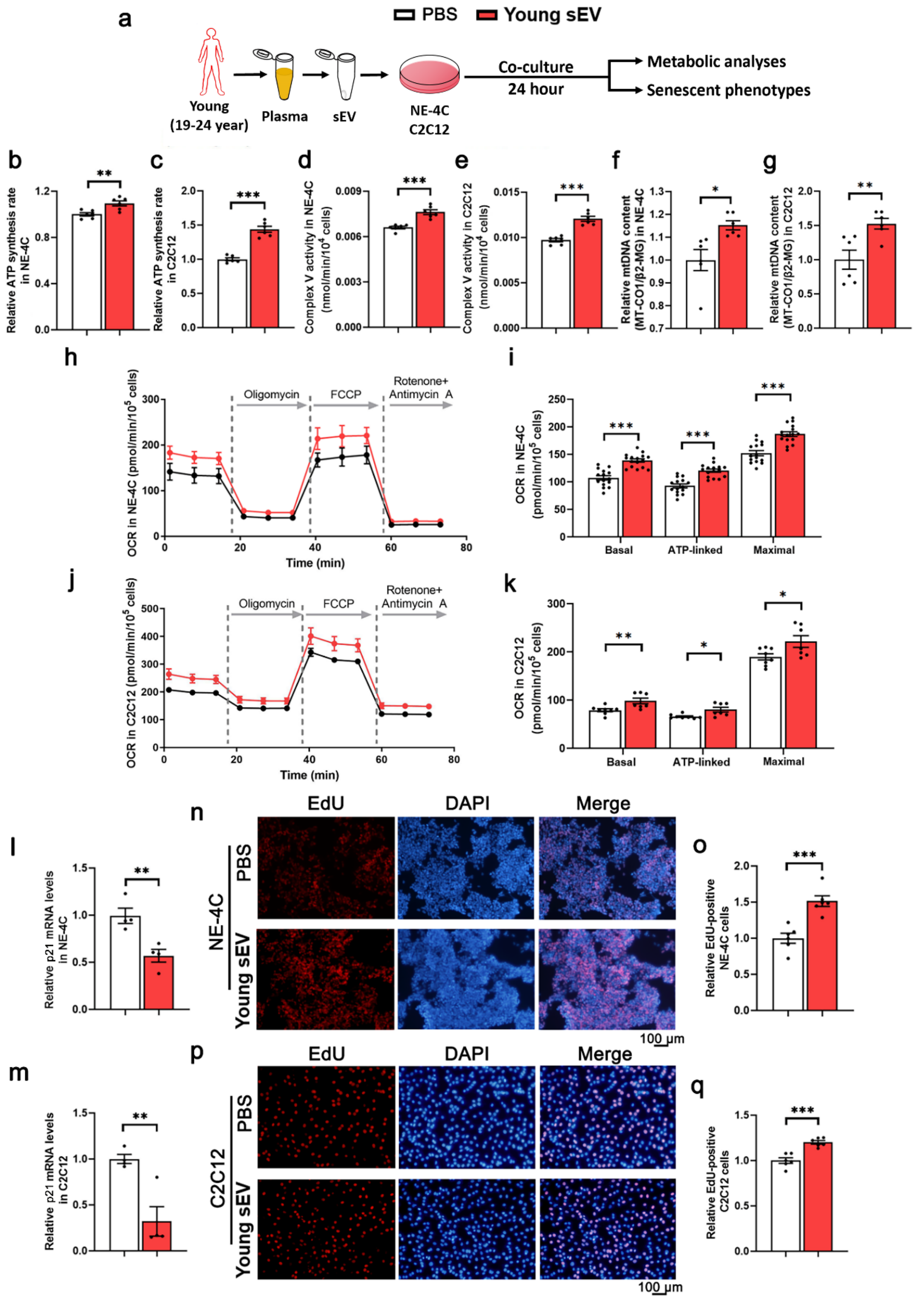

Supplementary Figure 14. Human sEVs derived from the plasma of young donors improve mitochondrial functions and attenuate senescent phenotypes in cultured cells. (a) Flow chart of the experimental design. NE-4C or C2C12 cells (1 × 10^6 cells) were incubated with 100 μL of PBS or sEVs derived from the plasma of young male donors for 24 hours, and then the cells were subjected to assessments of mitochondrial functional parameters and senescent phenotypes. **(b-c)** ATP synthesis rates in NE-4C

and C2C12 cells (n = 6). **(d-e)** Mitochondrial complex V activity in NE-4C and C2C12 cells (n = 6). **(f-g)** Relative mtDNA content (MT-CO1/β2-MG) in NE-4C and C2C12 cells (n = 6). **(h-k)** Measurement of OCR in NE-4C and C2C12 cells. After measurement of basal OCR, oligomycin, FCCP, and rotenone + antimycin A were sequentially added, and the alterations in OCR were recorded and normalized to cell number. Quantification of the basal OCR, ATP-coupled OCR and maximal OCR is shown (NE-4C, n = 15 for PBS, n = 16 for Young sEV; C2C12, n = 8 for PBS, n = 7 for Young sEV). **(l-m)** Quantitative RT–PCR analysis of p21 mRNA levels in NE-4C and C2C12 cells (n = 4). **(n-q)** EdU incorporation assay showing the proportion of proliferating cells in NE-4C and C2C12 cells. Representative images (scale bar, 100 μm) and quantitative analysis of the percentage of EdU-positive cells (n = 6) are shown. Significance was determined using two-sided Student's t-test in b, c, d, e, f, g, i, k, l, m, o and q. $^{*}P < 0.05$, $^{**}P < 0.01$ and $^{***}P < 0.005$.

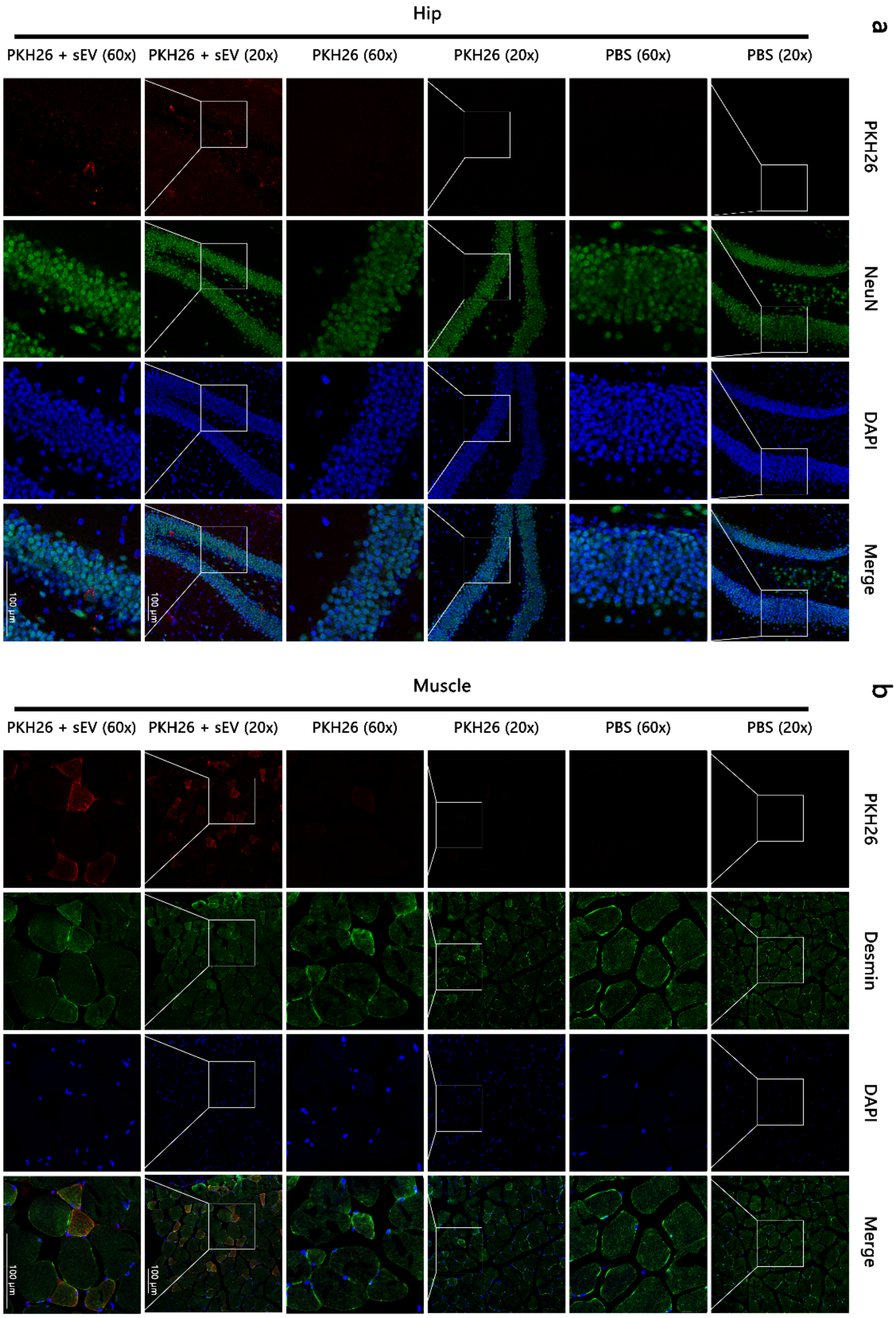

Supplementary Figure 15. Tracking of the delivery of fluorescently labeled young sEVs into hippocampus and muscle of aged mice. Young sEVs were purified from the plasma of young male mice (2 months) and stained with PKH26, and then the fluorescently labeled sEVs were intravenously injected into aged male mice (21 months). After treatment, aged mice were sacrificed, and fluorescence confocal microscopy was applied to detect the red fluorescent signals in frozen sections of hippocampus and muscle. Aged mice were solely injected with PBS or PKH26 dye as controls. **(a-b)** Representative images of microscopic fields showing PKH26-positive

cells in the hippocampus and muscle. PKH26-stained cells and DAPI-stained nuclei are shown in red and blue, respectively. The sections were also stained with specific tissue markers (positive signals are shown in green), including neuron-specific nucleoprotein (NeuN) for hippocampus and Desmin for muscle. Magnification, 20 × and 60 ×. Scale bar, 100 μm. Each experiment was independently repeated three times with similar results in a and b.

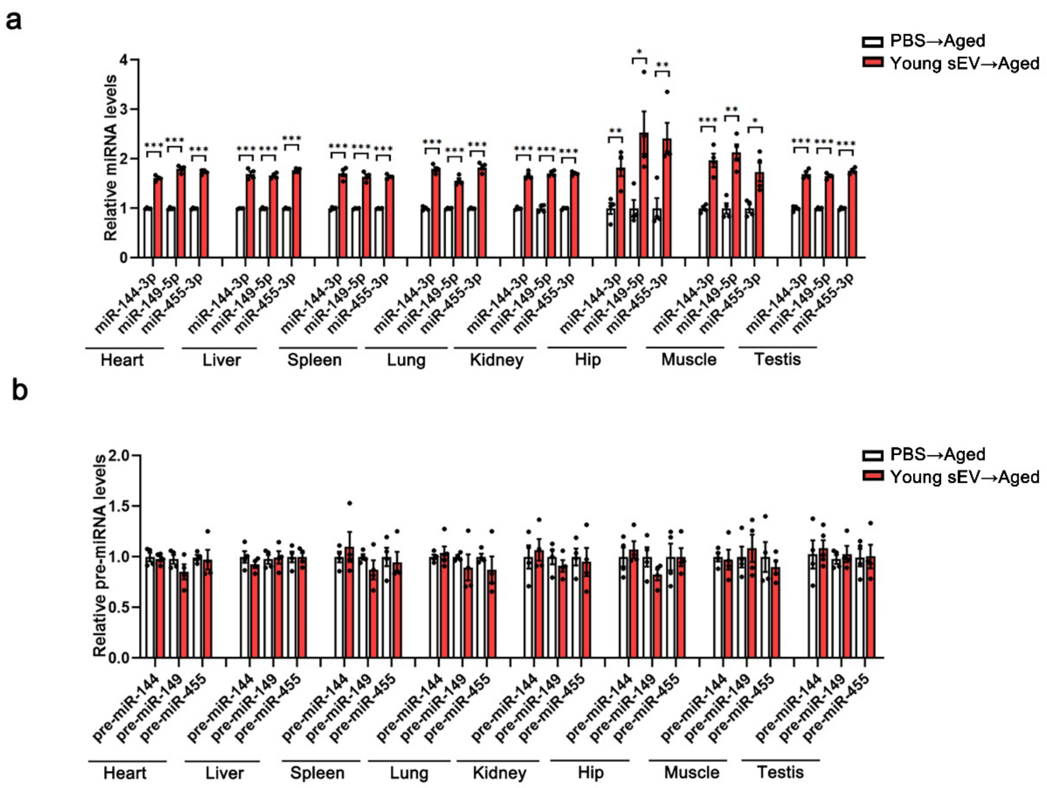

Supplementary Figure 16. Uptake of sEV miRNAs by aged tissues following the injection of young plasma sEVs into aged mice. (a) Quantitative RT–PCR analysis of miR-144-3p, miR-149-5p and miR-455-3p levels in the heart, liver, spleen, lung, kidney, hippocampus, muscle and testis of aged mice injected with 200 μL of PBS or young sEVs (from 2-month-old male mice) 7 times over 2 weeks. Fold changes of miRNAs in young sEV-injected mice relative to PBS-injected mice were determined (n = 4). **(b)** Quantitative RT–PCR analysis of pre-miR-144, pre-miR-149 and pre-miR-455 levels in the heart, liver, spleen, lung, kidney, hippocampus, muscle and testis of aged mice injected with 200 μL of PBS or young sEVs (from 2-month-old male mice) 7 times over 2 weeks. Fold changes of pre-miRNAs in young sEV-injected mice relative to PBS-injected mice were determined (n = 4). Significance was determined using two-sided Student's t-test in a-b. *P < 0.05, **P < 0.01 and ***P < 0.005.

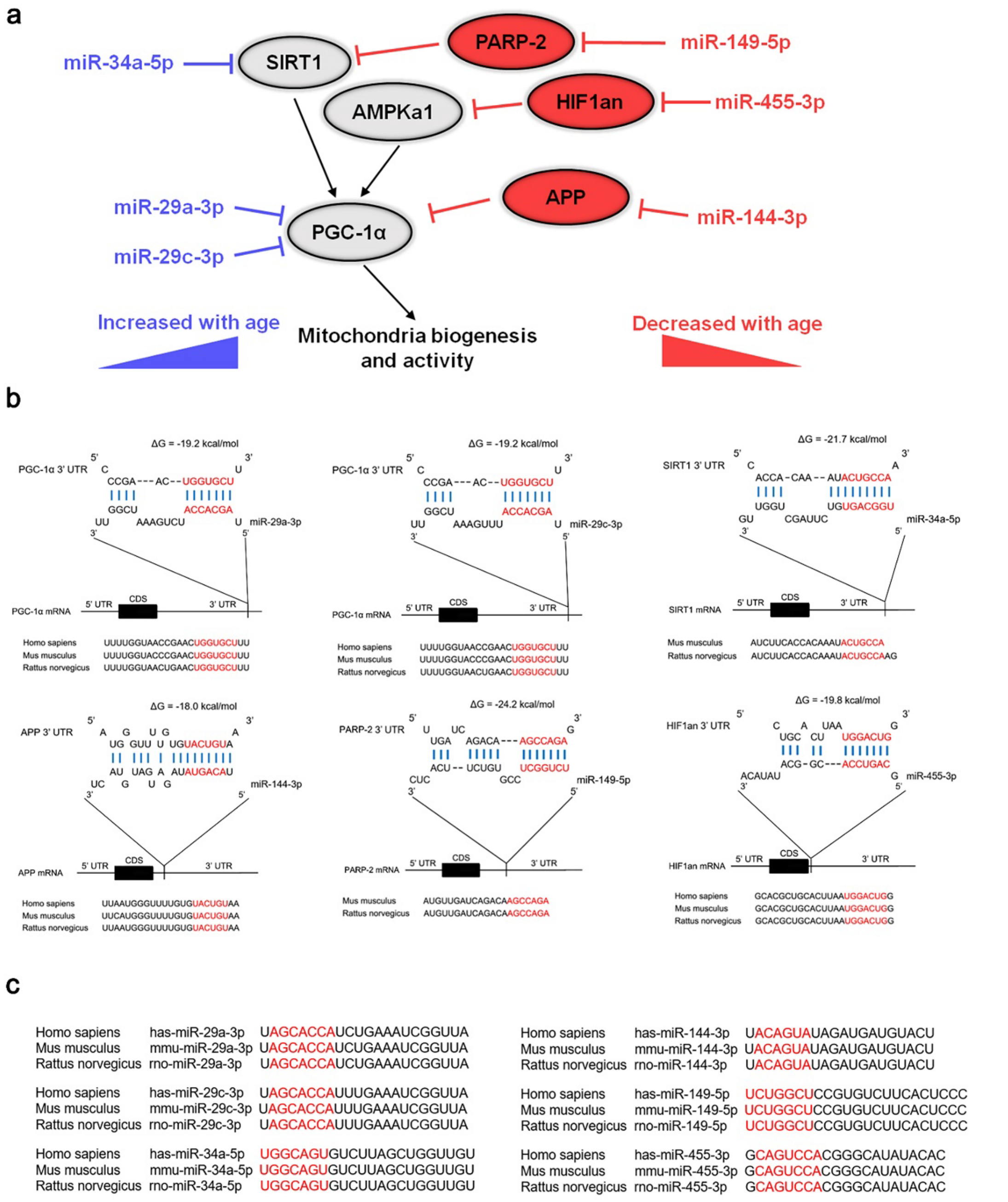

Supplementary Figure 17. PGC-1α is a direct or indirect downstream target of miR-29a-3p, miR-29c-3p, miR-34a-5p, miR-144-3p, miR-149-5p and miR-455-3p. **(a)** Putative working model and potential effects of the miR-29a-3p, miR-29c-3p and miR-34a-5p group and the miR-144-3p, miR-149-5p and miR-455-3p group on PGC-1α expression and mitochondrial functions. miR-29 family (miR-29a-3p and miR-29c-3p) directly downregulates the target gene PGC-1α, which in turn controls mitochondrial biogenesis and homeostasis. Meanwhile, miR-34a-5p directly targets and decreases Sirtuin1 (SIRT1) expression, which increases acetylation of the SIRT1 target transcriptional regulator PGC-1α, eventually resulting in decreased transcriptional activities of PGC-1α. On the other hand, while β-amyloid precursor protein (APP) shows inhibitory effects on the expression of PGC-1α, miR-144-3p

inhibits the expression of APP to increase cellular ATP levels and mtDNA copy numbers. Likewise, poly (ADP-ribose) polymerase-2 (PARP-2) is a direct target gene of miR-149-5p, and miR-149-5p inhibits PARP-2 expression and increases SIRT1 activity that subsequently enhances mitochondrial function and biogenesis via PGC-1α activation. Meanwhile, while hypoxia-inducible factor 1-alpha inhibitor (HIF1an) hydroxylates AMP-activated kinase α1 subunit (AMPKα1) and inhibit its activity, miR-455-3p suppresses HIF1an to activate AMPKα1, which in turn induces mitochondria biogenesis via the HIF1an-AMPKα1-PGC1α regulatory cascade. Since the downstream target genes of miR-144-3p, miR-149-5p and miR-455-3p, including APP, PARP-2 and HIF1an, exhibit inverse correlation with PGC-1α, miR-144-3p, miR-149-5p and miR-455-3p can be considered as indirect stimulators of PGC-1α expression. **(b)** Schematic description of the binding sites for miR-29a-3p and miR-29c-3p in PGC-1α 3'-untranslated region (3'-UTR), for miR-34a-5p in SIRT1 3'-UTR, for miR-144-3p in APP 3'-UTR, for miR-149-5p in PARP-2 3'-UTR and for miR-455-3p in HIF1an 3'-UTR. The minimum free energy value of each hybrid is indicated. The seed recognition sites are denoted, and all nucleotides in these regions are highly conserved across species. **(c)** Conservation of the sequences of miR-29a-3p, miR-29c-3p, miR-34a-5p, miR-144-3p, miR-149-5p and miR-455-3p across various species.

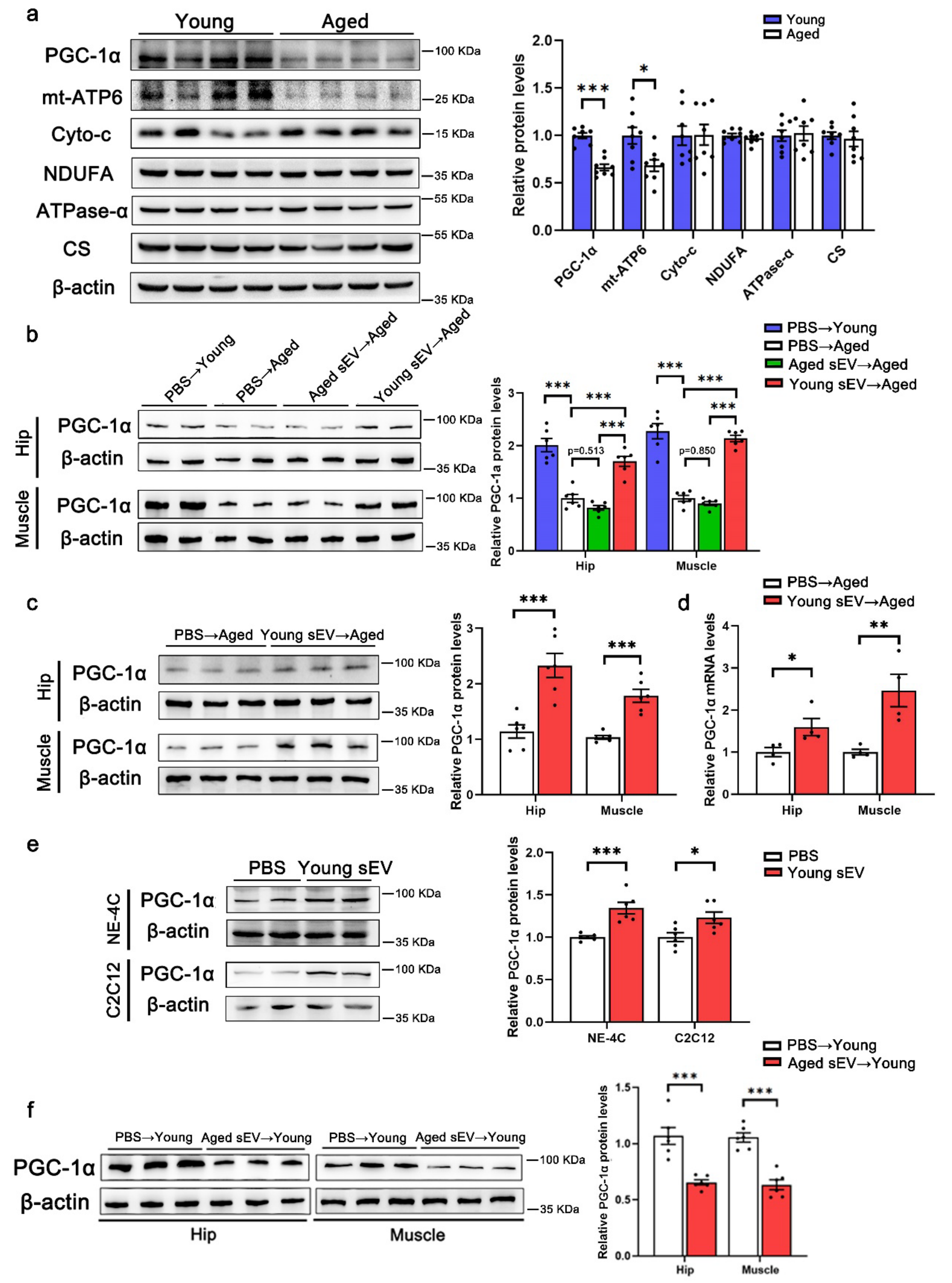

Supplementary Figure 18. Young and aged sEVs regulate PGC-1α expression *in vitro* and *in vivo*. (a) Western blot analysis of the protein levels of PGC-1α, mt-ATP6, Cyto-c (cytochrome c), NDUFA9 (NADH dehydrogenase (ubiquinone) 1α subcomplex, 9), ATPase-α and CS (citrate synthase) in the hippocampus and muscle of young and aged mice. Left panel: representative Western blots. Right panel: densitometric analysis (n = 8). **(b)** Western blot analysis of PGC-1α protein levels in the hippocampus and

muscle of aged mice injected with 200 μL of PBS, aged sEVs or young sEVs 7 times over 2 weeks. PBS-treated young mice serve as a control group. Left panel: representative Western blots. Right panel: densitometric analysis (n = 6). **(c)** Western blot analysis of PGC-1α protein levels in the hippocampus and muscle of aged mice injected with 200 μL of PBS or young human sEVs 7 times over 2 weeks. Left panel: representative Western blots. Right panel: densitometric analysis (n = 6). **(d)** Quantitative RT–PCR analysis of PGC-1α mRNA levels in the hippocampus and muscle of aged mice injected with 200 μL of PBS or young human sEVs 7 times over 2 weeks (n = 4). **(e)** Western blot analysis of PGC-1α protein levels in NE-4C cells and C2C12 cells incubated with 100 μL of PBS or young human sEVs for 24 hours. Left panel: representative Western blots. Right panel: densitometric analysis (n = 6). **(f)** Western blot analysis of PGC-1α protein levels in the hippocampus and muscle of young mice injected with 200 μL of PBS or aged mouse sEVs 7 times over 2 weeks. Left panel: representative Western blots. Right panel: densitometric analysis (n = 6). Significance was determined using two-sided Student's t-test in a, c, d, e and f and using one-way ANOVA followed by Dunnett's multiple comparison test in b. *P < 0.05, **P < 0.01 and ***P < 0.005.

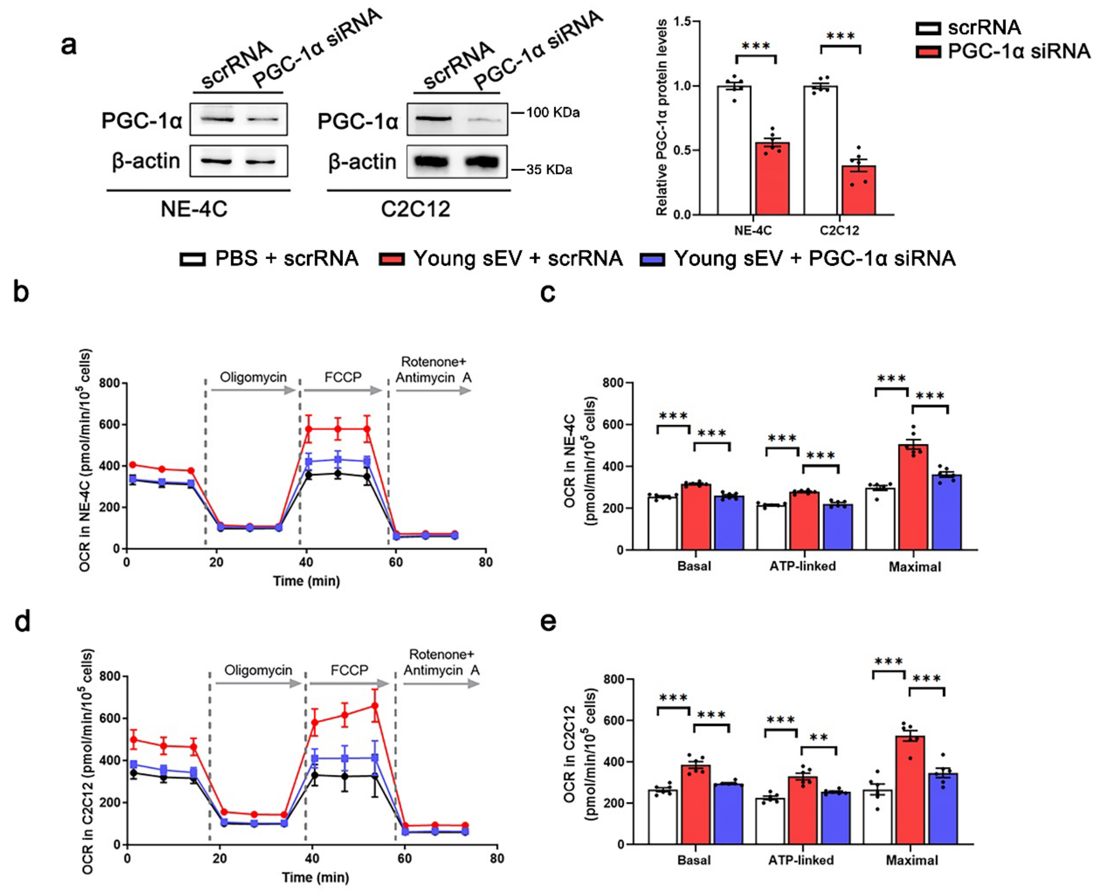

Supplementary Figure 19. PGC-1α siRNA blocks the beneficial effects of young sEVs on mitochondrial respiration. NE-4C or C2C12 cells (1×10^6 cells) were treated with PBS plus scrRNA, young sEVs plus scrRNA, or young sEVs plus PGC-1α siRNA for 24 hours, and then the cells were subjected to assessments of mitochondrial respiration. **(a)** Western blot analysis of PGC-1α protein levels in NE-4C and C2C12 cells after transfecting with scrRNA or PGC-1α siRNA. Left panel: representative Western blots. Right panel: densitometric analysis (n = 6). **(b-e)** Measurement of OCR in NE-4C and C2C12 cells after treatment with PBS plus scrRNA, young sEVs plus scrRNA, or young sEVs plus PGC-1α siRNA. After measurement of basal OCR, oligomycin, FCCP, and rotenone + antimycin A were sequentially added, and the alterations in OCR were recorded and normalized to cell number. Quantification of the basal OCR, ATP-coupled OCR and maximal OCR is shown (n = 6). Significance was determined using two-sided Student's t-test in a and using one-way ANOVA followed by Dunnett's multiple comparison test in c and e. *P < 0.05, **P < 0.01 and ***P < 0.005.

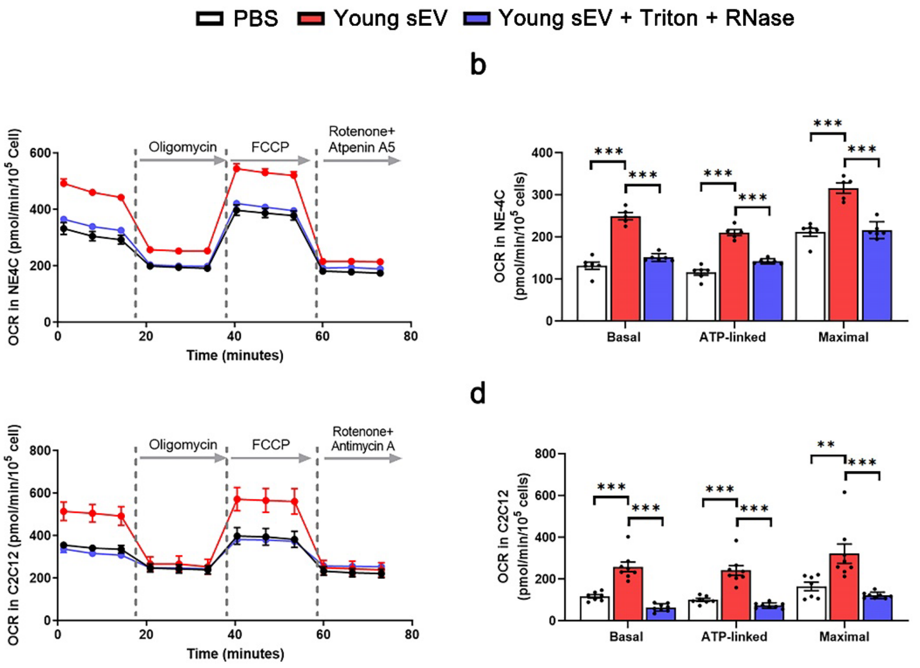

Supplementary Figure 20. Pre-treatment of young sEVs with Triton X-100 and RNase blocks the beneficial effects of young sEVs on mitochondrial respiration. Young sEVs were pre-treated with Triton X-100 and RNase, and then the resultant sEVs were incubated with NE-4C or C2C12 cells (1×10^6 cells) for 24 hours. NE-4C and C2C12 cells (1×10^6 cells) were also solely treated with PBS or young sEVs for 24 hours. After treatment, the cells were subjected to assessments of mitochondrial respiration. **(a-d)** Measurement of OCR in NE-4C and C2C12 cells. After measurement of basal OCR, oligomycin, FCCP, and rotenone + antimycin A were sequentially added, and the alterations in OCR were recorded and normalized to cell number. Quantification of the basal OCR, ATP-coupled OCR and maximal OCR is shown (NE-4C, n = 6 for PBS and Young sEV + Triton + RNase, n= 5 for Young sEV; C2C12, n =7 for PBS, n = 8 for Young sEV and Young sEV + Triton + RNase). Significance was determined using one-way ANOVA followed by Dunnett's multiple comparison test in b and d. *P < 0.05, **P < 0.01 and ***P < 0.005.

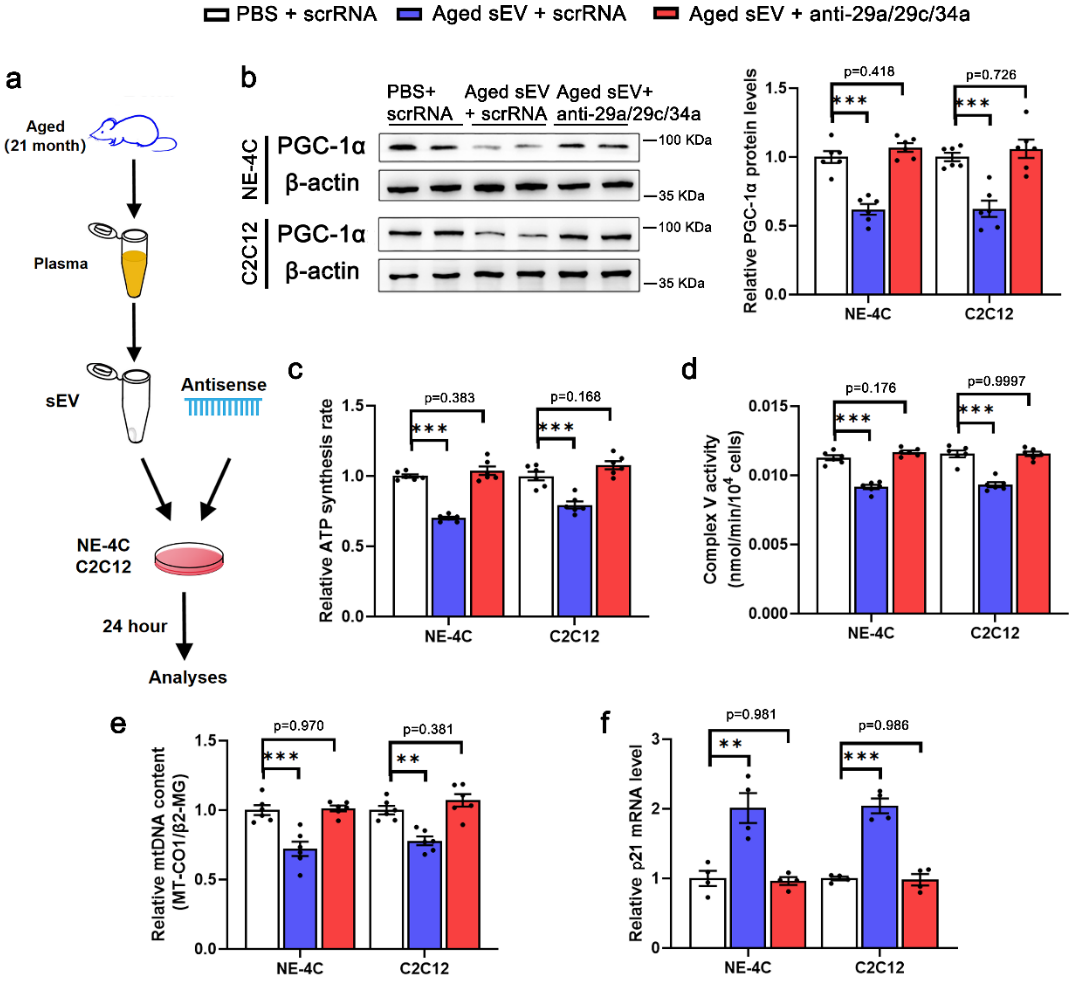

Supplementary Figure 21. Antisense oligonucleotides of miR-29a-3p, miR-29c-3p and miR-34a-5p rescue the detrimental effects of aged sEVs on mitochondrial metabolism and cell senescence. (a) Flow chart of the experimental design. NE-4C or C2C12 cells (1×10^6 cells) were treated with PBS plus scrRNA, aged sEVs plus scrRNA, or aged sEVs plus antisense oligonucleotides of miR-29a-3p, miR-29c-3p and miR-34a-5p (anti-miR-29a/29c/34a) for 24 hours, and then the cells were subjected to assessments of mitochondrial functional parameters and senescent phenotypes. **(b)** Western blot analysis of PGC-1α protein levels in NE-4C and C2C12 cells. Left panel: representative Western blots. Right panel: densitometric analysis (n = 6). **(c)** ATP synthesis rates in NE-4C and C2C12 cells (n = 6). **(d)** Mitochondrial complex V activity in NE-4C and C2C12 cells (n = 5). **(e)** Relative mtDNA content (MT-CO1/β2-MG) in NE-4C and C2C12 cells (n = 6). **(f)** Quantitative RT–PCR analysis of p21 mRNA levels in NE-4C and C2C12 cells (n = 4). Significance was determined using one-way ANOVA followed by Dunnett's multiple comparison test in b, c, d, e and f. *P < 0.05, **P < 0.01 and ***P < 0.005.

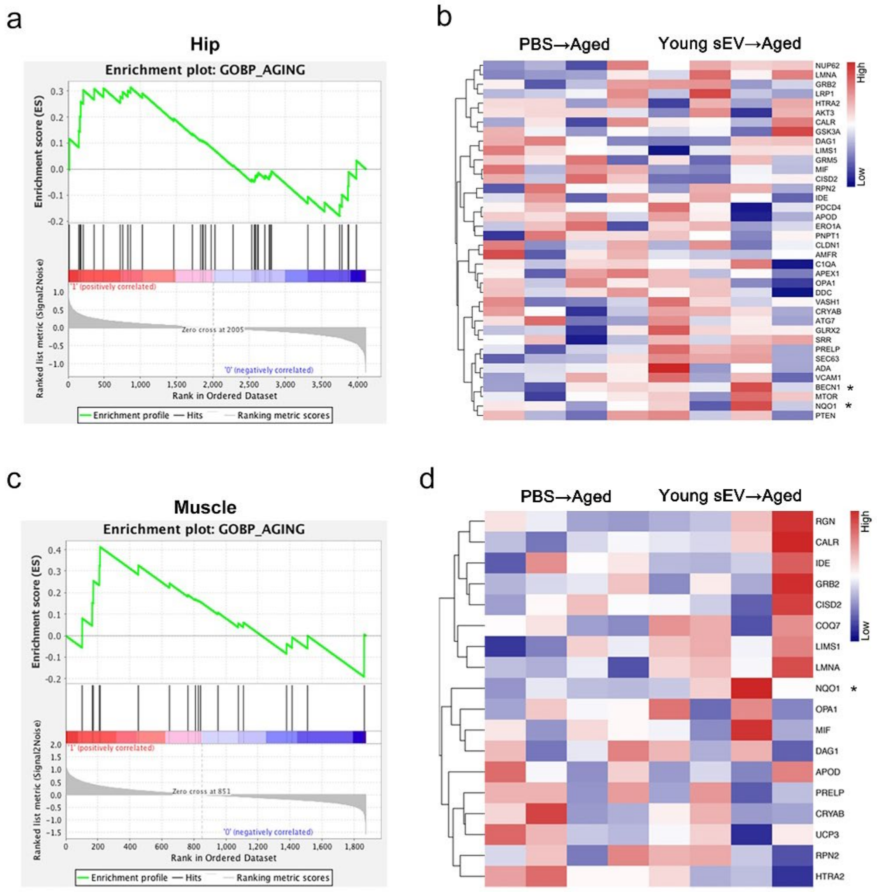

Supplementary Figure 22. The GO term GOBP_AGING (including GOBP_CELL AGING) is significantly upregulated and enriched in hippocampus and muscle. (a) Enrichment plot of the GO term GOBP_AGING showing the gene set that is upregulated in the hippocampus of “sEV→Aged” *vs.* “PBS→Aged” group with a Normalized Enrichment Score (NES) of 1.0678. **(b)** Heatmap showing the relative expression pattern of the 38 proteins in hippocampus involved in the GO term GOBP_AGING. **(c)** Enrichment plot of the GO term GOBP_AGING showing the gene set that is upregulated in the muscle of “sEV→Aged” *vs.* “PBS→Aged” group with a NES of 1.150. **(d)** Heatmap showing the relative expression pattern of the 18 proteins in muscle involved in the GO term GOBP_AGING. After young sEV treatment, beclin-1 (BECN1), an autophagy-regulating gene, was increased in aged hippocampus, and NAD(P)H dehydrogenase quinone 1 (NQO1), an antioxidant enzyme, was increased in aged hippocampus and muscle. Since BECN1 is decreased in human brains in an age-dependent fashion, leading to a reduction of autophagic activity and loss of cellular homeostasis during aging[66], modulation of BECN1 expression and restoration of BECN1-dependent autophagy by young sEVs can theoretically perform a neuroprotective effect against aging. Likewise, since the enzyme NQO1 plays a critical role in cellular antioxidant defense by effectively detoxifying quinones and, as a result, preventing the formation of ROS[67], age-associated decline in antioxidant potential and

accumulation of ROS in hippocampus and muscle can be rescued by young sEV-induced NQO1 upregulation.

Supplementary Tables

Supplementary Table 1. Assessment of frailty index scores in individual mouse (young sEV-treated aged mouse versus PBS-treated aged mouse) based on clinical signs of deterioration.

System and Parameter	PBS→Aged	PBS→Aged	PBS→Aged	PBS→Aged	PBS→Aged	PBS→Aged	PBS→Aged	PBS→Aged	PBS→Aged	PBS→Aged	sEV→Aged	sEV→Aged	sEV→Aged	sEV→Aged	sEV→Aged	sEV→Aged	sEV→Aged	sEV→Aged	sEV→Aged	sEV→Aged
Integument:																				
Alopecia	1	0.5	0	0.5	1	1	1	0.5	1	1	0.5	0.5	0	0.5	0.5	0.5	0.5	0.5	0	0.5
Loss of colour	1	1	1	1	1	1	1	0.5	1	0.5	1	0.5	0.5	0.5	0.5	0.5	0.5	0.5	0.5	0.5
Dermatitis	1	0	0	0	0.5	0	0.5	0	1	0	0	0		0	0	0	0	0	0	0
Loss of whiskers	0	0	0	0	0	0	0	0	1	0	0	0	0	0	0	0	0	0	0	0
Coat condition	1	1	0.5	0.5	0.5	1	1	0.5	1	0.5	0.5	0	1	0	0	0.5	0.5	0.5	0.5	0
Physical/Musculoskeletal:																				
Tumours	0.5	0	0.5	0.5	1	0	1	0	0	1	1	0	0	0	0	0	0	0	0	0
Distended abdomen	1	1	1	0.5	0	0.5	0	0.5	0.5	0.5	0	0	0.5	0	0	0	0	0	0.5	0
Kyphosis	0	0	0.5	0.5	0	0.5	0	0	0.5	0	0	0	0	0	0	0	0	0	0	0
Tail stiffening	0.5	0.5	1	0.5	1	1	0.5	1	0.5	0.5	0	0	0	0	0.5	0	0	0	0	0
Gait disorders	0.5	0.5	0.5	0	1	0.5	1	0	0	0.5	0	0	0	0	0	0.5	0	0.5	0	0
Tremor	1	1	0.5	0	0.5	1	1	0.5	1	0.5	0	0	0	0	0	0.5	0	0.5	0	0
Forelimb grip strength	0.5	0.5	0.5	0.5	0.5	1	0.5	0	0	0.5	0.5	0	0.5	0.5	0	0	0	0	0	0
Body condition score	1	1	1	0.5	0.5	1	0.5	0.5	1	0.5	0.5	0.5	0.5	0	0.5	0	0	0	0.5	0
Vestibulocochlear/Auditory:																				
Vestibular disturbance	0.5	0.5	1	0	0	0	0	0	0	0	0	0	0	0	0	0	0	0	0	0
Hearing loss	0.5	0.5	0	0	0	0	0	0	0	0.5	0.5	0	0	0	0	0	0	0	0	0
Ocular/Nasal:																				
Cataracts	0.5	0.5	0	0.5	0	0.5	0	0.5	0	0.5	0	0	0	0	0	0	0	0	0	0
Corneal opacity	0	0	0.5	0.5	0	0.5	0.5	0.5	0	0.5	0	0	0	0.5	0	0	0	0	0	0
Eye discharge /swelling	0	0	0	0	0	0.5	0.5	0	0.5	0.5	0	0	0	0	0.5	0	0	0	0	0
Microphthalmia	0	0	0	0	0	0	0	0	0	0	0	0	0	0	0	0	0	0	0	0
Vision loss	0	0	0	0	0	0	0	0	0	0	0	0	0	0	0	0	0	0		
Menace reflex	0	0	0	0	0	0	0	0	0	0	0	0	0	0	0	0	0	0	0	0
Nasal discharge	0	0	0	0	0	0	0	0	0	0	0	0	0	0	0	0	0	0	0	0
Digestive/Urogenital:																				
Malocclusions	0.5	0.5	0.5	0.5	0	0	0	0	0	0	0	0	0	0	0	0	0	0	0	0
Rectal prolapse	0	0	0	0	0	0	0	0	0	1	0	0	0	0	0	0	0	0	0	0
Vaginal/uterine/penile prolapse	0	0	0	0	0	0	0	0	0	1	0	0	0	0	0	0	0	0	0	0
Diarrhoea	0	0	0	0	0	0	0	0	0	0	0	0	0	0	0	0	0	0	0	0
Respiratory system:																				
breathing rate /depth	1	0.5	0.5	0.5	0.5	0.5	0.5	0.5	0.5	0.5	0	0	0.5	0	0	0.5	0	0.5	0.5	0.5
Discomfort:																				
Mouse Grimace Scale	0.5	0	0	0	0	0	0	0	0	0.5	0	0	0	0	0	0	0	0	0	0
Piloerection	0.5	0.5	0.5	0.5	0.5	0.5	0.5	0.5	0.5	0.5	0.5	0	0	0	0.5	0	0	0	0	0
Temperature score:	0.5	0.5	0.5	0.5	0.5	0.5	0.5	0.5	0.5	0.5	0.5	0.5	0.5	0.5	0.5	0.5	0.5	0.5	0.5	0.5
Body weight score:	1	1	0.5	1	0.5	0.5	1	0.5	0.5	0.5	0.5	0.5	0.5	0.5	0.5	0.5	0.5	0.5	0.5	0.5
Total Score	14.5	11.5	11	9	9.5	12	11.5	7	11	12.5	6	2.5	4.5	3	4	4	2.5	4	3.5	2.5
Clinical frailty index	0.4677419	0.3709677	0.3548387	0.2903226	0.3064516	0.3870968	0.3709677	0.2258065	0.3548387	0.4032258	0.1935484	0.0806452	0.1451613	0.0967742	0.1290323	0.1290323	0.0806452	0.1290323	0.1129032	0.0806452

Supplementary Table 2. The significantly differentially altered miRNAs in aged plasma compared with young plasma.

	Mean reads in aged mouse plasma	Mean reads in young mouse plasma	log_2FoldChange	P value*
miR-6953-3p	37.084	0	6.793	0.007
miR-7658-3p	31.648	0	6.565	0.008
miR-29a-5p	27.365	0	6.363	0.002
miR-7649-3p	25.445	0	6.245	0.037
miR-351-3p	25.022	0	6.221	0.033
miR-138-2-3p	23.094	0	6.105	0.042
miR-5107-5p	22.934	0	6.095	0.042
miR-3535	33.532	0.2479	6.093	0.007
miR-1968-5p	32.482	0.2070	6.050	0.006
miR-7036b-3p	21.918	0	6.030	0.044
miR-6395	19.561	0	5.874	0.012
miR-3473d	90.815	1.531	5.807	0.000
miR-466g	34.850	0.558	5.636	0.002
miR-702-3p	16.497	0	5.628	0.018
miR-330-3p	65.681	1.740	5.494	0.001
miR-1941-5p	20.336	0.351	5.361	0.045
miR-6948-3p	19.959	0.351	5.339	0.032
miR-338-5p	15.097	0.076	5.166	0.046
miR-490-5p	41.919	1.608	5.015	0.011
miR-101b-3p	88.077	3.061	4.770	0.000
miR-672-5p	50.058	3.516	3.783	0.032
miR-1843a-5p	77.388	5.792	3.623	0.002
miR-28a-5p	60.021	8.622	2.866	0.032
miR-29c-3p	67.263	18.066	2.020	0.042
miR-196b-5p	756.677	188.143	2.009	0.037
miR-3473e	44.614	10.971	1.965	0.045
miR-3473b	44.614	10.971	1.965	0.045
miR-23b-3p	9324.390	2576.480	1.856	0.000
miR-29a-3p	4997.068	1663.643	1.588	0.000
miR-34a-5p	4002.032	1420.359	1.494	0.009
miR-326-3p	1072.678	428.694	1.320	0.048
miR-27b-3p	14306.330	6851.343	1.063	0.006
miR-7a-5p	2412.016	4958.333	-1.040	0.031
mmu-let-7f-5p	17378.260	36186.470	-1.058	0.010
miR-149-5p	5365.526	11415.520	-1.089	0.007
mmu-let-7e-5p	448.391	1077.117	-1.264	0.022
miR-144-3p	427.786	1089.023	-1.349	0.011

miR-486a-3p	1009.263	2666.168	-1.401	0.031
miR-1983	127.551	368.438	-1.524	0.033
miR-615-3p	76.580	227.581	-1.575	0.040
miR-211-5p	122.288	461.185	-1.916	0.013
miR-199a-3p	1458.081	5705.585	-1.968	0.000
miR-135b-5p	11.570	55.409	-2.269	0.023
miR-8114	262.591	1358.905	-2.372	0.008
miR-127-3p	1197.295	6354.734	-2.408	0.000
miR-195a-5p	14.791	116.867	-2.969	0.020
miR-455-3p	3.252	27.151	-3.032	0.063
miR-379-5p	15.164	130.092	-3.099	0.016
miR-26b-3p	7.176	79.404	-3.465	0.024
miR-5106	12.500	146.600	-3.545	0.030
miR-296-5p	29.753	356.092	-3.578	0.004
miR-431-5p	2.665	35.310	-3.718	0.008
miR-8109	19.520	290.038	-3.888	0.000
miR-214-3p	14.468	249.799	-4.107	0.013
miR-5119	13.211	298.907	-4.499	0.002
miR-409-3p	3.366	120.373	-5.181	0.000
miR-5134-5p	0	10.807	-6.960	0.021
miR-30c-1-3p	0	11.563	-7.057	0.019
miR-329-3p	0	12.462	-7.182	0.001
miR-294-5p	0	20.105	-7.848	0.004
miR-298-5p	0	25.948	-8.218	0.000
miR-455-5p	0	28.552	-8.351	0.005
miR-668-3p	0	31.802	-8.507	0.003
miR-295-5p	0	34.546	-8.627	0.003
miR-293-5p	0	54.650	-9.288	0.001
miR-290a-3p	0	58.120	-9.376	0.001
miR-124-3p	0	66.138	-9.562	0.001
miR-291a-5p	0	180.362	-24.129	0.000
miR-291a-3p	0	117.063	-24.530	0.000
miR-290a-5p	0	160.729	-25.650	0.000
miR-292a-5p	0	272.972	-26.507	0.000
miR-294-3p	0	371.178	-26.926	0.000
miR-295-3p	0	615.431	-27.611	0.000
miR-293-3p	0	1000.708	-28.287	0.000
miR-292a-3p	0	1181.514	-28.514	0.000

* Significance was determined using two-sided Student's t-test.

Supplementary Table 3. Selection of age-associated circulating miRNAs (in plasma and serum) based on literature mining.

miRNA	Species	Source	Detection technique (RNA sequencing /qRT-PCR)	up/down	References
let-7a-5p	human and mouse	serum and plasma	RNA sequencing and qRT-PCR	down	[1-5]
let-7e-5p	human and mouse	plasma	qRT-PCR	down	[6-8]
let-7f-5p	human and mouse	serum and plasma	qRT-PCR	down	[1, 9-12]
let-7g-5p	human	whole blood and serum	RNA sequencing and qRT-PCR	up	[13-15]
let-7i-5p	human	serum and plasma	RNA sequencing and qRT-PCR	down	[16-19]
miR-1	human and mouse	serum and plasma	RNA sequencing and qRT-PCR	up	[20-24]
miR-106a	human and mouse	plasma	RNA sequencing and qRT-PCR	down	[25-33]
miR-106b	human and mouse	serum and plasma	RNA sequencing and qRT-PCR	down	[34-37]
miR-122-5p	human and mouse	plasma	RNA sequencing and qRT-PCR	up	[38-42]
miR-125b	human	serum and plasma	RNA sequencing and qRT-PCR	down	[43-47]
miR-125b-5p	human and mouse	serum and plasma	RNA sequencing and qRT-PCR	up	[18, 39, 40, 48, 49]
miR-126	human and mouse	serum and plasma	RNA sequencing and qRT-PCR	down	[20, 50-53]
miR-126-5p	human and mouse	serum and plasma	RNA sequencing and qRT-PCR	down	[54-56]
miR-129-5p	human and mouse	serum and plasma	RNA sequencing and qRT-PCR	up	[57-60]
miR-130b-5p	human and mouse	serum and plasma	RNA sequencing	up	[42, 61, 62]
miR-133a-3p	human and mouse	serum and plasma	RNA sequencing and qRT-PCR	up	[63-66]
miR-134-5p	human and mouse	plasma	qRT-PCR	up	[16, 67-72]
miR-138-5p	human and mouse	plasma	RNA sequencing	up	[42, 73-75]
miR-142-3p	human and mouse	serum and plasma	RNA sequencing and qRT-PCR	up	[2, 44, 65, 76-78]
miR-144-3p	human and mouse	serum and plasma	RNA sequencing and qRT-PCR	up	[9, 79-83]
miR-145-5p	human and mouse	serum and plasma	RNA sequencing and qRT-PCR	down	[84-87]
miR-148a-5p	human and mouse	plasma	RNA sequencing and qRT-PCR	up	[88-92]
miR-149-5p	human and mouse	plasma	RNA sequencing and qRT-PCR	down	[52, 93-96]
miR-150-5p	human and mouse	serum and plasma	qRT-PCR	up	[65, 97, 98]
miR-151a-3p	human and mouse	plasma	RNA sequencing and qRT-PCR	up	[19, 99-102]
miR-155-5p	human and mouse	plasma	qRT-PCR	up	[60, 103-105]
miR-15a	human and mouse	serum and plasma	qRT-PCR	up	[36, 106, 107]
miR-17	human	serum and plasma	RNA sequencing and qRT-PCR	down	[20, 52, 77, 108, 109]
miR-17-5p	human and mouse	serum and plasma	RNA sequencing and qRT-PCR	down	[7, 39, 79, 110-112]
miR-181a-5p	human and mouse	serum and plasma	qRT-PCR	up	[83, 90, 113]
miR-181b-5p	human and mouse	plasma	qRT-PCR	down	[114-116]
miR-183-5p	human and mouse	plasma	qRT-PCR	up	[16, 62, 117]
miR-185	human and mouse	serum and plasma	qRT-PCR	up	[21, 78, 118, 119]
miR-18a	human and mouse	serum and plasma	RNA sequencing and qRT-PCR	down	[20, 35, 52, 108]
miR-199a-3p	human and mouse	serum and plasma	RNA sequencing and qRT-PCR	down	[17, 25, 120, 121]

miRNA	Species	Source	Detection technique (RNA sequencing /qRT-PCR)	up/down	References
miR-19a-3p	human and mouse	serum and plasma	RNA sequencing and qRT-PCR	up	[13, 15, 122-125]
miR-19b-3p	human and mouse	plasma	RNA sequencing and qRT-PCR	up	[46, 82, 123]
miR-208a	human and mouse	serum and plasma	RNA sequencing and qRT-PCR	up	[20, 23, 52, 64, 126-128]
miR-208b	human and mouse	plasma	RNA sequencing and qRT-PCR	up	[22, 23, 127]
miR-20a-5p	human and mouse	serum and plasma	RNA sequencing and qRT-PCR	down	[35, 52, 108, 127, 129, 130]
miR-20b-5p	human and mouse	serum and plasma	RNA sequencing and qRT-PCR	down	[46, 131, 132]
miR-210	human	serum and plasma	RNA sequencing and qRT-PCR	up	[28, 36, 133]
miR-212	human	plasma	RNA sequencing and qRT-PCR	down	[18, 134, 135]
miR-214-3p	human and mouse	serum and plasma	RNA sequencing and qRT-PCR	up	[39, 136-138]
miR-21-5p	human	serum and plasma	RNA sequencing and qRT-PCR	up	[13, 40, 65, 139-143]
miR-217-5p	human and mouse	plasma	RNA sequencing	up	[42, 144, 145]
miR-22	human and mouse	plasma	RNA sequencing and qRT-PCR	up	[18, 73, 146]
miR-221-5p	human and mouse	serum and plasma	RNA sequencing and qRT-PCR	up	[23, 127, 147]
miR-22-3p	human and mouse	serum and plasma	RNA sequencing and qRT-PCR	down	[14, 102, 128, 148]
miR-23a-3p	human and mouse	serum and plasma	RNA sequencing and qRT-PCR	up	[4, 18, 40, 48, 149-151]
miR-23b-3p	human and mouse	serum and plasma	qRT-PCR	up	[12, 49, 152, 153]
miR-26b-5p	human and mouse	plasma	RNA sequencing and qRT-PCR	down	[11, 25, 46, 63, 154, 155]
miR-27a-3p	human and mouse	plasma	RNA sequencing and qRT-PCR	up	[13, 25, 155-159]
miR-27b-3p	human and mouse	plasma	RNA sequencing and qRT-PCR	up	[12, 157, 160, 161]
miR-29	human and mouse	serum and plasma	RNA sequencing and qRT-PCR	down	[20, 46, 162, 163]
miR-29a-3p	human and mouse	serum and plasma	RNA sequencing and qRT-PCR	up	[56, 82, 164-166]
miR-29b	human	plasma	RNA sequencing and qRT-PCR	down	[23, 34, 43, 46, 167-169]
miR-29b-3p	human and mouse	serum and plasma	qRT-PCR	up	[82, 170-172]
miR-29c-3p	human and mouse	serum and plasma	RNA sequencing and qRT-PCR	up	[46, 62, 136]
miR-30a-5p	human and mouse	serum and plasma	RNA sequencing and qRT-PCR	up	[39, 129, 173-177]
miR-31-5p	human	serum and plasma	RNA sequencing and qRT-PCR	down	[128, 148, 178-180]
miR-320b	human	serum and plasma	RNA sequencing and qRT-PCR	up	[168, 181-185]
miR-324-3p	human	serum and plasma	RNA sequencing and qRT-PCR	up	[62, 185, 186]
miR-326-3p	human and mouse	whole blood	RNA sequencing and qRT-PCR	up	[20, 57, 185, 187]
miR-328-3p	human	plasma	RNA sequencing	up	[14, 62, 103]
miR-330-3p	human and mouse	serum	qRT-PCR	up	[58, 188, 189]
miR-33a-5p	human and mouse	plasma	qRT-PCR	up	[190-192]
miR-33b	human and mouse	serum and plasma	qRT-PCR	up	[88, 193, 194]
miR-342-3p	human	serum and plasma	RNA sequencing and qRT-PCR	down	[36, 46, 89]
miR-345-5p	human	whole blood/serum	RNA sequencing and qRT-PCR	up	[195-197]
miR-34a-5p	human and mouse	serum and plasma	RNA sequencing and qRT-PCR	up	[6, 12, 105, 148, 198-202]
miR-378a-3p	human and mouse	plasma	RNA sequencing	down	[62, 191, 203]
miR-409-3p	human and mouse	serum and plasma	RNA sequencing and qRT-PCR	down	[36, 186, 204]

miRNA	Species	Source	Detection technique (RNA sequencing /qRT-PCR)	up/down	References
miR-423-3p	human and mouse	serum and plasma	qRT-PCR	up	[17, 148, 155]
miR-423-5p	human and mouse	serum and plasma	RNA sequencing and qRT-PCR	up	[36, 148, 155, 205]
miR-455-3p	human and mouse	serum and plasma	RNA sequencing and qRT-PCR	down	[30, 46, 59, 206-208]
miR-483-5p	human	serum and plasma	qRT-PCR	up	[44, 142, 184]
miR-486-5p	human	serum and plasma	RNA sequencing and qRT-PCR	up	[36, 132, 181, 204, 209]
miR-497-5p	human and mouse	serum and plasma	RNA sequencing and qRT-PCR	up	[46, 210, 211]
miR-499	human	plasma	qRT-PCR	down	[22, 23, 127, 212]
miR-501-3p	human	plasma	qRT-PCR	up	[11, 46, 213-215]
miR-7a-5p	human and mouse	serum and plasma	RNA sequencing and qRT-PCR	down	[216-218]
miR-9	human and mouse	serum and plasma	qRT-PCR	down	[43, 46, 167, 176, 219, 220]
miR-92a	human and mouse	serum and plasma	RNA sequencing and qRT-PCR	down	[34-36, 52, 108, 187, 221-224]
miR-92a-up	human and mouse	serum and plasma	RNA sequencing and qRT-PCR	up	[13, 42, 103, 181]

Supplementary Table 4. Antibody list.

Target	Isotype	Supplier Name	Cat#	Clone Name	Lot #	Dilution ratio
NDUFA	Mouse IgG_1 kappa	Thermo Fisher	459100	20C11B11B 11	TD2536591	1:1000 (WB)
ATPase-α	Mouse IgG2b, kappa	Thermo Fisher	459240	7H10BD4F9	TE2563181	1:1000 (WB)
CS	Mouse IgG_1 Kappa	Santa Cruz	sc-390693	G-3	H2714	1:1000 (WB)
Cyto-c	Mouse IgG2b, kappa	BD Biosciences	556433	7H8.2C12	1068185	1:1000 (WB)
PARP2	Mouse IgM kappa	Santa Cruz	Sc-393343	F-8	J0713	1:1000 (WB)
mt-ATP6	Rabbit IgG	Abcam	ab1924 23		GR 3198216-11	1:1000 (WB)
PGC-1α	Mouse IgG	Abcam	ab5448 1	EPR18289	GR3315850-1	1:1000 (WB)
P21	Rabbit IgG	Abcam	ab1882 24	EPR18021	GR3289181-4	1:1000 (WB)
MAP2	Rabbit IgG	Servicebio	GB 11128-2	P20357	AC 220511024	1:1000 (IF)
Desmin	Rabbit IgG	Proteintech	16520-1-AP	P17661	00099060	1:500 (IF)
SDHA	Mouse IgG	Abcam	ab1471 5	2E3GC12FB 2AE2	GR3263456-6	1:1000 (IF)
HIF1an	Rabbit IgG	Abcam	ab9230 4	EPR3659	GR77846-7	1:1000 (WB)
APP	Rabbit IgG	Abcam	ab3213 6	Y188	GR3287436-6	1:1000 (WB)
Alix	Rabbit IgG	Proteintech	12422-1-AP	Q8WUM4	00096216	1:2000 (WB)
TSG101	Rabbit IgG	Proteintech	14497-1-AP	Q99816	00093762	1:1000 (WB)
CD9	Mouse IgG_1 Kappa	Santa Cruz	sc-13118	C-4	G0121	1:1000 (WB)
CD63	Mouse IgG_1 Kappa	Santa Cruz	sc-5275	MX-49.129.5	C0320	1:1000 (WB)
Albumin	Rabbit IgG	Proteintech	16475-1-AP	P02768	00076243	1:2500 (WB)

Calnexin	Mouse IgG	Santa Cruz	Sc-23954	P35564	AF-18	1:1000 (WB)
β-actin	Rabbit IgG	Servicebio	GB11001	P60710	LS202310	1:1000 (WB)
**Secondary Antibodies**						
Goat anti-rabbit HRP-conjugated	Goat IgG	Santa Cruz	sc-2030		L1015	1:1000 (WB)
Goat anti-mouse HRP-conjugated	Goat IgG	Santa Cruz	sc-2005		B1616	1:1000 (WB)
BrdU	Mouse IgG1	Abcam	ab8152	IIB5	GR3340784-1	1:100 (IF)
DAPI	/	Santa Cruz	sc-24941		L1508	1:300 (IF)

Supplementary Table 5. Primer list.

Gene	Primer Sequence	Gene	Primer Sequence
	Primers for mRNAs		
P21 Forward	AGTCAGTTCCTTGTGGAGCC	β-actin Forward	GGCTGTATTCCCCTCCATCG
P21 Reverse	CATTAGCGCATCACAGTCGC	β-actin Reverse	CCAGTTGGTAACAATGCCATGT
PGC-1α Forward	AACAATGAGCCTGCGAACA		
PGC-1α Reverse	CATCAAATGAGGGCAATCC		
	Primers for mitochondrial DNAs		
MT-CO1 Forward	TTGGTCCCCTCCTCCAGC	MT-ND1 Forward	AGTCACCCTAGCCATCATTCTACT
MT-CO1 Reverse	CCAGTGCTAGCCGCAGGCA	MT-ND1 Reverse	GGAGTAATCAGAGGTGTTCTTGTGT
MT-CO3 Forward	AGGCATCACCCCGCTAAATC	D-loop Forward	CATCTGGTTCCTACTTCAGGG
MT-CO3 Reverse	GGTGAGCTCAGGTGATTGATACTC	D-loop Reverse	TGAGTGGTTAATAGGGTGATAGA
β2-MG Forward	GCGTGGGAGGAGCATCAGGG		
β2-MG Reverse	CTCATCACCACCCCGGGGACT		
	Primers for miRNAs		
mmu-miR-23a-3p Reverse	GTCGTATCCAGTGCAGGGTCCGAGGTATT CGCACTGGATACGACGGAAAT	mmu-miR-23a-3p Forward	GCGATCACATTGCCAGGG
mmu-miR-23b-3p Reverse	GTCGTATCCAGTGCAGGGTCCGAGGTATT CGCACTGGATACGACGTGGTA	mmu-miR-23b-3p Forward	CGATCACATTGCCAGGGAT
mmu-miR-27a-3p Reverse	GTCGTATCCAGTGCAGGGTCCGAGGTATT CGCACTGGATACGACGCGGAA	mmu-miR-27a-3p Forward	GCGCGTTCACAGTGGCTAAG
mmu-miR-27b-3p Reverse	GTCGTATCCAGTGCAGGGTCCGAGGTATT CGCACTGGATACGACGCAGAA	mmu-miR-27b-3p Forward	GCGCGTTCACAGTGGCTAAG
mmu-miR-29a-3p Reverse	GTCGTATCCAGTGCAGGGTCCGAGGTATT CGCACTGGATACGACTAACCG	mmu-miR-29a-3p Forward	CGCGTAGCACCATCTGAAAT
mmu-miR-29c-3p Reverse	GTCGTATCCAGTGCAGGGTCCGAGGTATT CGCACTGGATACGACTAACCG	mmu-miR-29c-3p Forward	CGCGTAGCACCATTTGAAAT

Gene	Primer Sequence	Gene	Primer Sequence
mmu-miR-34a-5p Reverse	GTCGTATCCAGTGCAGGGTCCGAGGTATT CGCACTGGATACGACACAACC	mmu-miR-34a-5p Forward	CGCGTGGCAGTGTCTTAGCT
mmu-miR-122-5p Reverse	GTCGTATCCAGTGCAGGGTCCGAGGTATT CGCACTGGATACGACCAAACA	mmu-miR-122-5p Forward	CGCGTGGAGTGTGACAATGG
mmu-miR-129-5p Reverse	GTCGTATCCAGTGCAGGGTCCGAGGTATT CGCACTGGATACGACGCAAGC	mmu-miR-129-5p Forward	CGCTTTTTGCGGTCTGG
mmu-miR-130b-5p Reverse	GTCGTATCCAGTGCAGGGTCCGAGGTATT CGCACTGGATACGACGTAGTG	mmu-miR-130b-5p Forward	CGCGACTCTTTCCCTGTTG
mmu-miR-134-5p Reverse	GTCGTATCCAGTGCAGGGTCCGAGGTATT CGCACTGGATACGACCCCCTC	mmu-miR-134-5p Forward	CGCGTGTGACTGGTTGACCA
mmu-miR-138-5p Reverse	GTCGTATCCAGTGCAGGGTCCGAGGTATT CGCACTGGATACGACCGGCCT	mmu-miR-138-5p Forward	GCGAGCTGGTGTTGTGAATC
mmu-miR-148a-5p Reverse	GTCGTATCCAGTGCAGGGTCCGAGGTATT CGCACTGGATACGACAGTCGG	mmu-miR-148a-5p Forward	GCGCGAAAGTTCTGAGACACT
mmu-miR-150-5p Reverse	GTCGTATCCAGTGCAGGGTCCGAGGTATT CGCACTGGATACGACCACTGG	mmu-miR-150-5p Forward	GCGTCTCCCAACCCTTGTA
mmu-miR-155-5p Reverse	GTCGTATCCAGTGCAGGGTCCGAGGTATT CGCACTGGATACGACACCCCT	mmu-miR-155-5p Forward	GCGCGTTAATGCTAATTGTGAT
mmu-miR-183-5p Reverse	GTCGTATCCAGTGCAGGGTCCGAGGTATT CGCACTGGATACGACAGTGAA	mmu-miR-183-5p Forward	CGCGTATGGCACTGGTAGAA
mmu-miR-192-5p Reverse	GTCGTATCCAGTGCAGGGTCCGAGGTATT CGCACTGGATACGACGGCTGT	mmu-miR-192-5p Forward	GCGCGCTGACCTATGAATTG
mmu-miR-217-5p Reverse	GTCGTATCCAGTGCAGGGTCCGAGGTATT CGCACTGGATACGACTCCAAT	mmu-miR-217-5p Forward	CGCGTACTGCATCAGGAACTG
mmu-miR-221-5p Reverse	GTCGTATCCAGTGCAGGGTCCGAGGTATT CGCACTGGATACGACAAATCT	mmu-miR-221-5p Forward	CGCGACCTGGCATACAATGT

Gene	Primer Sequence	Gene	Primer Sequence
mmu-miR-10a-5p	GTCGTATCCAGTGCAGGGTCCGAGGTATT	mmu-miR-10a-5p	CGCGTACCCTGTAGATCCGAA
Reverse	CGCACTGGATACGACCACAAA	Forward	
mmu-miR-7-5p	GTCGTATCCAGTGCAGGGTCCGAGGTATT	mmu-miR-7-5p	CGCGTGGAAGACTAGTGATTTT
Reverse	CGCACTGGATACGACAACAAC	Forward	
mmu-miR-17-5p	GTCGTATCCAGTGCAGGGTCCGAGGTATT	mmu-miR-17-5p	GCGCAAAGTGCTTACAGTGC
Reverse	CGCACTGGATACGACCTACCT	Forward	
mmu-miR-20a	GTCGTATCCAGTGCAGGGTCCGAGGTATT	mmu-miR-20a	GCGCGTAAAGTGCTTATAGTGC
Reverse	CGCACTGGATACGACCTACCT	Forward	
mmu-miR-92a	GTCGTATCCAGTGCAGGGTCCGAGGTATT	mmu-miR-92a	CGCGTATTGCACTTGTCCC
Reverse	CGCACTGGATACGACCAGGCC	Forward	
mmu-miR-106a	GTCGTATCCAGTGCAGGGTCCGAGGTATT	mmu-miR-106a	GCGCAAAGTGCTAACAGTGC
Reverse	CGCACTGGATACGACCTACCT	Forward	
mmu-miR-126-5p	GTCGTATCCAGTGCAGGGTCCGAGGTATT	mmu-miR-126-5p	GCGCGCATTATTACTTTTGG
Reverse	CGCACTGGATACGACCGCGTA	Forward	
mmu-miR-144-3p	GTCGTATCCAGTGCAGGGTCCGAGGTATT	mmu-miR-144-3p	GCGCGCGTACAGTATAGATGA
Reverse	CGCACTGGATACGACAGTACA	Forward	
mmu-miR-145-5p	GTCGTATCCAGTGCAGGGTCCGAGGTATT	mmu-miR-145-5p	CGGTCCAGTTTTCCCAGGA
Reverse	CGCACTGGATACGACAGGGAT	Forward	
mmu-miR-149-5p	GTCGTATCCAGTGCAGGGTCCGAGGTATT	mmu-miR-149-5p	CGTCTGGCTCCGTGTCTTC
Reverse	CGCACTGGATACGACGGGAGT	Forward	
mmu-miR-378a-3p	GTCGTATCCAGTGCAGGGTCCGAGGTATT	mmu-miR-378a-3p	CGCGACTGGACTTGGAGTCA
Reverse	CGCACTGGATACGACGCCTTC	Forward	
mmu-miR-455-3p	GTCGTATCCAGTGCAGGGTCCGAGGTATT	mmu-miR-455-3p	CGGCAGTCCATGGGCAT
Reverse	CGCACTGGATACGACGTGTAT	Forward	
mmu-let-7a-5p	GTCGTATCCAGTGCAGGGTCCGAGGTATT	mmu-let-7a-5p	GCGCGTGAGGTAGTAGGTTGT
Reverse	CGCACTGGATACGACAACTAT	Forward	

Gene	Primer Sequence	Gene	Primer Sequence
mmu-let-7f-5p	GTCGTATCCAGTGCAGGGTCCGAGGTATT	mmu-let-7f-5p	CGCGCGTGAGGTAGTAGATTGT
Reverse	CGCACTGGATACGACAACTAT	Forward	
mmu-miR-16-1-3p	GTCGTATCCAGTGCAGGGTCCGAGGTATT	mmu-miR-16-1-3p	CGCGCCAGTATTGACTGTGC
Reverse	CGCACTGGATACGACTCAGCA	Forward	
hsa-miR-23a-3p	GTCGTATCCAGTGCAGGGTCCGAGGTATT	hsa-miR-23a-3p	GCGATCACATTGCCAGGG
Reverse	CGCACTGGATACGACGGAAAT	Forward	
hsa-miR-23b-3p	GTCGTATCCAGTGCAGGGTCCGAGGTATT	hsa-miR-23b-3p	CGATCACATTGCCAGGGAT
Reverse	CGCACTGGATACGACGTGGTA	Forward	
hsa-miR-27a-3p	GTCGTATCCAGTGCAGGGTCCGAGGTATT	hsa-miR-27a-3p	GCGCGTTCACAGTGGCTAAG
Reverse	CGCACTGGATACGACGCGGAA	Forward	
hsa-miR-27b-3p	GTCGTATCCAGTGCAGGGTCCGAGGTATT	hsa-miR-27b-3p	GCGCGTTCACAGTGGCTAAG
Reverse	CGCACTGGATACGACGCAGAA	Forward	
hsa-miR-29a-3p	GTCGTATCCAGTGCAGGGTCCGAGGTATT	hsa-miR-29a-3p	CGCGTAGCACCATCTGAAAT
Reverse	CGCACTGGATACGACTAACCG	Forward	
hsa-miR-29c-3p	GTCGTATCCAGTGCAGGGTCCGAGGTATT	hsa-miR-29c-3p	CGCGTAGCACCATTTGAAAT
Reverse	CGCACTGGATACGACTAACCG	Forward	
hsa-miR-34a-5p	GTCGTATCCAGTGCAGGGTCCGAGGTATT	hsa-miR-34a-5p	CGCGTGGCAGTGTCTTAGCT
Reverse	CGCACTGGATACGACACAACC	Forward	
hsa-miR-130b-5p	GTCGTATCCAGTGCAGGGTCCGAGGTATT	hsa-miR-130b-5p	CGCGACTCTTTCCCTGTTG
Reverse	CGCACTGGATACGACGTAGTG	Forward	
hsa-miR-150-5p	GTCGTATCCAGTGCAGGGTCCGAGGTATT	hsa-miR-150-5p	GCGTCTCCCAACCCTTGTA
Reverse	CGCACTGGATACGACCACTGG	Forward	
hsa-miR-221-5p	GTCGTATCCAGTGCAGGGTCCGAGGTATT	hsa-miR-221-5p	CGCGACCTGGCATACAATGT
Reverse	CGCACTGGATACGACAAATCT	Forward	
hsa-miR-17-5p	GTCGTATCCAGTGCAGGGTCCGAGGTATT	hsa-miR-17-5p	GCGCAAAGTGCTTACAGTGC
Reverse	CGCACTGGATACGACCTACCT	Forward	

Gene	Primer Sequence	Gene	Primer Sequence
hsa-miR-126-5p	GTCGTATCCAGTGCAGGGTCCGAGGTATT	hsa-miR126-5p	GCGCGCATTATTACTTTTGG
Reverse	CGCACTGGATACGACCGCGTA	Forward	
hsa-miR-144-3p	GTCGTATCCAGTGCAGGGTCCGAGGTATT	hsa-miR144-3p	GCGCGCGTACAGTATAGATGA
Reverse	CGCACTGGATACGACAGTACA	Forward	
hsa-miR-149-5p	GTCGTATCCAGTGCAGGGTCCGAGGTATT	hsa-miR-149-5p	CGTCTGGCTCCGTGTCTTC
Reverse	CGCACTGGATACGACGGGAGT	Forward	
hsa-miR-378a-3p	GTCGTATCCAGTGCAGGGTCCGAGGTATT	hsa-miR-378a-3p	CGCGACTGGACTTGGAGTCA
Reverse	CGCACTGGATACGACGCCTTC	Forward	
hsa-miR-455-3p	GTCGTATCCAGTGCAGGGTCCGAGGTATT	hsa-miR-455-3p	CGGCAGTCCATGGGCAT
Reverse	CGCACTGGATACGACGTGTAT	Forward	
hsa-let-7a-5p	GTCGTATCCAGTGCAGGGTCCGAGGTATT	hsa-let-7a-5p	GCGCGTGAGGTAGTAGGTTGT
Reverse	CGCACTGGATACGACAACTAT	Forward	
hsa-let-7f-5p	GTCGTATCCAGTGCAGGGTCCGAGGTATT	hsa-let-7f-5p	CGCGCGTGAGGTAGTAGATTGT
Reverse	CGCACTGGATACGACAACTAT	Forward	
hsa-miR-16-1-3p	GTCGTATCCAGTGCAGGGTCCGAGGTATT	hsa-miR-16-1-3p	CGCGCCAGTATTGACTGTGC
Reverse	CGCACTGGATACGACTCAGCA	Forward	
Primers for pre-miRNAs			
mmu-pre-miR-144	GTCGTATCCAGTGCAGGGTCCGAGGTATT	mmu-pre-miR-144	GTGATGAGACACTACAGTATAGATG
Reverse	CGCACTGGATACGACGACTAG	Forward	ATGTA
mmu-pre-miR-149	GTCGTATCCAGTGCAGGGTCCGAGGTATT	mmu-pre-miR-149	AGGGAGGGACGGGGGC
Reverse	CGCACTGGATACGACAGCACC	Forward	
mmu-pre-miR-455	GTCGTATCCAGTGCAGGGTCCGAGGTATT	mmu-pre-miR-455	CAGTCCACGGGCATATACACTT
Reverse	CGCACTGGATACGACTGAGGC	Forward	

Supplementary Table 6. Sequences of synthetic miRNA mimics and antisenses.

miRNA name	miRNA mimics	miRNA antisense
mmu-miR-29a-3p	Forward: UAGCACCAUCUGAAAUCGGUUA<br>Reverse: ACCGAUUUCAGAUGGUGCUAUU	UAACCGAUUUCAGAUGGUGCUA
mmu-miR-29c-3p	Forward: UAGCACCAUUUGAAAUCGGUUA<br>Reverse: ACCGAUUUCAAAUGGUGCUAUU	UAACCGAUUUCAAAUGGUGCUA
mmu-miR-34a-5p	Forward: UGGCAGUGUCUUAGCUGGUUGU<br>Reverse: AACCAGCUAAGACACUGCCAUU	ACAACCAGCUAAGACACUGCCA
mmu-miR-144-3p	Forward: UACAGUAUAGAUGAUGUACU<br>Reverse: UACAUCAUCUAUACUGUAUU	AGUACAUCAUCUAUACUGUA
mmu-miR-149-5p	Forward: UCUGGCUCCGUGUCUUCACUCCC<br>Reverse: GAGUGAAGACACGGAGCCAGAUU	GGGAGUGAAGACACGGAGCCAGA
mmu-miR-455-3p	Forward: GCAGUCCAUGGGCAUAUACAC<br>Reverse: GUAUAUGCCCAUGGACUGCUU	GUGUAUAUGCCCAUGGACUGC

References

1. Fehlmann, T., et al., *Common diseases alter the physiological age-related blood microRNA profile.* Nature Communications, 2020. **11**(1).
2. Lai, C.-Y., et al., *Modulated expression of human peripheral blood microRNAs from infancy to adulthood and its role in aging.* Aging Cell, 2014. **13**(4): p. 679-689.
3. Guo, J., et al., *Dicer1 downregulation by multiple myeloma cells promotes the senescence and tumor-supporting capacity and decreases the differentiation potential of mesenchymal stem cells.* Cell Death & Disease, 2018. **9**.
4. Tzaridis, T., et al., *Extracellular Vesicle Separation Techniques Impact Results from Human Blood Samples: Considerations for Diagnostic Applications.* International Journal of Molecular Sciences, 2021. **22**(17).
5. Ragusa, M., et al., *Molecular Crosstalking among Noncoding RNAs: A New Network Layer of Genome Regulation in Cancer.* International Journal of Genomics, 2017. **2017**.
6. Markopoulos, G.S., et al., *Senescence-associated microRNAs target cell cycle regulatory genes in normal human lung fibroblasts.* Experimental Gerontology, 2017. **96**: p. 110-122.
7. Daimiel, L., et al., *Impact of Phenol-Enriched Virgin Olive Oils on the Postprandial Levels of Circulating microRNAs Related to Cardiovascular Disease.* Molecular Nutrition & Food Research, 2020. **64**(15).
8. Yubero-Serrano, E.M., et al., *Mediterranean diet and endothelial function in patients with coronary heart disease: An analysis of the CORDIOPREV randomized controlled trial.* Plos Medicine, 2020. **17**(9).
9. Pienimaeki-Roemer, A., et al., *Transcriptomic profiling of platelet senescence and platelet extracellular vesicles.* Transfusion, 2017. **57**(1): p. 144-156.
10. Li, Z.-H., et al., *Let-7f-5p suppresses Th17 differentiation via targeting STAT3 in multiple sclerosis.* Aging-Us, 2019. **11**(13): p. 4463-4477.

11. Li, H., et al., *Identification of molecular alterations in leukocytes from gene expression profiles of peripheral whole blood of Alzheimer's disease.* Scientific Reports, 2017. **7**.
12. Luk, H.-Y., et al., *Impacts of Green Tea on Joint and Skeletal Muscle Health: Prospects of Translational Nutrition.* Antioxidants, 2020. **9**(11).
13. Raut, J.R., et al., *A microRNA panel compared to environmental and polygenic scores for colorectal cancer risk prediction.* Nature Communications, 2021. **12**(1).
14. Weilner, S., et al., *Differentially circulating miRNAs after recent osteoporotic fractures can influence osteogenic differentiation.* Bone, 2015. **79**: p. 43-51.
15. Nevola, K.T., et al., *Pharmacogenomic Effects of beta-Blocker Use on Femoral Neck Bone Mineral Density.* Journal of the Endocrine Society, 2021. **5**(8).
16. Tong, K.-L., et al., *Circulating MicroRNAs in Young Patients with Acute Coronary Syndrome.* International Journal of Molecular Sciences, 2018. **19**(5).
17. Bruno, N., et al., *MicroRNAs relate to early worsening of renal function in patients with acute heart failure.* International Journal of Cardiology, 2016. **203**: p. 564-569.
18. Dong, X., D. Zheng, and J. Nao, *Circulating Exosome microRNAs as Diagnostic Biomarkers of Dementia.* Frontiers in Aging Neuroscience, 2020. **12**.
19. Gamez-Valero, A., et al., *Exploratory study on microRNA profiles from plasma-derived extracellular vesicles in Alzheimer's disease and dementia with Lewy bodies.* Translational Neurodegeneration, 2019. **8**(1).
20. Danaii, S., et al., *The Association between Inflammatory Cytokines and miRNAs with Slow Coronary Flow Phenomenon.* Iranian Journal of Allergy Asthma and Immunology, 2020. **19**(1): p. 56-64.
21. Rusanova, I., et al., *Involvement of plasma miRNAs, muscle miRNAs and mitochondrial miRNAs in the pathophysiology of frailty.* Experimental Gerontology, 2019. **124**.
22. Zhang, T., et al., *Improved knee extensor strength with resistance training associates with muscle specific miRNAs in older adults.* Experimental Gerontology, 2015. **62**: p. 7-13.
23. Pellegrini, L., et al., *MicroRNAs in Cancer Treatment-Induced Cardiotoxicity.* Cancers, 2020. **12**(3).
24. Lv, X., et al., *Exosomal long non-coding RNA LINC00662 promotes non-small cell lung cancer progression by miR-320d/E2F1 axis.* Aging-Us, 2021. **13**(4): p. 6010-6024.
25. Ovchinnikova, T.E.E., et al., *Signature of circulating microRNAs in patients with acute heart failure.* European Journal of Heart Failure, 2015. **17**: p. 119-119.
26. Tzaridis, T., et al., *Analysis of Serum miRNA in Glioblastoma Patients: CD44-Based Enrichment of Extracellular Vesicles Enhances Specificity for the Prognostic Signature.* International Journal of Molecular Sciences, 2020. **21**(19).
27. Lauretti, E., K. Dabrowski, and D. Pratico, *The neurobiology of non-coding RNAs and Alzheimer's disease pathogenesis: Pathways, mechanisms and translational opportunities.* Ageing Research Reviews, 2021. **71**.
28. Mengel-From, J., et al., *Circulating, Cell-Free Micro-RNA Profiles Reflect Discordant Development of Dementia in Monozygotic Twins.* Journal of Alzheimers Disease, 2018. **63**(2): p. 591-601.
29. Harries, L.W., *MicroRNAs as Mediators of the Ageing Process.* Genes, 2014. **5**(3): p. 656-670.

30. Xu, M., et al., *MicroRNAs Regulate Thymic Epithelium in Age-Related Thymic Involution via Down- or Upregulation of Transcription Factors.* Journal of Immunology Research, 2017. **2017**.
31. Abuelezz, N.Z., et al., *MicroRNAs as Potential Orchestrators of Alzheimer's Disease-Related Pathologies: Insights on Current Status and Future Possibilities.* Frontiers in Aging Neuroscience, 2021. **13**.
32. Watanabe, K., et al., *Functional similarities of microRNAs across different types of tissue stem cells in aging.* Inflammation and Regeneration, 2018. **38**.
33. Yamamoto, T., et al., *A Challenge to Aging Society by microRNA in Extracellular Vesicles: microRNA in Extracellular Vesicles as Promising Biomarkers and Novel Therapeutic Targets in Multiple Myeloma.* Journal of Clinical Medicine, 2018. **7**(3).
34. Zhang, H., et al., *Investigation of MicroRNA Expression in Human Serum During the Aging Process.* Journals of Gerontology Series a-Biological Sciences and Medical Sciences, 2015. **70**(1): p. 102-109.
35. Luo, X., et al., *Identification and Evaluation of Plasma MicroRNAs for Early Detection of Colorectal Cancer.* Plos One, 2013. **8**(5).
36. Hatse, S., et al., *Circulating MicroRNAs as Easy-to-Measure Aging Biomarkers in Older Breast Cancer Patients: Correlation with Chronological Age but Not with Fitness/Frailty Status.* Plos One, 2014. **9**(10).
37. Menard, C., et al., *miR-106b suppresses pathological retinal angiogenesis.* Aging-Us, 2020. **12**(24): p. 24836-24852.
38. Mandourah, A.Y., et al., *Circulating microRNAs as potential diagnostic biomarkers for osteoporosis.* Scientific Reports, 2018. **8**.
39. Luis, A., et al., *Circulating miRNAs Associated With ER Stress and Organ Damage in a Preclinical Model of Trauma Hemorrhagic Shock.* Frontiers in Medicine, 2020. **7**.
40. He, N., et al., *Increasing Fracture Risk Associates With Plasma Circulating MicroRNAs in Aging People's Sarcopenia.* Frontiers in Physiology, 2021. **12**.
41. Li, D.-B., et al., *Plasma Exosomal miRNA-122-5p and miR-300-3p as Potential Markers for Transient Ischaemic Attack in Rats.* Frontiers in Aging Neuroscience, 2018. **10**.
42. Wang, K., et al., *Melatonin Enhances the Therapeutic Effect of Plasma Exosomes Against Cerebral Ischemia-Induced Pyroptosis Through the TLR4/NF-kappa B Pathway.* Frontiers in Neuroscience, 2020. **14**.
43. Maldonado-Lasuncion, I., et al., *Aging-Related Changes in Cognition and Cortical Integrity are Associated With Serum Expression of Candidate MicroRNAs for Alzheimer Disease.* Cerebral Cortex, 2019. **29**(10): p. 4426-4437.
44. Al-Rawaf, H.A., A.H. Alghadir, and S.A. Gabr, *Molecular Changes in Circulating microRNAs' Expression and Oxidative Stress in Adults with Mild Cognitive Impairment: A Biochemical and Molecular Study.* Clinical Interventions in Aging, 2021. **16**: p. 57-70.
45. Cheng, N.-L., et al., *MicroRNA-125b modulates inflammatory chemokine CCL4 expression in immune cells and its reduction causes CCL4 increase with age.* Aging Cell, 2015. **14**(2): p. 200-208.
46. Zhao, Y., et al., *The Potential Markers of Circulating microRNAs and long non-coding RNAs in Alzheimer's Disease.* Aging and Disease, 2019. **10**(6): p. 1293-1301.

47. Deng, Q., et al., *Exosomal long non-coding RNA MSTRG.292666.16 is associated with osimertinib (AZD9291) resistance in non-small cell lung cancer.* Aging-Us, 2020. **12**(9): p. 8001-8015.
48. Verma, S., et al., *Signature transcriptome analysis of stage specific atherosclerotic plaques of patients.* Bmc Medical Genomics, 2022. **15**(1).
49. Ramzan, F., et al., *Comprehensive Profiling of the Circulatory miRNAome Response to a High Protein Diet in Elderly Men: A Potential Role in Inflammatory Response Modulation.* Molecular Nutrition & Food Research, 2019. **63**(8).
50. Behbahanipour, M., et al., *Expression Profiling of Blood microRNAs 885, 361, and 17 in the Patients with the Parkinson's disease: Integrating Interaction Data to Uncover the Possible Triggering Age-Related Mechanisms.* Scientific Reports, 2019. **9**.
51. Sun, J., et al., *Endothelial progenitor cell-derived exosomes, loaded with miR-126, promoted deep vein thrombosis resolution and recanalization.* Stem Cell Research & Therapy, 2018. **9**.
52. Zhang, Y., et al., *MicroRNAs or Long Noncoding RNAs in Diagnosis and Prognosis of Coronary Artery Disease.* Aging and Disease, 2019. **10**(2): p. 353-366.
53. Banerjee, J., et al., *Senescence-associated miR-34a and miR-126 in middle-aged Indians with type 2 diabetes.* Clinical and Experimental Medicine, 2020. **20**(1): p. 149-158.
54. Lee, B.-R., et al., *Effect of young exosomes injected in aged mice.* International Journal of Nanomedicine, 2018. **13**: p. 5335-5345.
55. Li, H.-y., et al., *Plasma MicroRNA-126-5p is Associated with the Complexity and Severity of Coronary Artery Disease in Patients with Stable Angina Pectoris.* Cellular Physiology and Biochemistry, 2016. **39**(3): p. 837-846.
56. Chi, J., et al., *Integrated Analysis and Identification of Novel Biomarkers in Parkinson's Disease.* Frontiers in Aging Neuroscience, 2018. **10**.
57. Dogan, S., et al., *Roles of adiponectin and leptin signaling-related microRNAs in the preventive effects of calorie restriction in mammary tumor development.* Applied Physiology Nutrition and Metabolism, 2021. **46**(8): p. 866-876.
58. Catanesi, M., et al., *MicroRNAs Dysregulation and Mitochondrial Dysfunction in Neurodegenerative Diseases.* International Journal of Molecular Sciences, 2020. **21**(17).
59. Lee, J. and H. Kang, *Role of MicroRNAs and Long Non-Coding RNAs in Sarcopenia.* Cells, 2022. **11**(2).
60. Rajman, M., et al., *A microRNA-129-5p/Rbfox crosstalk coordinates homeostatic downscaling of excitatory synapses.* Embo Journal, 2017. **36**(12): p. 1770-1787.
61. Huan, T., et al., *Age-associated microRNA Expression in Human Peripheral Blood is Associated With All-cause Mortality and Age-related Traits.* Circulation, 2016. **134**.
62. Sitlinger, A., et al., *Physiological Fitness and the Pathophysiology of Chronic Lymphocytic Leukemia (CLL).* Cells, 2021. **10**(5).
63. Ren, S., et al., *Circular RNAs: Promising Molecular Biomarkers of Human Aging-Related Diseases via Functioning as an miRNA Sponge.* Molecular Therapy-Methods & Clinical Development, 2020. **18**: p. 215-229.
64. Yu, W., et al., *Differential expression profiles of miRNA in the serum of sarcopenic rats.* Biochemistry and Biophysics Reports, 2022. **30**.

65. Kunadian, V., et al., *Study to Improve Cardiovascular Outcomes in high-risk older patieNts (ICON1) with acute coronary syndrome: study design and protocol of a prospective observational study.* Bmj Open, 2016. **6**(8).
66. Su, M.-T., et al., *LILRB4 promotes tumor metastasis by regulating MDSCs and inhibiting miR-1 family miRNAs.* Oncoimmunology, 2022. **11**(1).
67. Sheinerman, K., et al., *Age- and sex-dependent changes in levels of circulating brain-enriched microRNAs during normal aging.* Aging-Us, 2018. **10**(10): p. 3017-3041.
68. Dhahbi, J.M., et al., *Deep sequencing identifies circulating mouse miRNAs that are functionally implicated in manifestations of aging and responsive to calorie restriction.* Aging-Us, 2013. **5**(2): p. 130-141.
69. Dal Lin, C., et al., *Rapid changes of miRNAs-20,-30,-410,-515,-134, and-183 and telomerase with psychological activity: A one year study on the relaxation response and epistemological considerations.* Journal of Traditional and Complementary Medicine, 2021. **11**(5): p. 409-418.
70. Kou, X. and N. Chen, *Resveratrol as a Natural Autophagy Regulator for Prevention and Treatment of Alzheimer's Disease.* Nutrients, 2017. **9**(9).
71. Mohammed, C.P.D., et al., *MicroRNAs in brain aging.* Mechanisms of Ageing and Development, 2017. **168**: p. 3-9.
72. Bronze-da-Rocha, E., *MicroRNAs Expression Profiles in Cardiovascular Diseases.* Biomed Research International, 2014. **2014**.
73. Garza-Manero, S., et al., *Identification of age- and disease-related alterations in circulating miRNAs in a mouse model of Alzheimer's disease.* Frontiers in Cellular Neuroscience, 2015. **9**.
74. Zhao, B., et al., *Transplantation of bone marrow-derived mesenchymal stem cells with silencing of microRNA-138 relieves pelvic organ prolapse through the FBLN5/IL-1 beta/elastin pathway.* Aging-Us, 2021. **13**(2): p. 3045-3059.
75. Amakiri, N., et al., *Amyloid Beta and MicroRNAs in Alzheimer's Disease.* Frontiers in Neuroscience, 2019. **13**.
76. Manuel Matamala, J., et al., *Genome-wide circulating microRNA expression profiling reveals potential biomarkers for amyotrophic lateral sclerosis.* Neurobiology of Aging, 2018. **64**: p. 123-138.
77. Villard, A., et al., *Diagnostic Value of Cell-free Circulating MicroRNAs for Obesity and Type 2 Diabetes: A Meta-analysis.* Journal of molecular biomarkers & diagnosis, 2015. **6**(6).
78. Espinosa-Parrilla, Y., et al., *Decoding the Role of Platelets and Related MicroRNAs in Aging and Neurodegenerative Disorders.* Frontiers in Aging Neuroscience, 2019. **11**.
79. Liu, J., et al., *Identification of Differentially Expressed miRNAs in the Response of Spleen CD4(+) T Cells to Electroacupuncture in Senescence-Accelerated Mice.* Cell Biochemistry and Biophysics, 2020. **78**(1): p. 89-100.
80. Zago, E., et al., *Early downregulation of hsa-miR-144-3p in serum from drug-naive Parkinson's disease patients.* Scientific Reports, 2022. **12**(1).
81. Liu, Y., et al., *Circulating exosomal miR-144-3p inhibits the mobilization of endothelial progenitor cells post myocardial infarction via regulating the MMP9 pathway.* Aging-Us, 2020. **12**(16): p. 16294-16303.
82. Wang, W.-X., et al., *A Highly Predictive MicroRNA Panel for Determining Delayed Cerebral

Vasospasm Risk Following Aneurysmal Subarachnoid Hemorrhage. Frontiers in Molecular Biosciences, 2021. **8**.

83. Li, Y., et al., *Metabolic syndrome increases senescence-associated micro-RNAs in extracellular vesicles derived from swine and human mesenchymal stem/stromal cells (vol 18, 124, 2020).* Cell Communication and Signaling, 2020. **18**(1).
84. Dreher, S.I., et al., *TGF-beta Induction of miR-143/145 Is Associated to Exercise Response by Influencing Differentiation and Insulin Signaling Molecules in Human Skeletal Muscle.* Cells, 2021. **10**(12).
85. Sardu, C., et al., *Cardiac Resynchronization Therapy Outcomes in Type 2 Diabetic Patients: Role of MicroRNA Changes.* Journal of Diabetes Research, 2016. **2016**.
86. Ren, S., et al., *The whole profiling and competing endogenous RNA network analyses of noncoding RNAs in adipose-derived stem cells from diabetic, old, and young patients.* Stem Cell Research & Therapy, 2021. **12**(1).
87. Khor, E.-S. and P.-F. Wong, *Endothelial replicative senescence delayed by the inhibition of MTORC1 signaling involves MicroRNA-107.* International Journal of Biochemistry & Cell Biology, 2018. **101**: p. 64-73.
88. Jacobo-Albavera, L., et al., *The Role of the ATP-Binding Cassette A1 (ABCA1) in Human Disease.* International Journal of Molecular Sciences, 2021. **22**(4).
89. Nielsen, S., et al., *The miRNA Plasma Signature in Response to Acute Aerobic Exercise and Endurance Training.* Plos One, 2014. **9**(2).
90. Wei, Z.-Y.D. and A.K. Shetty, *Can mild cognitive impairment and Alzheimer's disease be diagnosed by monitoring a miRNA triad in the blood?* Aging Cell, 2022. **21**(6).
91. Hu, S., et al., *In silico analysis identifies neuropilin-1 as a potential therapeutic target for SARS-Cov-2 infected lung cancer patients.* Aging-Us, 2021. **13**(12): p. 15770-15784.
92. Goedeke, L., et al., *MicroRNA-148a regulates LDL receptor and ABCA1 expression to control circulating lipoprotein levels.* Nature Medicine, 2015. **21**(11): p. 1280-1289.
93. Guo, K., et al., *Bone morphogenetic protein 9, and its genetic variants contribute to susceptibility of idiopathic pulmonary arterial hypertension.* Aging-Us, 2020. **12**(3): p. 2123-2131.
94. Bruno, S., et al., *Renal Regenerative Potential of Different Extracellular Vesicle Populations Derived from Bone Marrow Mesenchymal Stromal Cells.* Tissue Engineering Part A, 2017. **23**(21-22): p. 1262-1273.
95. Mihanfar, A., et al., *Exosomal miRNAs in osteoarthritis.* Molecular Biology Reports, 2020. **47**(6): p. 4737-4748.
96. Pourrajab, F., et al., *The master switchers in the aging of cardiovascular system, reverse senescence by microRNA signatures; as highly conserved molecules.* Progress in Biophysics & Molecular Biology, 2015. **119**(2): p. 111-128.
97. Leoncini, P.P., et al., *MicroRNA fingerprints in juvenile myelomonocytic leukemia (JMML) identified miR-150-5p as a tumor suppressor and potential target for treatment.* Oncotarget, 2016. **7**(34): p. 55395-55408.
98. Yao, M.-Y., et al., *Long non-coding RNA MALAT1 exacerbates acute respiratory distress syndrome by upregulating ICAM-1 expression via microRNA-150-5p downregulation.* Aging-

Us, 2020. **12**(8): p. 6570-6585.

99. He, Y., et al., *MicroRNA-151a-3p Functions in the Regulation of Osteoclast Differentiation: Significance to Postmenopausal Osteoporosis.* Clinical Interventions in Aging, 2021. **16**: p. 1357-1366.

100. Mengel-From, J., et al., *Circulating microRNAs disclose biology of normal cognitive function in healthy elderly people - a discovery twin study.* European Journal of Human Genetics, 2018. **26**(9): p. 1378-1387.

101. Meng, J., et al., *Identification of miR-194-5p as a potential biomarker for postmenopausal osteoporosis.* Peerj, 2015. **3**.

102. Leggio, L., et al., *Extracellular Vesicles as Novel Diagnostic and Prognostic Biomarkers for Parkinson's Disease.* Aging and Disease, 2021. **12**(6): p. 1494-1515.

103. Daimiel, L., et al., *Alcoholic and Non-Alcoholic Beer Modulate Plasma and Macrophage microRNAs Differently in a Pilot Intervention in Humans with Cardiovascular Risk.* Nutrients, 2021. **13**(1).

104. Sanfiorenzo, C., et al., *Two Panels of Plasma MicroRNAs as Non-Invasive Biomarkers for Prediction of Recurrence in Resectable NSCLC.* Plos One, 2013. **8**(1).

105. Jung, H.J., et al., *MicroRNAs in Skeletal Muscle Aging: Current Issues and Perspectives.* Journals of Gerontology Series a-Biological Sciences and Medical Sciences, 2019. **74**(7): p. 1008-1014.

106. Yuan, Y., et al., *Role of microRNA-15a in autoantibody production in interferon-augmented murine model of lupus.* Molecular Immunology, 2012. **52**(2): p. 61-70.

107. Ye, Z., et al., *Gene co-expression network for analysis of plasma exosomal miRNAs in the elderly as markers of aging and cognitive decline.* Peerj, 2020. **8**.

108. Ristau, J., et al., *Suitability of Circulating miRNAs as Potential Prognostic Markers in Colorectal Cancer.* Cancer Epidemiology Biomarkers & Prevention, 2014. **23**(12): p. 2632-2637.

109. Dellago, H., M.R. Bobbili, and J. Grillari, *MicroRNA-17-5p: At the Crossroads of Cancer and Aging - A Mini-Review.* Gerontology, 2017. **63**(1): p. 20-28.

110. Liu, H.-W., et al., *Effect of Progressive Resistance Training on Circulating Adipogenesis-, Myogenesis-, and Inflammation-Related microRNAs in Healthy Older Adults: An Exploratory Study.* Gerontology, 2020. **66**(6): p. 562-570.

111. Kim, J.-H., et al., *Reverse Expression of Aging-Associated Molecules through Transfection of miRNAs to Aged Mice.* Molecular Therapy-Nucleic Acids, 2017. **6**: p. 106-115.

112. Giuliani, A., et al., *Circulating Inflamma-miRs as Potential Biomarkers of Cognitive Impairment in Patients Affected by Alzheimer's Disease.* Frontiers in Aging Neuroscience, 2021. **13**.

113. Noren Hooten, N., et al., *Age-related changes in microRNA levels in serum.* Aging-Us, 2013. **5**(10): p. 725-740.

114. Hori, D., et al., *miR-181b regulates vascular stiffness age dependently in part by regulating TGF-beta signaling.* Plos One, 2017. **12**(3).

115. Lai, Y., et al., *Exosome long non-coding RNA SOX2-OT contributes to ovarian cancer malignant progression by miR-181b-5p/SCD1 signaling.* Aging-Us, 2021. **13**(20): p. 23726-

23738.
116. Li, F.-J., et al., *Involvement of the MiR-181b-5p/HMGB1 Pathway in Ang II-induced Phenotypic Transformation of Smooth Muscle Cells in Hypertension.* Aging and Disease, 2019. **10**(2): p. 231-248.
117. Shi, J., et al., *MicroRNAs are potential prognostic and therapeutic targets in diabetic osteoarthritis.* Journal of Bone and Mineral Metabolism, 2015. **33**(1): p. 1-8.
118. Li, T., et al., *MiR-185 targets POT1 to induce telomere dysfunction and cellular senescence.* Aging-Us, 2020. **12**(14): p. 14791-14807.
119. Ipson, B.R., et al., *IDENTIFYING EXOSOME-DERIVED MICRORNAS AS CANDIDATE BIOMARKERS OF FRAILTY.* Journal of Frailty & Aging, 2018. **7**(2): p. 100-103.
120. Vegter, E.L., et al., *Low circulating microRNA levels in heart failure patients are associated with atherosclerotic disease and cardiovascular-related rehospitalizations.* Clinical Research in Cardiology, 2017. **106**(8): p. 598-609.
121. Vegter, E.L., et al., *Rodent heart failure models do not reflect the human circulating microRNA signature in heart failure.* Plos One, 2017. **12**(5).
122. Nevola, K.T., et al., *miRNAMechanisms Underlying the Association of Beta Blocker Use and Bone Mineral Density.* Journal of Bone and Mineral Research, 2021. **36**(1): p. 110-122.
123. Morsiani, C., et al., *Circulating miR-19a-3p and miR-19b-3p characterize the human aging process and their isomiRs associate with healthy status at extreme ages.* Aging Cell, 2021. **20**(7).
124. Xiao, Z., et al., *Blocking lncRNA H19-m R-19a-Id2 axis attenuates hypoxia/ischemia induced neuronal injury.* Aging-Us, 2019. **11**(11): p. 3585-3600.
125. Gou, L., et al., *Inhibition of Exo-miR-19a-3p derived from cardiomyocytes promotes angiogenesis and improves heart function in mice with myocardial infarction via targeting HIF-1 alpha.* Aging-Us, 2020. **12**(23): p. 23609-23618.
126. Soares, E., et al., *Circulating Extracellular Vesicles: The Missing Link between Physical Exercise and Depression Management?* International Journal of Molecular Sciences, 2021. **22**(2).
127. Zhou, Q., et al., *Circulating microRNAs in Response to Exercise Training in Healthy Adults.* Frontiers in Genetics, 2020. **11**.
128. Liu, C., et al., *Research Paper Genetic predisposition and bioinformatics analysis of ATP-sensitive potassium channels polymorphisms with the risks of elevated apolipoprotein B serum levels and its related arteriosclerosis cardiovascular disease.* Aging-Us, 2021. **13**(6): p. 8177-8203.
129. Murabito, J.M., et al., *Cross-sectional relations of whole-blood miRNA expression levels and hand grip strength in a community sample.* Aging Cell, 2017. **16**(4): p. 888-894.
130. Bugden, M., et al., *Ionizing radiation affects miRNA composition in both young and old mice.* International Journal of Radiation Biology, 2019. **95**(10): p. 1404-1413.
131. Li, J., et al., *Circulating non-coding RNA cluster predicted the tumorigenesis and development of colorectal carcinoma.* Aging-Us, 2020. **12**(22): p. 23047-23066.
132. Flowers, E., et al., *Circulating microRNA-320a and microRNA-486 predict thiazolidinedione response: Moving towards precision health for diabetes prevention.* Metabolism-Clinical and Experimental, 2015. **64**(9): p. 1051-1059.

133. Ma, Y., et al., *Serum miRNA expression and correlation with clinical characteristics in acute kidney injury.* International Journal of Clinical and Experimental Pathology, 2017. **10**(8): p. 8721-8726.

134. Balzano, F., et al., *MicroRNA Expression Analysis of Centenarians and Rheumatoid Arthritis Patients Reveals a Common Expression Pattern.* International Journal of Medical Sciences, 2017. **14**(7): p. 622-628.

135. Cha, D.J., et al., *miR-212 and miR-132 Are Downregulated in Neurally Derived Plasma Exosomes of Alzheimer's Patients.* Frontiers in Neuroscience, 2019. **13**.

136. Schulz, et al., *Meta-analyses identify differentially expressed micrornas in Parkinson's disease.*

137. Li, D., et al., *Osteoclast-derived exosomal miR-214-3p inhibits osteoblastic bone formation.* Nature Communications, 2016. **7**.

138. Nakamura, N., et al., *Increased estrogen levels altered microRNA expression in prostate and plasma of rats dosed with sex hormones.* Andrology, 2020. **8**(5): p. 1360-1374.

139. Ouyang, Y., et al., *MiR-21-5p/dual-specificity phosphatase 8 signalling mediates the anti-inflammatory effect of haem oxygenase-1 in aged intracerebral haemorrhage rats.* Aging Cell, 2019. **18**(6).

140. Shen, Y., et al., *Role of long non-coding RNA MIAT in proliferation, apoptosis and migration of lens epithelial cells: a clinical and in vitro study.* Journal of Cellular and Molecular Medicine, 2016. **20**(3): p. 537-548.

141. Wu, F., et al., *Exosomes increased angiogenesis in papillary thyroid cancer microenvironment.* Endocrine-Related Cancer, 2019. **26**(5): p. 525-538.

142. Xu, J., et al., *Evaluation of the cargo contents and potential role of extracellular vesicles in osteoporosis.* Aging-Us, 2021. **13**(15): p. 19282-19292.

143. Zhang, J.-J., et al., *Atorvastatin exerts inhibitory effect on endothelial senescence in hyperlipidemic rats through a mechanism involving down-regulation of miR-21-5p/203a-3p.* Mechanisms of Ageing and Development, 2018. **169**: p. 10-18.

144. de Yebenes, V.G., et al., *Aging-Associated miR-217 Aggravates Atherosclerosis and Promotes Cardiovascular Dysfunction.* Arteriosclerosis Thrombosis and Vascular Biology, 2020. **40**(10): p. 2408-2424.

145. Florio, M.C., et al., *Aging, MicroRNAs, and Heart Failure.* Current Problems in Cardiology, 2020. **45**(12).

146. Zheng, Y. and Z. Xu, *MicroRNA-22 Induces Endothelial Progenitor Cell Senescence by Targeting AKT3.* Cellular Physiology and Biochemistry, 2014. **34**(5): p. 1547-1555.

147. Lacolley, P., et al., *The vascular smooth muscle cell in arterial pathology: a cell that can take on multiple roles.* Cardiovascular Research, 2012. **95**(2): p. 194-204.

148. Maekitie, R.E., et al., *Altered microRNA profile in osteoporosis caused by impaired WNT signaling.* European Journal of Human Genetics, 2019. **27**: p. 629-629.

149. Satoh, M., et al., *Expression of miR-23a induces telomere shortening and is associated with poor clinical outcomes in patients with coronary artery disease.* Clinical Science, 2017. **131**(15): p. 2007-2017.

150. Monaghan, T.M., et al., *Fecal Microbiota Transplantation for Recurrent Clostridioides difficile Infection Associates With Functional Alterations in Circulating microRNAs.* Gastroenterology,

2021. **161**(1): p. 255-+.
151. Mastropasqua, R., et al., *Serum microRNA Levels in Diabetes Mellitus.* Diagnostics, 2021. **11**(2).
152. Smit-McBride, Z., et al., *Effects of aging and environmental tobacco smoke exposure on ocular and plasma circulatory microRNAs in the Rhesus macaque.* Molecular Vision, 2018. **24**: p. 633-646.
153. Zhan, L., et al., *Protective role of miR-23b-3p in kainic acid-induced seizure.* Neuroreport, 2016. **27**(10): p. 764-768.
154. Li, H., et al., *Bioinformatics analysis of differentially expressed genes and identification of an miRNA–mRNA network associated with entorhinal cortex and hippocampus in Alzheimer's disease.* Hereditas, 2021. **158**(1): p. 25.
155. Vegter, E.L.E., et al., *Rodent heart failure models do not reflect the human circulating microRNA response in heart failure.* European Journal of Heart Failure, 2017. **19**: p. 334-334.
156. Bonafè, M. and F. Olivieri, *Circulating microRNAs in aging.* Oncotarget, 2015. **6**(3): p. 1340-1341.
157. Fazeli, S., et al., *A compound downregulation of SRRM2 and miR-27a-3p with upregulation of miR-27b-3p in PBMCs of Parkinson's patients is associated with the early stage onset of disease (vol 15, e0240855, 2020).* Plos One, 2020. **15**(12).
158. Zhang, T., et al., *Circulating MiRNAs as biomarkers of gait speed responses to aerobic exercise training in obese older adults.* Aging-Us, 2017. **9**(3): p. 900-913.
159. Xu, W., et al., *CeRNA regulatory network-based analysis to study the roles of noncoding RNAs in the pathogenesis of intrahepatic cholangiocellular carcinoma.* Aging-Us, 2020. **12**(2): p. 1047-1086.
160. Kangas, R., et al., *Aging and serum exomiR content in women-effects of estrogenic hormone replacement therapy.* Scientific Reports, 2017. **7**.
161. Meadows, S., et al., *Altered Regulation of adipomiR Editing with Aging.* International Journal of Molecular Sciences, 2020. **21**(18).
162. Han, L., et al., *Association of the serum microRNA-29 family with cognitive impairment in Parkinson's disease.* Aging-Us, 2020. **12**(13): p. 13518-13528.
163. Wang, X. and K. Hong, *Atrial remodeling and atrial fibrillation: recent advances and translational perspectives.* Chinese Journal of Geriatrics, 2016. **35**(5): p. 560-564.
164. Yavropoulou, M.P., et al., *Serum Profile of microRNAs Linked to Bone Metabolism During Sequential Treatment for Postmenopausal Osteoporosis.* Journal of Clinical Endocrinology & Metabolism, 2020. **105**(8): p. E2885-E2894.
165. Cui, Y., et al., *MiR-29a-3p Improves Acute Lung Injury by Reducing Alveolar Epithelial Cell PANoptosis.* Aging and Disease, 2021.
166. Yang, Y., et al., *Circulating MicroRNAs and Long Non-coding RNAs as Potential Diagnostic Biomarkers for Parkinson's Disease.* Frontiers in Molecular Neuroscience, 2021. **14**.
167. Geekiyanage, H., A. Upadhye, and C. Chan, *Inhibition of serine palmitoyltransferase reduces A beta and tau hyperphosphorylation in a murine model: a safe therapeutic strategy for Alzheimer's disease.* Neurobiology of Aging, 2013. **34**(8): p. 2037-2051.
168. Bake, S., et al., *Blood Brain Barrier and Neuroinflammation Are Critical Targets of IGF-1-Mediated Neuroprotection in Stroke for Middle-Aged Female Rats.* Plos One, 2014. **9**(3).

169. Wardzynska, A., et al., *Circulating MicroRNAs and T-Cell Cytokine Expression Are Associated With the Characteristics of Asthma Exacerbation.* Allergy Asthma & Immunology Research, 2020. **12**(1): p. 125-136.
170. Feichtinger, X., et al., *Bone-related Circulating MicroRNAs miR-29b-3p, miR-550a-3p, and miR-324-3p and their Association to Bone Microstructure and Histomorphometry.* Scientific Reports, 2018. **8**.
171. Yang, C.-P., et al., *Muscle atrophy-related myotube-derived exosomal microRNA in neuronal dysfunction: Targeting both coding and long noncoding RNAs.* Aging Cell, 2020. **19**(5).
172. Kocijan, R., et al., *Circulating microRNA Signatures in Patients With Idiopathic and Postmenopausal Osteoporosis and Fragility Fractures.* Journal of Clinical Endocrinology & Metabolism, 2016. **101**(11): p. 4125-4134.
173. Li, H., et al., *Bioinformatics analysis of differentially expressed genes and identification of an miRNA-mRNA network associated with entorhinal cortex and hippocampus in Alzheimer's disease.* Hereditas, 2021. **158**(1).
174. Jimenez-Lucena, R., et al., *A PLASMA CIRCULATING MIRNAS PROFILE PREDICTS TYPE 2 DIABETES MELLITUS AND PREDIABETES: FROM THE CORDIOPREV STUDY.* Atherosclerosis, 2018. **275**: p. E5-E5.
175. Yang, B., et al., *Tumor-derived exosomal circRNA_102481 contributes to EGFR-TKIs resistance via the miR-30a-5p/ROR1 axis in non-small cell lung cancer.* Aging-Us, 2021. **13**(9): p. 13264-13286.
176. Jimenez-Lucena, R., et al., *Circulating miRNAs as Predictive Biomarkers of Type 2 Diabetes Mellitus Development in Coronary HeartDisease Patients from the CORDIOPREV Study.* Molecular Therapy-Nucleic Acids, 2018. **12**: p. 146-157.
177. Alberto Rangel-Zuniga, O., et al., *A set of miRNAs predicts T2DM remission in patients with coronary heart disease: from the CORDIOPREV study.* Molecular Therapy-Nucleic Acids, 2021. **23**: p. 255-263.
178. Capri, M., et al., *Identification of miR-31-5p, miR-141-3p, miR-200c-3p, and GLT1 as human liver aging markers sensitive to donor-recipient age-mismatch in transplants.* Aging Cell, 2017. **16**(2): p. 262-272.
179. Heilmeier, U., et al., *Circulating serum microRNAs including senescent miR-31-5p are associated with incident fragility fractures in older postmenopausal women with type 2 diabetes mellitus.* Bone, 2022. **158**.
180. Shin, P.-K., et al., *A Traditional Korean Diet Alters the Expression of Circulating MicroRNAs Linked to Diabetes Mellitus in a Pilot Trial.* Nutrients, 2020. **12**(9).
181. Wakabayashi, I., et al., *Blood levels of microRNAs associated with ischemic heart disease differ between Austrians and Japanese: a pilot study.* Scientific Reports, 2020. **10**(1).
182. Santovito, D., et al., *Plasma microRNA signature associated with retinopathy in patients with type 2 diabetes.* Scientific Reports, 2021. **11**(1).
183. Dalmasso, B., et al., *Age-related microRNAs in older breast cancer patients: biomarker potential and evolution during adjuvant chemotherapy.* Bmc Cancer, 2018. **18**.
184. Giuliani, A., et al., *Circulating miR-320b and miR-483-5p levels are associated with COVID-19 in-hospital mortality.* Mechanisms of Ageing and Development, 2022. **202**.

185. Kenis, C., et al., *Circulating MicroRNAs as Easy-to-Measure Aging Biomarkers in Older Breast Cancer Patients: Correlation with Chronological Age but Not with Fitness/Frailty Status*. 2014, Figshare.
186. Simonson, O.E., et al., *In Vivo Effects of Mesenchymal Stromal Cells in Two Patients With Severe Acute Respiratory Distress Syndrome (vol 4, pg 1199, 2015).* Stem Cells Translational Medicine, 2016. **5**(6): p. 845-845.
187. Fletcher, M., et al., *Identifying exosome-derived microRNAs as candidate biomarkers of frailty.* Journal of the American Geriatrics Society, 2017. **65**: p. S121-S122.
188. Wei, C., et al., *MicroRNA-330-3p promotes brain metastasis and epithelial-mesenchymal transition via GRIA3 in non-small cell lung cancer.* Aging-Us, 2019. **11**(17): p. 6734-6761.
189. Fonseca, A., et al., *Identification of colorectal cancer associated biomarkers: an integrated analysis of miRNA expression (vol 13, pg 21991, 2021).* Aging-Us, 2022. **14**(4): p. 2014-2015.
190. Reynoso, R., et al., *MicroRNAs differentially present in the plasma of HIV elite controllers reduce HIV infection in vitro.* Scientific Reports, 2014. **4**.
191. Rodriguez-Ayala, E., et al., *Towards precision medicine: defining and characterizing adipose tissue dysfunction to identify early immunometabolic risk in symptom-free adults from the GEMM family study.* Adipocyte, 2020. **9**(1): p. 153-169.
192. Song, Q., et al., *miR-33a-5p inhibits the progression of esophageal cancer through the DKK1-mediatea Wnt/beta-catenin pathway.* Aging-Us, 2021. **13**(16): p. 20481-20494.
193. Corona-Meraz, F.-I., et al., *Ageing influences the relationship of circulating miR-33a and miR-33b levels with insulin resistance and adiposity.* Diabetes & Vascular Disease Research, 2019. **16**(3): p. 244-253.
194. Mandolini, C., et al., *Identification of microRNAs 758 and 33b as potential modulators of ABCA1 expression in human atherosclerotic plaques.* Nutrition Metabolism and Cardiovascular Diseases, 2015. **25**(2): p. 202-209.
195. Goodall, E.F., et al., *Age-Associated mRNA and miRNA Expression Changes in the Blood-Brain Barrier.* International Journal of Molecular Sciences, 2019. **20**(12).
196. Freiesleben, S., et al., *Analysis of microRNA and Gene Expression Profiles in Multiple Sclerosis: Integrating Interaction Data to Uncover Regulatory Mechanisms.* Scientific Reports, 2016. **6**.
197. Zhao, X., et al., *A Machine Learning Approach to Identify a Circulating MicroRNA Signature for Alzheimer Disease.* Journal of Applied Laboratory Medicine, 2020. **5**(1): p. 15-28.
198. Owczarz, M., et al., *Age-related epigenetic drift deregulatesSIRT6expression and affects its downstream genes in human peripheral blood mononuclear cells.* Epigenetics, 2020. **15**(12): p. 1336-1347.
199. MacDonagh, L., et al., *MicroRNA expression profiling and biomarker validation in treatment-naive and drug resistant non-small cell lung cancer.* Translational Lung Cancer Research, 2021. **10**(4): p. 1773-+.
200. Haluck-Kangas, A., et al., *DISE/6mer seed toxicity-a powerful anti-cancer mechanism with implications for other diseases.* Journal of Experimental & Clinical Cancer Research, 2021. **40**(1).
201. Forte, D., et al., *Distinct profile of CD34(+) cells and plasma-derived extracellular vesicles from triple-negative patients with Myelofibrosis reveals potential markers of aggressive disease.*

Journal of Experimental & Clinical Cancer Research, 2021. **40**(1).

202. Nambiar, A.M., et al., *Senolytics in Idiopathic Pulmonary Fibrosis: Preliminary Results from a First-in-Human, Open-Label, Pilot Study.* American Journal of Respiratory and Critical Care Medicine, 2019. **199**.

203. Kanata, E., et al., *MicroRNA Alterations in the Brain and Body Fluids of Humans and Animal Prion Disease Models: Current Status and Perspectives.* Frontiers in Aging Neuroscience, 2018. **10**.

204. Prabhakar, P., S.R. Chandra, and R. Christopher, *Circulating microRNAs as potential biomarkers for the identification of vascular dementia due to cerebral small vessel disease.* Age and Ageing, 2017. **46**(5): p. 861-864.

205. Liu, D., et al., *Cross-platform genomic identification and clinical validation of breast cancer diagnostic biomarkers.* Aging-Us, 2021. **13**(3): p. 4258-4273.

206. Kumar, S. and P.H. Reddy, *MicroRNA-455-3p as a potential peripheral biomarker and therapeutic target for Alzheimer's disease.* Journal of Alzheimers Disease, 2019. **72**: p. S32-S33.

207. Kou, X., D. Chen, and N. Chen, *Physical Activity Alleviates Cognitive Dysfunction of Alzheimer's Disease through Regulating the mTOR Signaling Pathway.* International Journal of Molecular Sciences, 2019. **20**(7).

208. Li, X.-H., et al., *H2S regulates endothelial nitric oxide synthase protein stability by promoting microRNA-455-3p expression.* Scientific Reports, 2017. **7**.

209. Nair, V.D., et al., *Sedentary and Trained Older Men Have Distinct Circulating Exosomal microRNA Profiles at Baseline and in Response to Acute Exercise.* Frontiers in Physiology, 2020. **11**.

210. Ma, J., et al., *Circulating miR-181c-5p and miR-497-5p Are Potential Biomarkers for Prognosis and Diagnosis of Osteoporosis.* Journal of Clinical Endocrinology & Metabolism, 2020. **105**(5).

211. Hou, Z., et al., *Pseudogene KRT17P3 drives cisplatin resistance of human NSCLC cells by modulating miR-497-5p/mTOR.* Cancer Science, 2021. **112**(1): p. 275-286.

212. He, N., et al., *Circulating MicroRNAs in Plasma Decrease in Response to Sarcopenia in the Elderly.* Frontiers in Genetics, 2020. **11**.

213. Gullett, J.M., et al., *MicroRNA predicts cognitive performance in healthy older adults.* Neurobiology of Aging, 2020. **95**: p. 186-194.

214. Toyama, K., M. Mogi, and P.S. Tsao, *microRNA-based biomarker for dementia.* Aging-Us, 2019. **11**(5): p. 1329-1330.

215. Toyama, K., et al., *Exosome miR-501-3p Elevation Contributes to Progression of Vascular Stiffness.* Circulation reports, 2021. **3**(3): p. 170-177.

216. Williams, J., et al., *Are microRNAs true sensors of ageing and cellular senescence?* Ageing Research Reviews, 2017. **35**: p. 350-363.

217. Cosin-Tomas, M., et al., *Temporal Integrative Analysis of mRNA and microRNAs Expression Profiles and Epigenetic Alterations in Female SAMP8, a Model of Age-Related Cognitive Decline.* Frontiers in Genetics, 2018. **9**.

218. Park, E.J., M. Shimaoka, and H. Kiyono, *Functional Flexibility of Exosomes and MicroRNAs of Intestinal Epithelial Cells in Affecting Inflammation.* Frontiers in Molecular Biosciences, 2022. **9**.

219. Souza, V.C., et al., *Whole-Blood Levels of MicroRNA-9 Are Decreased in Patients With Late-Onset Alzheimer Disease.* American Journal of Alzheimers Disease and Other Dementias, 2020. **35**.

220. Fossati, S., et al., *Ambient Particulate Air Pollution and MicroRNAs in Elderly Men.* Epidemiology, 2014. **25**(1): p. 68-78.

221. Lucas, T., et al., *Light-inducible antimiR-92a as a therapeutic strategy to promote skin repair in healing-impaired diabetic mice.* Nature Communications, 2017. **8**.

222. Chen, Z., et al., *Oxidative Stress Activates Endothelial Innate Immunity via Sterol Regulatory Element Binding Protein 2 (SREBP2) Transactivation of MicroRNA-92a.* Circulation, 2015. **131**(9): p. 805-U125.

223. Saenz-Pipaon, G., et al., *The Role of Circulating Biomarkers in Peripheral Arterial Disease.* International Journal of Molecular Sciences, 2021. **22**(7): p. 3601.

224. Wang, W., et al., *Extracellular vesicles extracted from young donor serum attenuate inflammaging via partially rejuvenating aged T-cell immunotolerance.* Faseb Journal, 2018. **32**(11): p. 5899-5912.
