# Supplementary material for: Small extracellular vesicles from young plasma reverse age-related functional declines by improving mitochondrial energy metabolism
Source: Nat Aging. 2024 Apr 16;4(6):814–38. doi: 10.1038/s43587-024-00612-4 (PMC11186790; doi:10.1038/s43587-024-00612-4)
Supplement: Supplementary file 2 — Reporting Summary [file 43587_2024_612_MOESM2_ESM.pdf]

nature research

Corresponding author(s): Xi Chen

Last updated by author(s): Feb 22, 2024

Reporting Summary

Nature Research wishes to improve the reproducibility of the work that we publish. This form provides structure for consistency and transparency in reporting. For further information on Nature Research policies, see Authors & Referees and the Editorial Policy Checklist.

Statistics

For all statistical analyses, confirm that the following items are present in the figure legend, table legend, main text, or Methods section.

n/a	Confirmed	
☐	☒	The exact sample size (*n*) for each experimental group/condition, given as a discrete number and unit of measurement
☐	☒	A statement on whether measurements were taken from distinct samples or whether the same sample was measured repeatedly
☐	☒	The statistical test(s) used AND whether they are one- or two-sided<br>*Only common tests should be described solely by name; describe more complex techniques in the Methods section.*
☒	☐	A description of all covariates tested
☐	☒	A description of any assumptions or corrections, such as tests of normality and adjustment for multiple comparisons
☐	☒	A full description of the statistical parameters including central tendency (e.g. means) or other basic estimates (e.g. regression coefficient) AND variation (e.g. standard deviation) or associated estimates of uncertainty (e.g. confidence intervals)
☐	☒	For null hypothesis testing, the test statistic (e.g. *F*, *t*, *r*) with confidence intervals, effect sizes, degrees of freedom and *P* value noted<br>*Give P values as exact values whenever suitable.*
☒	☐	For Bayesian analysis, information on the choice of priors and Markov chain Monte Carlo settings
☒	☐	For hierarchical and complex designs, identification of the appropriate level for tests and full reporting of outcomes
☒	☐	Estimates of effect sizes (e.g. Cohen's *d*, Pearson's *r*), indicating how they were calculated

Our web collection on statistics for biologists contains articles on many of the points above.

Software and code

Policy information about availability of computer code

Data collection

The images of appearance, implantation sites and the number of mice per litter were captured by camera (Cannon, Japan). Measurement of heat production, physical activity, O2 consumption and CO2 production were continuously monitored using a combined indirect calorimetry system (TSE phenoMaster, TSE Systems GmbH, Bad Homburg, Germany). Echocardiography was measured by a Visual Sonics Vevo 3100 (Fujifilm, Toronto, ON, Canada). Micro-CT images were scanned using a Hiscan XM Micro CT analyzer (Suzhou Hiscan Information Technology). MRI images were scanned using 9.4 T BioSpec 94/20 USR animal scanner (Bruker, Germany). Contextual fear conditioning test was recorded using a video tracking system and software, FCT-100 (Taimeng, Chengdu, China). The images of SA-β-gal staining, SDH staining, and Lipofuscin staining were captured using a microscope (Nikon, Japan). The protein level was visualized using the Tanon 5200 Multi detection system (Tanon, Shanghai, China). The EdU stained cells were imaged using a BX51 fluorescence microscope (Olympus, Japan). MS data were acquired with a TripleTOF 5600+ System (AB SCIEX, MA, USA). The images of mitochondrial ultrastructure and sEV were captured using a Hitachi TEM system (Hitachi HT7700) at 80.0 kV. Small RNA libraries were constructed according to the TruSeq Small RNA Sample Prep Kit (Illumina). Sperm DNA fragmentation was captured with a bright-field microscope (Nikon, Japan).

Data analysis

Micro-CT 3D-images were reconstructed with Hiscan Reconstruct software (Version 3.0, Suzhou Hiscan Information Technology) and analyzed with Hiscan Analyzer software (Version 3.0, Suzhou Hiscan Information Technology). MRI images, including the whole brain, hippocampus and cortex, were analyzed with the professional 3D medical image segment software ITK-SNAP 3.8.0 package (University of Pennsylvania, USA). Contextual fear conditioning test was analyzed using a video tracking system and software, FCT-100 (Taimeng, Chengdu, China). The images of SDH staining and western blot data were quantified using ImageJ 1.46 software (NIH, Bethesda, MD). The OCR was analyzed using an XF96 Analyzer (Seahorse Bioscience, Copenhagen, Denmark). The size and concentration of sEVs were analyzed with a NanosightLM10 system equipped with a blue laser (405 nm). The evaluation of sperm motility parameters was conducted using a computer-assisted sperm analysis system (CASA) with Sperm Vision HR software version 1.01 (Minitube, Ingersoll, ON, Canada). All statistical tests were performed using GraphPad Prism software Version 8 (San Diego, CA) or the open source statistical package R. All data are presented as the means ± SEM. Significance was analyzed using one-way ANOVA or unpaired two-tailed Student's t test, and $P < 0.05$ was considered statistically significant. In-depth functional class scoring (FCS) was performed using gene set enrichment analysis software (GSEA, http://www.gsea-msigdb.org). The GO terms of GSEA were clustered with binary cut using the R

package simplifyEnrichment to further analyze the GSEA results. Mature miRNA sequences were obtained from miRBase v21 (https://mirbase.org/).

For manuscripts utilizing custom algorithms or software that are central to the research but not yet described in published literature, software must be made available to editors/reviewers. We strongly encourage code deposition in a community repository (e.g. GitHub). See the Nature Research guidelines for submitting code & software for further information.

Data

Policy information about availability of data

All manuscripts must include a data availability statement. This statement should provide the following information, where applicable:

- Accession codes, unique identifiers, or web links for publicly available datasets
- A list of figures that have associated raw data
- A description of any restrictions on data availability

The original MS/MS data were submitted to ProteinPilot Software (version 4.5, AB SCIEX, MA, USA) for data analysis and searched against Mus musculus sequences in the UniProt database concatenated with the reverse decoy database (March 4, 2021, containing 55,366 sequences, http://www.UniProt.org/proteomes/UP000000589). The mass spectrometry proteomics data have been deposited to the ProteomeXchange Consortium via the PRIDE partner repository with the project accession PXD030350. Reviewer account details: username: reviewer_pxd030350@ebi.ac.uk; password: l41MYrFB.Quantitative data that support the findings of this study are available within the paper and the Supplementary Information. All other data that support the findings of this study are available from the corresponding author on reasonable request.

Field-specific reporting

Please select the one below that is the best fit for your research. If you are not sure, read the appropriate sections before making your selection.

☒ Life sciences ☐ Behavioural & social sciences ☐ Ecological, evolutionary & environmental sciences

For a reference copy of the document with all sections, see nature.com/documents/nr-reporting-summary-flat.pdf

Life sciences study design

All studies must disclose on these points even when the disclosure is negative.

Sample size	The sample sizes chosen are consistent with previously published works in the field.
Data exclusions	No data was excluded from the analyses.
Replication	For metabolic cage studies, echocardiography, micro-CT scanning, MRI scanning, quantitative RT-PCR, western blot, immunostaining, and RNA sequencing, experiments were conducted at least three times. All attempts at replication were successful.
Randomization	Aged animals were numbered and randomly divided into groups according to a random number table. One group is used for young sEV injection, and the other group is used as control group.
Blinding	All the investigators were blinded to group allocation during data collection and analysis.

Reporting for specific materials, systems and methods

We require information from authors about some types of materials, experimental systems and methods used in many studies. Here, indicate whether each material, system or method listed is relevant to your study. If you are not sure if a list item applies to your research, read the appropriate section before selecting a response.

Materials & experimental systems

n/a	Involved in the study	
☐	☒	Antibodies
☐	☒	Eukaryotic cell lines
☒	☐	Palaeontology
☐	☒	Animals and other organisms
☒	☐	Human research participants
☒	☐	Clinical data

Methods

n/a	Involved in the study	
☒	☐	ChIP-seq
☒	☐	Flow cytometry
☒	☐	MRI-based neuroimaging

Antibodies

Antibodies used	The detailed information of all antibodies used in the study: NDUFA9 (Thermo Fisher, 459100, 1:1000 dilituion for WB, lot number: TD2536591), ATPase-α (Thermo Fisher, 459240, 1:1000 dilituion for WB, lot number: TE2563181), CS (Santa Cruz, sc-390693, 1:1000 dilituion for WB, lot number: H2714), Cyto-c (BD Biosciences, 556433, 1:1000 dilituion for WB, lot number: 1068185), PARP2 (Santa Cruz, Sc-393343, 1:1000 dilituion for WB, lot number: J0713), mt-ATP6 (Abcam, ab192423,1:1000

	dilituion for WB, lot number: GR3198216-11), PGC1 alpha (Abcam, ab54481, 1:1000 dilituion for WB, lot number: GR3315850-1), P21 (Abcam, ab188224, 1:1000 dilituion for WB, lot number: GR3289181-4), MAP2 (Servicebio, GB11128-2, 1:1000 dilituion for IF, lot number: AC220511024), Desmin (Proteintech, 16520-1-AP, 1:500 dilituion for IF, lot number: 00099060), HIF1AN/FIH-1 (Abcam, ab92304,1:1000 dilituion for WB, lot number: GR77846-7), APP (Abcam, ab32136, 1:1000 dilituion for WB, lot number: GR3287436-6), Alix (Proteintech, 12422-1-AP, 1:2000 dilituion for WB, lot number: 00096216), TSG101 (Proteintech, 14497-1-AP, 1:1000 dilituion for WB, lot number: 00093762), CD9 (Santa Cruz, sc-13118, 1:1000 dilituion for WB, lot number: G0121), CD63 (Santa Cruz, sc-5275, 1:1000 dilituion for WB, lot number: C0320), Albumin (Proteintech, 16475-1-AP, 1:2500 dilituion for WB, lot number: 00076243), Calnexin (Santa Cruz, Sc-23954, 1:1000 dilituion for WB, lot number: AF-18), beta Actin (Servicebio, GB11001, 1:1000 dilituion for WB, lot number: LS202310), Goat anti-mouse HRP-conjugated (Santa Cruz, sc-2005, 1:1000 dilituion for WB, lot number: B1616), Goat anti-rabbit HRP-conjugated (Santa Cruz, sc-2030, 1:1000 dilituion for WB, lot number: L1015), BrdU (Abcam, ab8152, 1:100 dilituion for IHC, lot number: GR3340784-1), UltraCruz Aqueous Mounting Medium with DAPI (Santa Cruz, sc-24941, 1:300 dilituion for IHC, lot number: I1508), SDHA (Abcam, ab14715, 1:100 dilituion for IHC, lot number: 888711).
Validation	The detailed validation is listed below.<br>NDUFA9: RRID (AB_2532223)<br>ATP5A1: RRID (AB_2532234)<br>Cyto C: RRID (AB_396417)<br>CS: https://www.scbt.com/p/citrate-synthase-antibody-g-3<br>PARP-2: https://www.scbt.com/p/parp-2-antibody-f-8<br>MT-ATP6: https://www.abcam.cn/mt-atp6-antibody-ab192423.html<br>PGC1 alpha: https://www.abcam.cn/pgc1-alpha-antibody-bsa-and-azide-free-ab54481.html<br>P21: https://www.abcam.cn/p21-antibody-epr18021-ab188224.html<br>MAP2:https://www.servicebio.com/goodsdetail?id=41398<br>Desmin:https://ptgcn.com/products/DES-Antibody-16520-1-AP.htm<br>HIF1AN/FIH-1:https://www.abcam.cn/hif1anfih-1-antibody-epr3659-ab92304.html<br>APP:https://www.abcam.cn/amyloid-precursor-protein-antibody-y188-ab32136.html<br>Alix:https://www.ptgcn.com/products/PDCD6IP-Antibody-12422-1-AP.htm<br>TSG101:https://www.ptgcn.com/products/TSG101-Antibody-14497-1-AP.htm<br>CD9:https://www.scbt.com/p/cd9-antibody-c-4?requestFrom=search<br>CD63:https://www.scbt.com/p/cd63-antibody-mx-49-129-5?requestFrom=search<br>Albumin:https://ptgcn.com/products/ALB-Antibody-16475-1-AP.htm<br>Calnexin:https://www.scbt.com/p/calnexin-antibody-af18<br>beta Actin:https://www.servicebio.cn/goodsdetail?id=1308<br>BrdU:https://www.abcam.cn/brdu-antibody-iib5-ab8152.html<br>SDHA:https://www.abcam.cn/products/primary-antibodies/sdha-antibody-2e3gc12fb2ae2-ab14715.html

Eukaryotic cell lines

Policy information about cell lines

Cell line source(s)	NE-4C and C2C12 cells were provided by the Shanghai Institute of Cell Biology, Chinese Academy of Sciences .
Authentication	The STR profiling authentication procedures of NE-4C and C2C12 cells were performed by the Shanghai Institute of Cell Biology, Chinese Academy of Sciences.
Mycoplasma contamination	We confirmed that the NE-4C and C2C12 cells tested negative for mycoplasma contamination.
Commonly misidentified lines (See ICLAC register)	No commonly misidentified cell line was used in this study.

Animals and other organisms

Policy information about studies involving animals; ARRIVE guidelines recommended for reporting animal research

Laboratory animals	Male C57BL/6J mice (8-week-old, 20-month-old and 21-month-old) were used in the study. These mice were housed in a controlled environment with a 12-hour light/dark cycle (illuminated from 7 am) and granted unrestricted access to both food and water. Room temperature: 20 ~ 26°C; Daily temperature difference: ≤4°C; Relative humidity: 40% ~ 70%.
Wild animals	The study did not involve wild animals.
Field-collected samples	The study did not involve samples collected from the field.
Ethics oversight	All experiments were approved by the Animal Ethical and Welfare Committee of Nanjing University. The approved animal ethical and welfare number is IACUC-2010001.

Note that full information on the approval of the study protocol must also be provided in the manuscript.
